# Supplementary material for: Methods detecting rhythmic gene expression are biologically relevant only for strong signal
Source: PLoS Comput Biol. 2020 Mar 17;16(3):e1007666. doi: 10.1371/journal.pcbi.1007666 (PMC7100990; doi:10.1371/journal.pcbi.1007666)
Supplement: S2 File — Density distribution of p-values obtained before (raw) and after the default correction (software) for the seven methods applied to each vertebrate dataset, sub-categorized in: i. randomized data which represents the null hypothesis; ii. randomized data restricted to the first and fourth quartiles of the median gene expression level, to check for the impact of expression level under the null; iii. the full original dataset; iv. the first and fourth quartiles of the median gene expression level of the original data; and v. a subset of known cycling genes when such data was available (8 to 99 genes according to species). The default p-values of ARS, GeneCycle, and LS are uncorrected. (PDF) [file pcbi.1007666.s003.pdf]

VERTEBRATES

default *p*-values

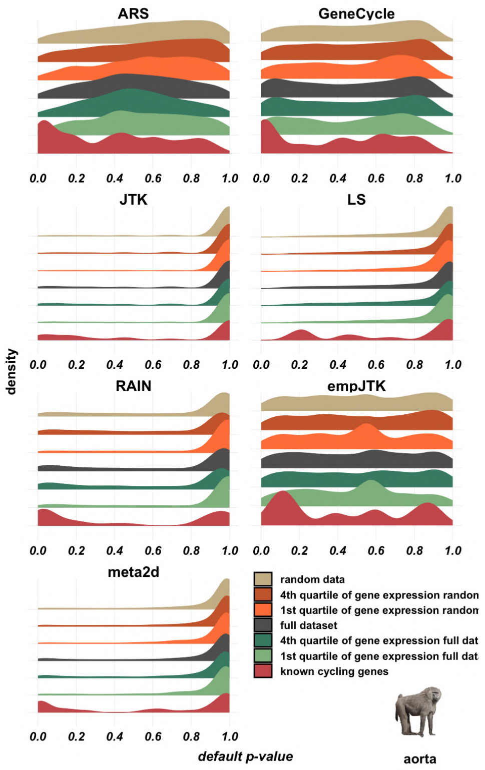

Fig. S1

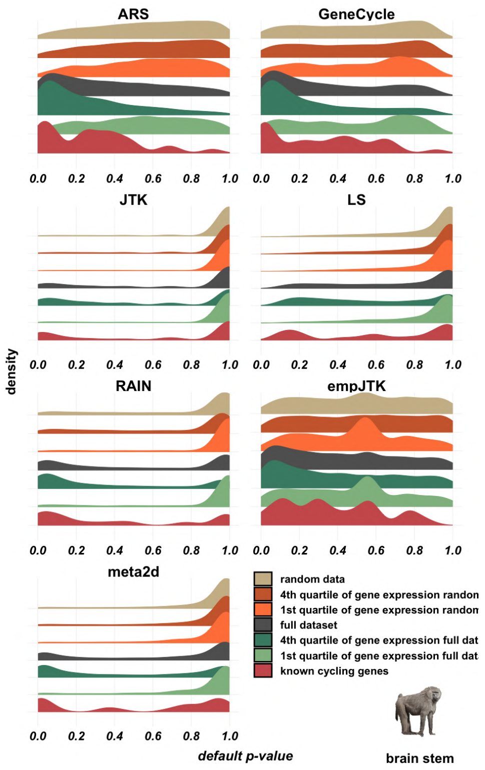

Fig. S2

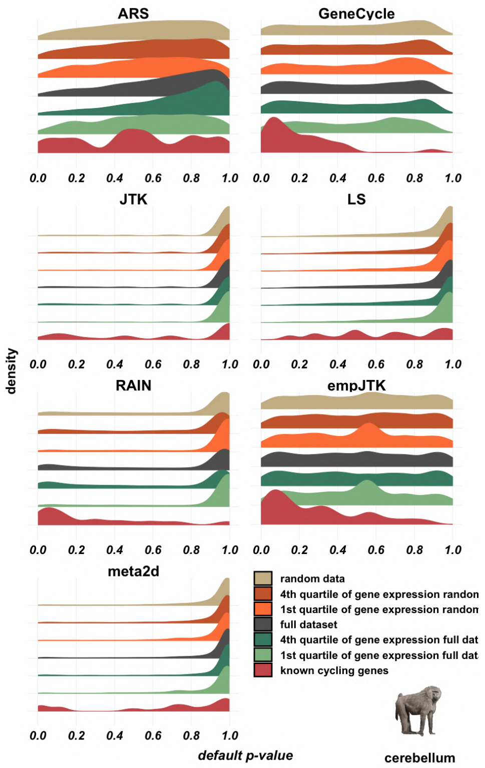
ARS
GeneCycle
JTK
LS
RAIN
empJTK
meta2d
density
0.0
0.2
0.4
0.6
0.8
1.0
default p-value
random data
4th quartile of gene expression randon
1st quartile of gene expression randon
full dataset
4th quartile of gene expression full dat
1st quartile of gene expression full dat
known cycling genes
cerebellum

Fig. S3

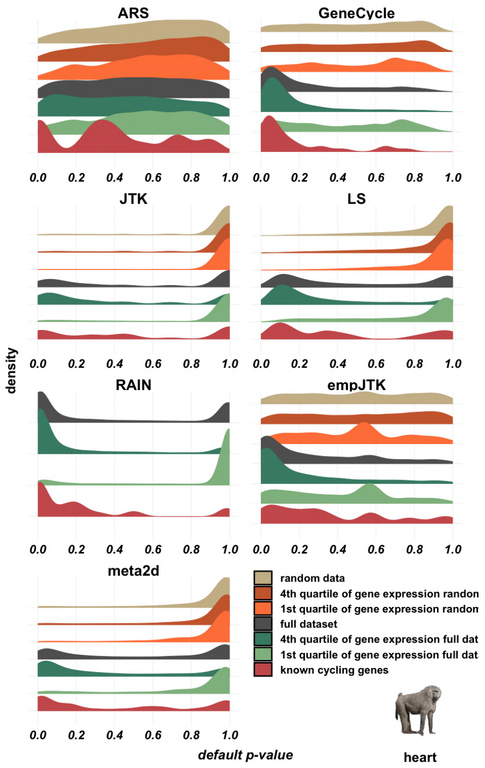
ARS
GeneCycle
JTK
LS
RAIN
empJTK
meta2d
density
0.0
0.2
0.4
0.6
0.8
1.0
default p-value
random data
4th quartile of gene expression randon
1st quartile of gene expression randon
full dataset
4th quartile of gene expression full dat
1st quartile of gene expression full dat
known cycling genes
heart

Fig. S4

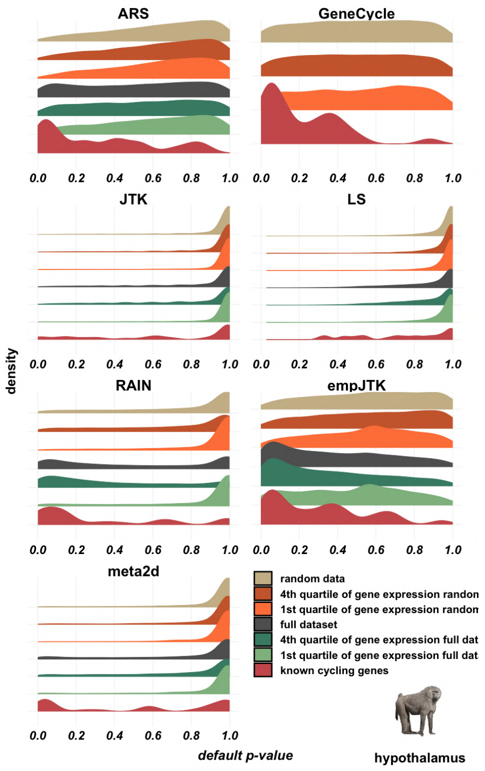
ARS
GeneCycle
JTK
LS
RAIN
empJTK
meta2d
density
default p-value
random data
4th quartile of gene expression randon
1st quartile of gene expression randon
full dataset
4th quartile of gene expression full dat
1st quartile of gene expression full dat
known cycling genes
hypothalamus

Fig. S5

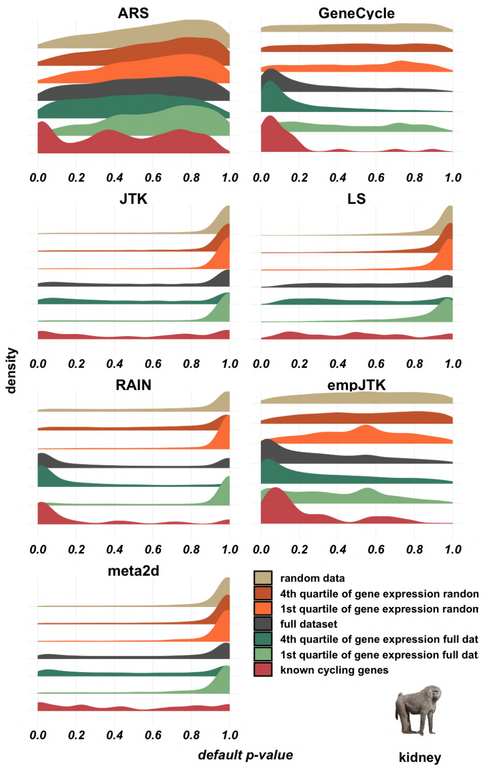
ARS
GeneCycle
JTK
LS
RAIN
empJTK
meta2d
density
default p-value
random data
4th quartile of gene expression randon
1st quartile of gene expression randon
full dataset
4th quartile of gene expression full dat
1st quartile of gene expression full dat
known cycling genes
kidney

Fig. S6

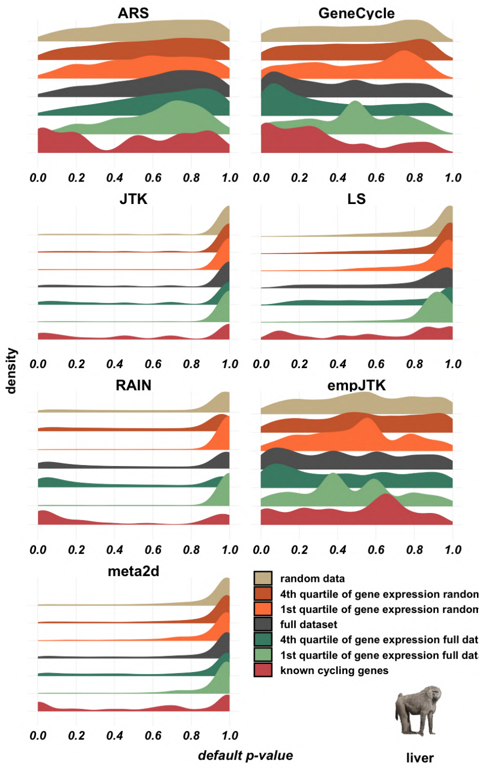
ARS
GeneCycle
JTK
LS
RAIN
empJTK
meta2d
density
0.0 0.2 0.4 0.6 0.8 1.0
default p-value
random data
4th quartile of gene expression randon
1st quartile of gene expression randon
full dataset
4th quartile of gene expression full dat
1st quartile of gene expression full dat
known cycling genes
liver

Fig. S7

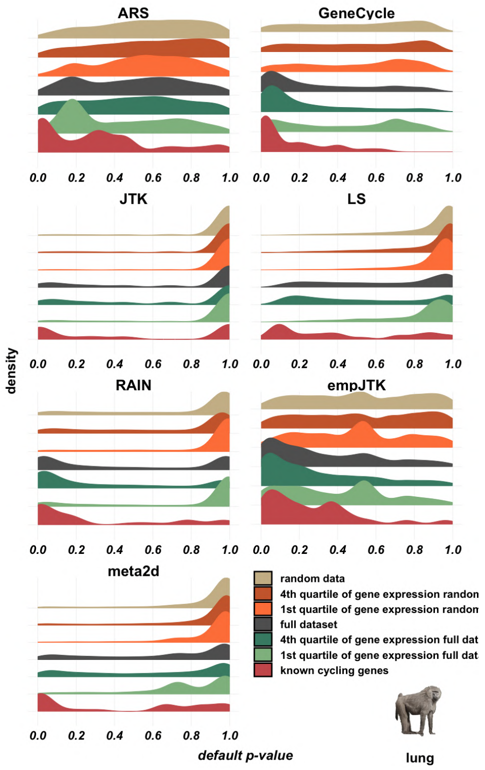
ARS
GeneCycle
JTK
LS
RAIN
empJTK
meta2d
density
0.0 0.2 0.4 0.6 0.8 1.0
default p-value
random data
4th quartile of gene expression randon
1st quartile of gene expression randon
full dataset
4th quartile of gene expression full dat
1st quartile of gene expression full dat
known cycling genes
lung

Fig. S8

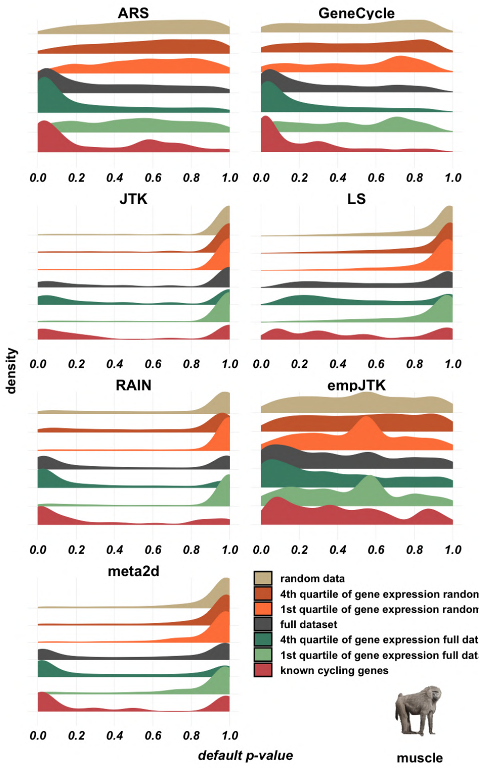

Fig. S9

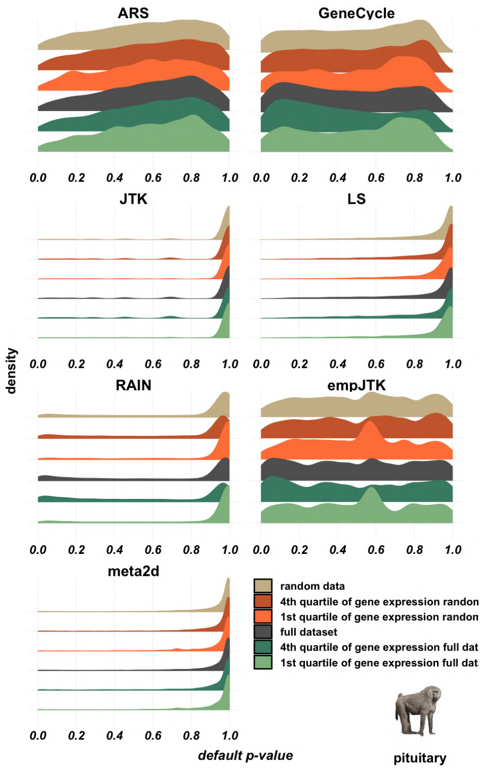

Fig. S10

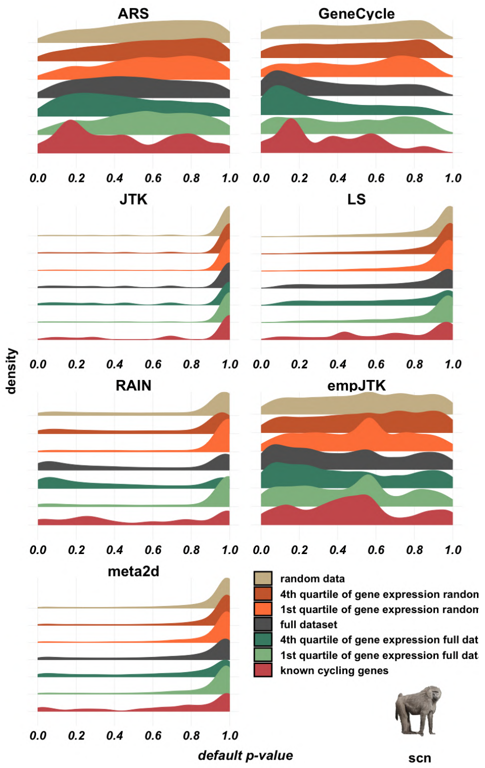

Fig. S11

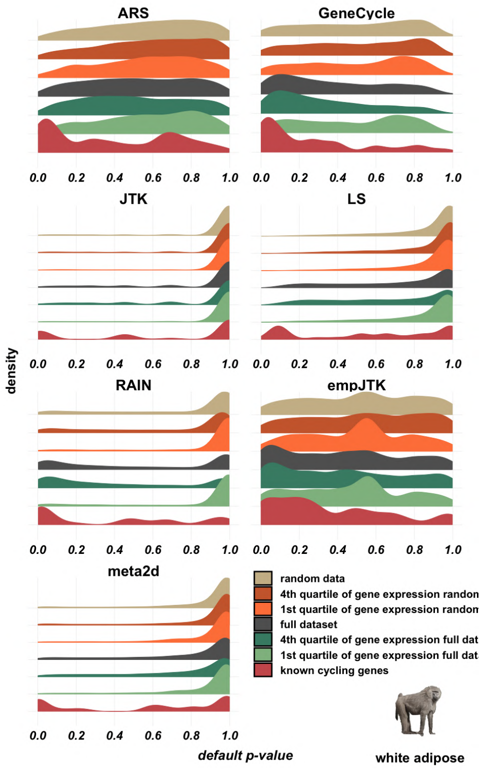

Fig. S12

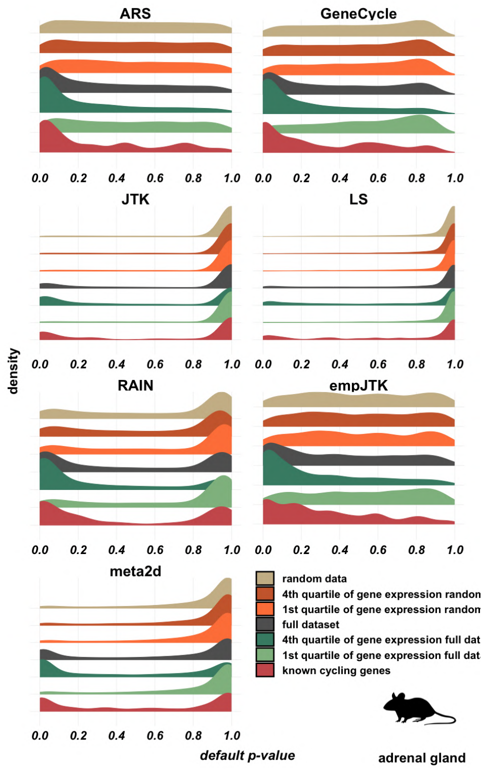

Fig. S13

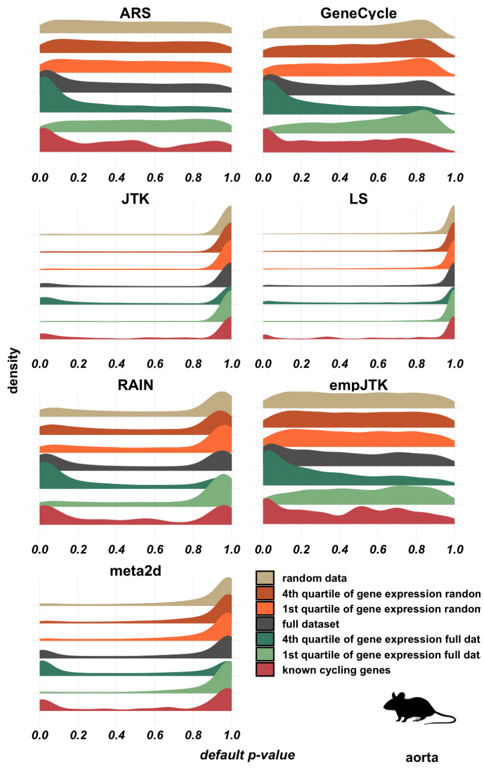

Fig. S14

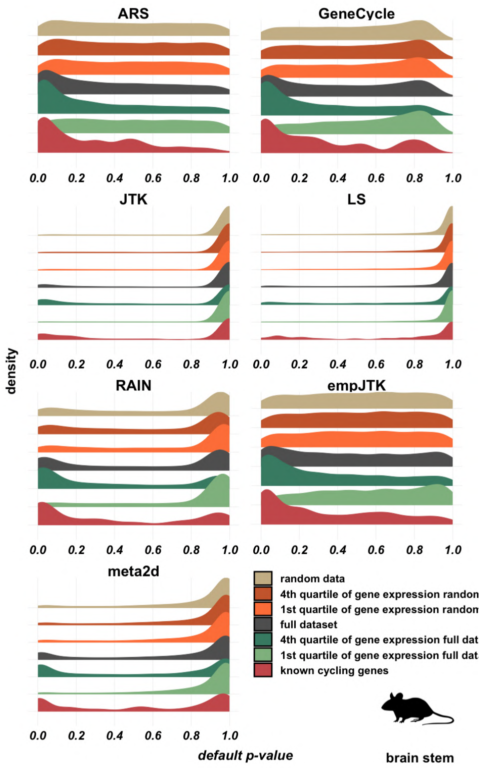

Fig. S15

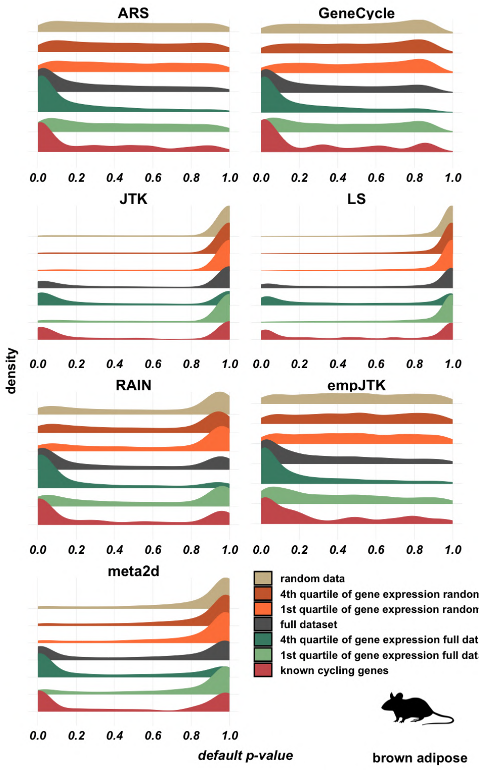

Fig. S16

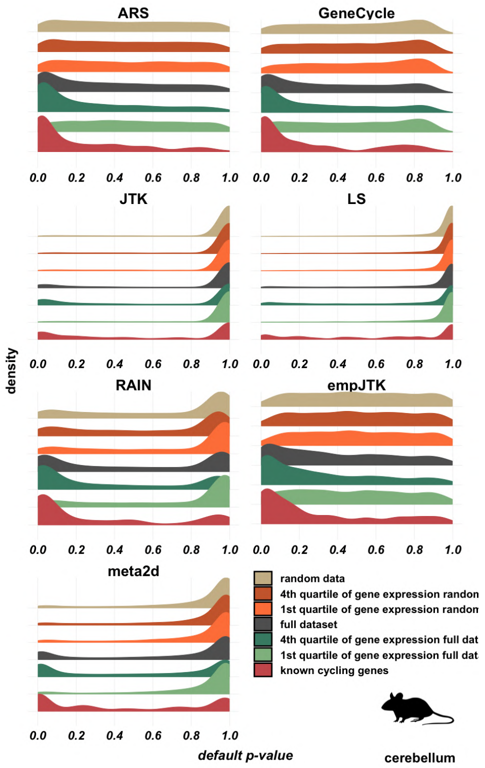

Fig. S17

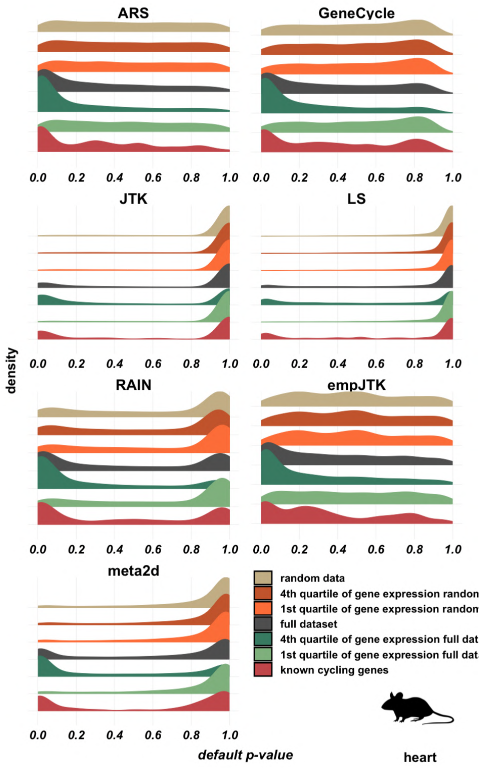

Fig. S18

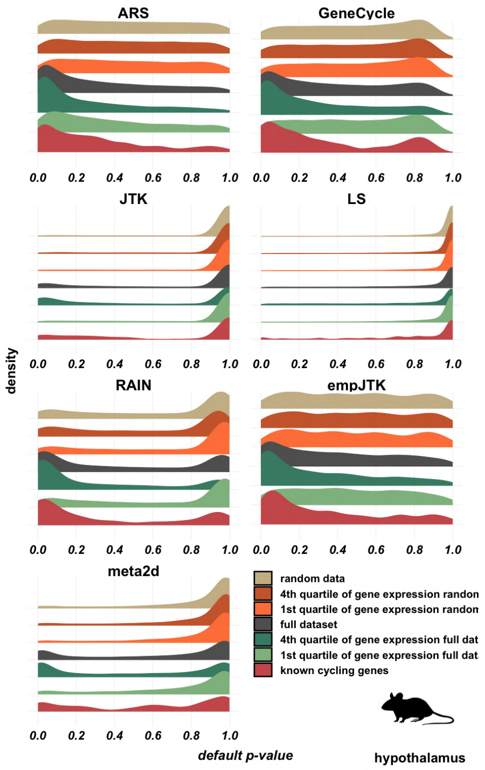

Fig. S19

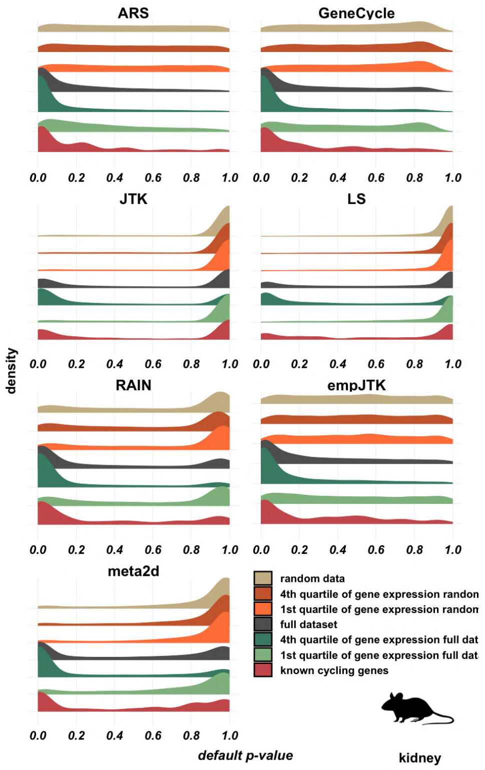

Fig. S20

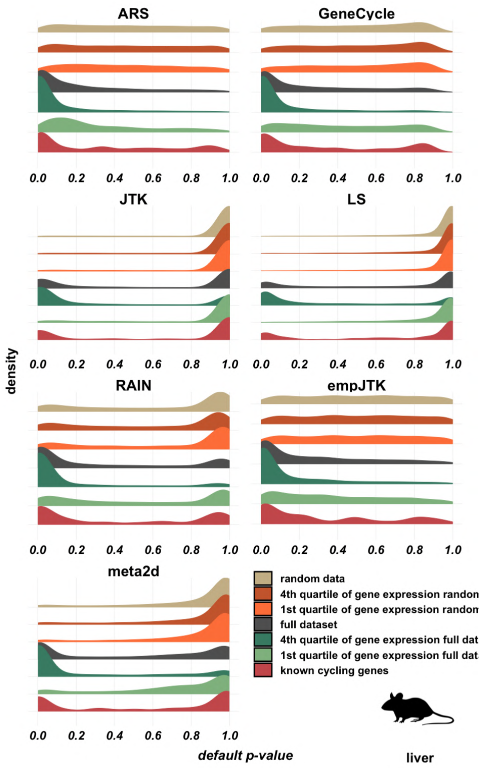

Fig. S21

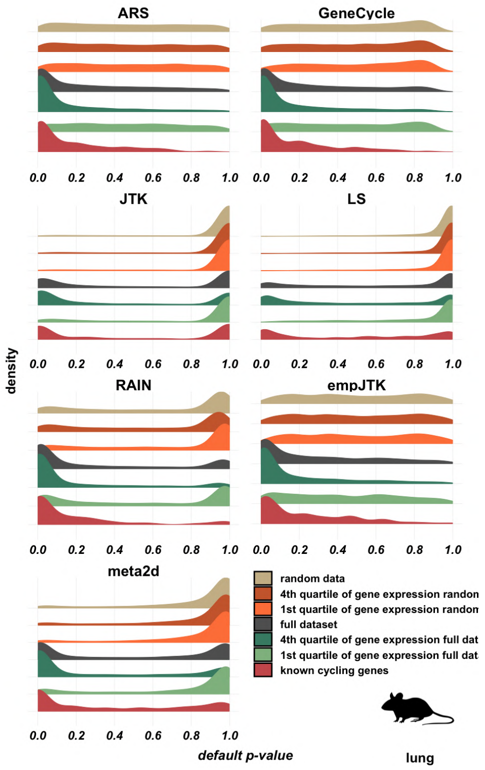

Fig. S22

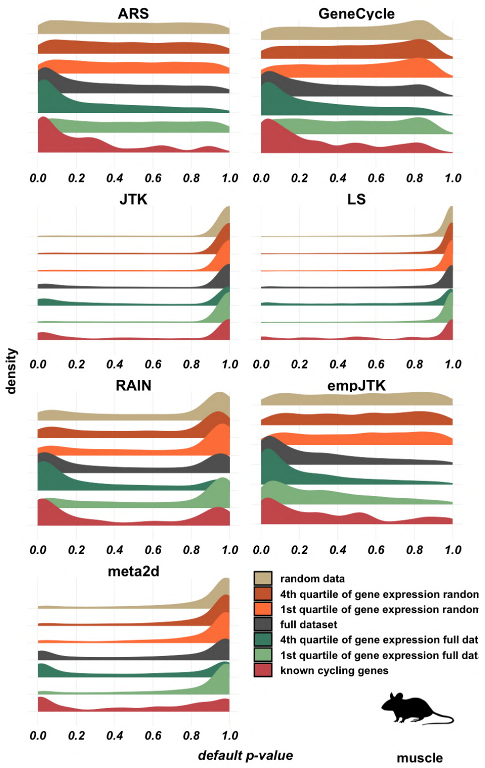

Fig. S23

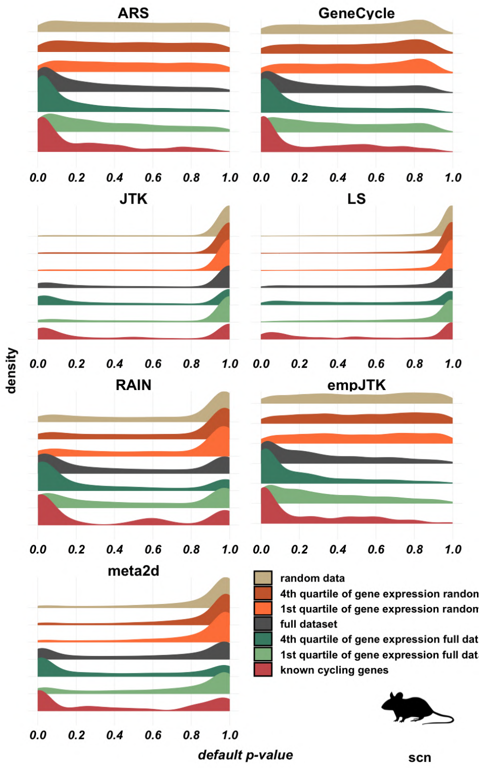

Fig. S24

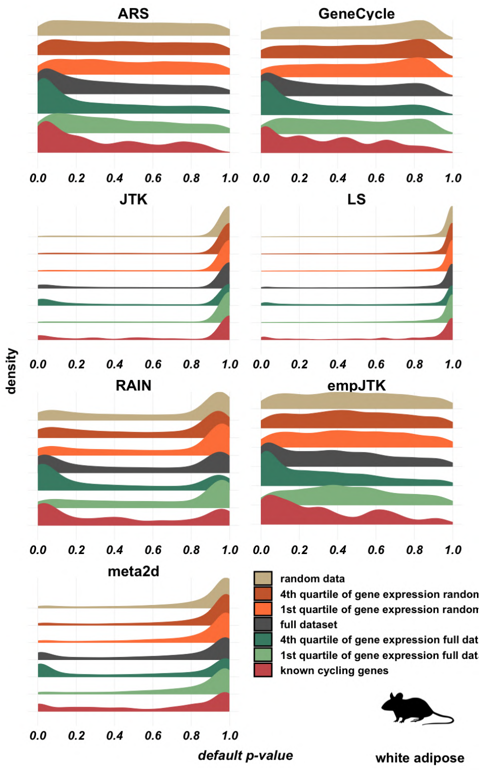

Fig. S25

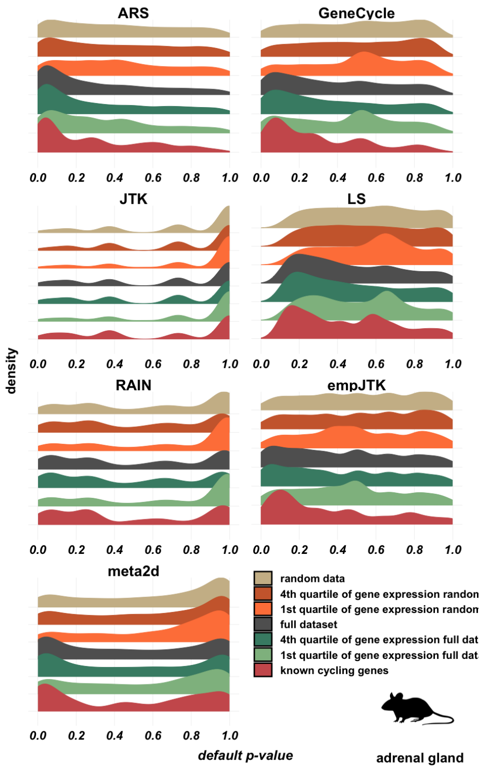

Fig. S26

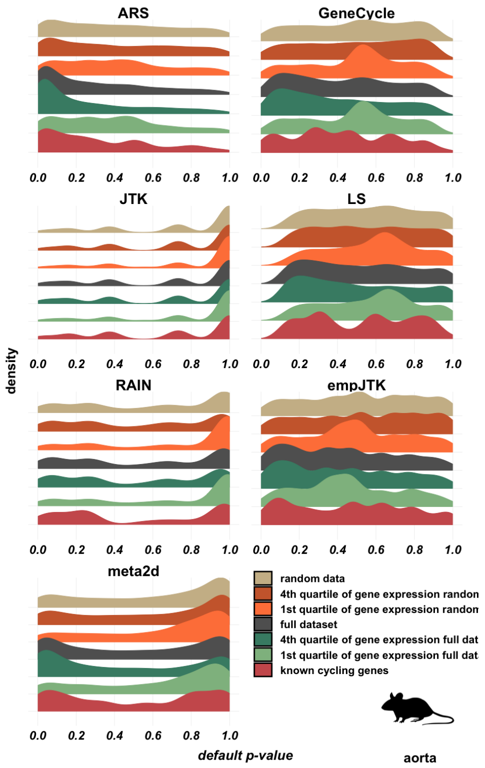

Fig. S27

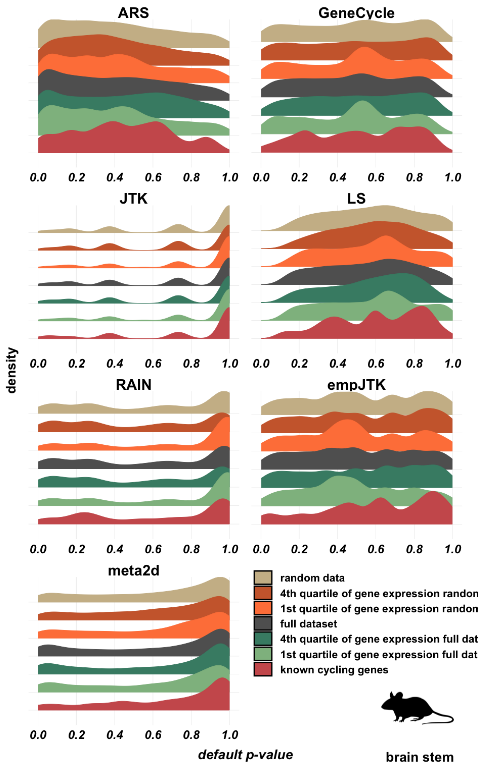

Fig. S28

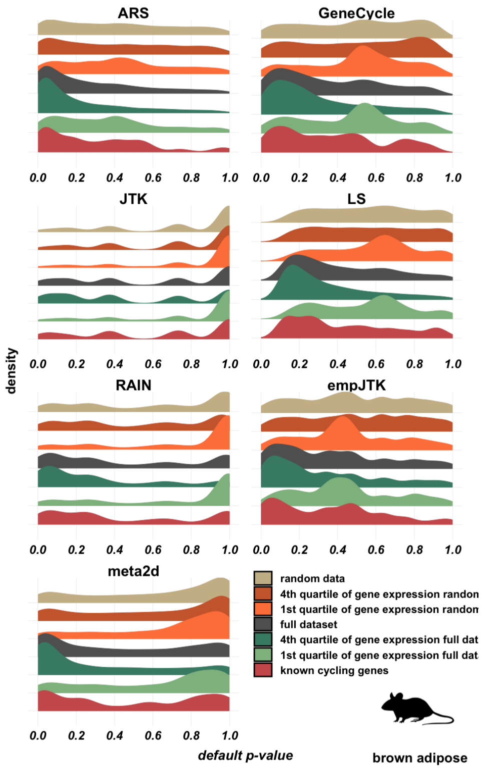

Fig. S29

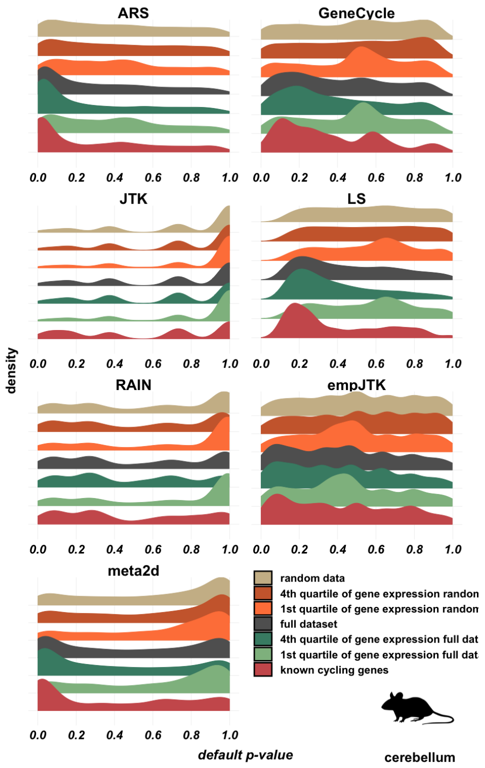

Fig. S30

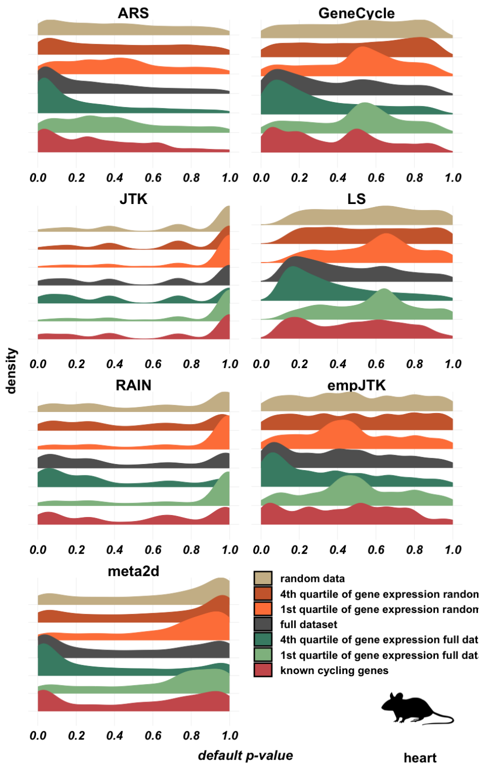

Fig. S31

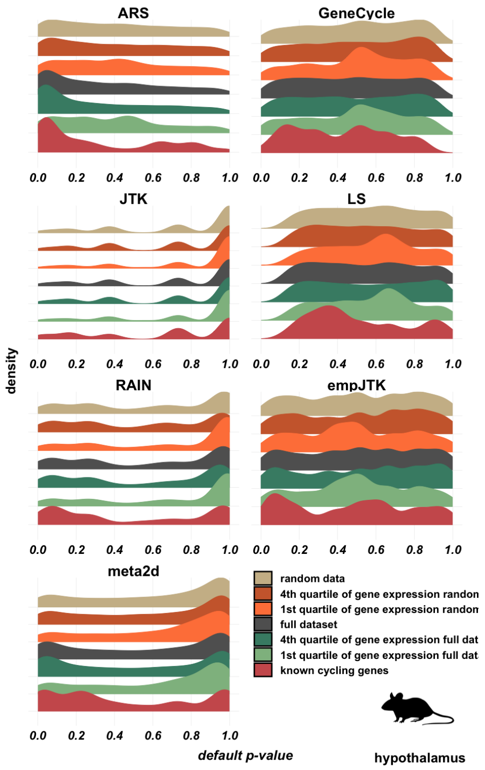

Fig. S32

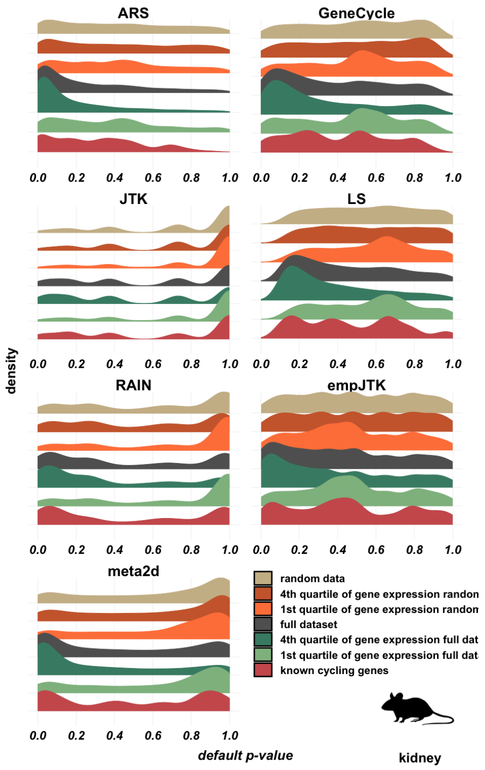

Fig. S33

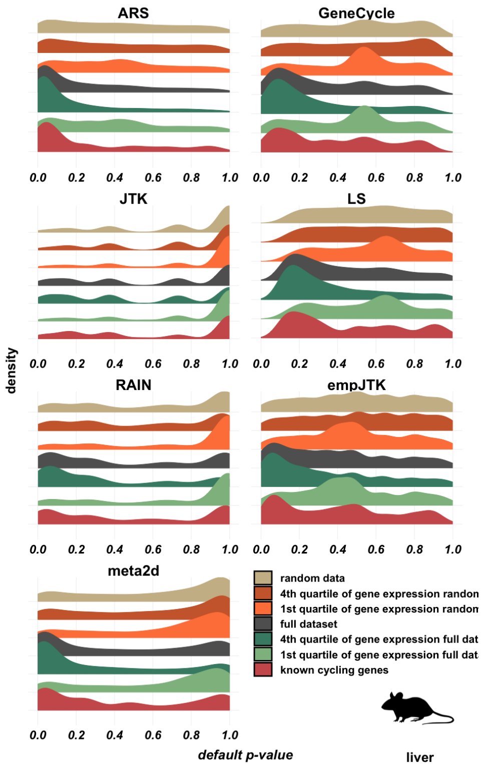

Fig. S34

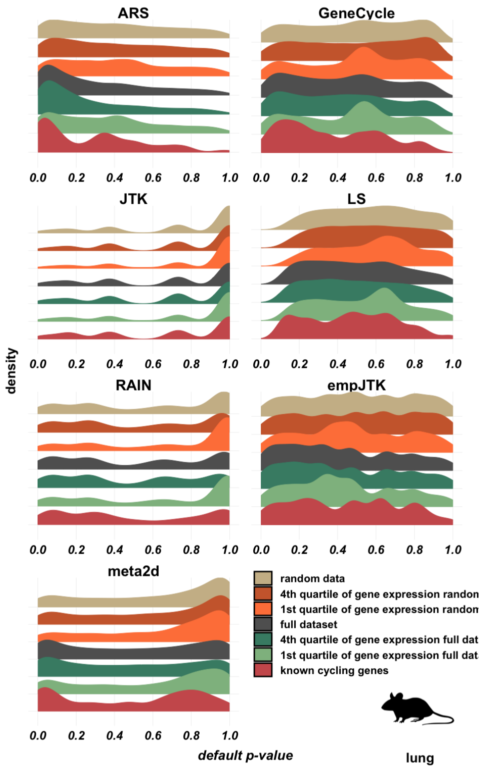

Fig. S35

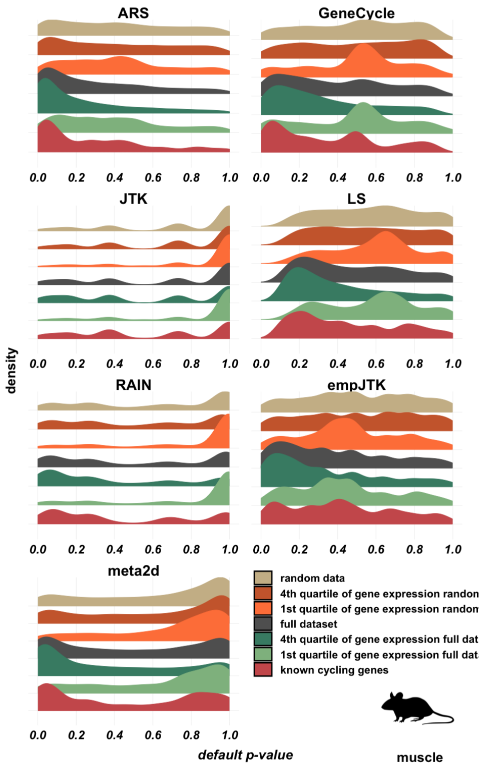

Fig. S36

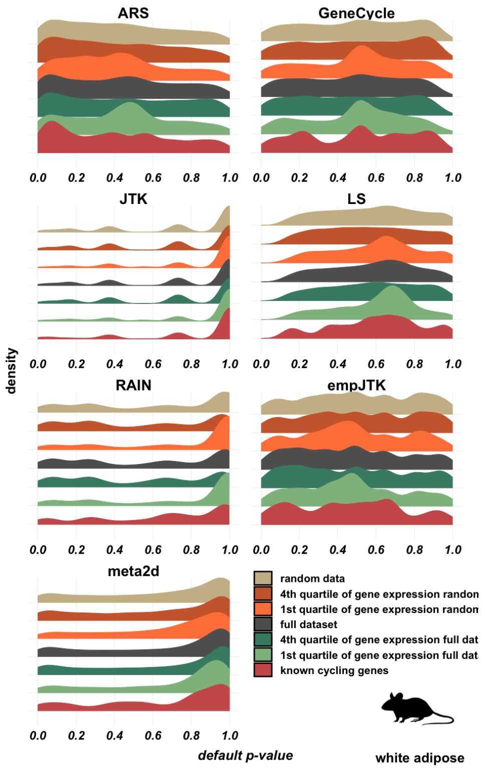

Fig. S37

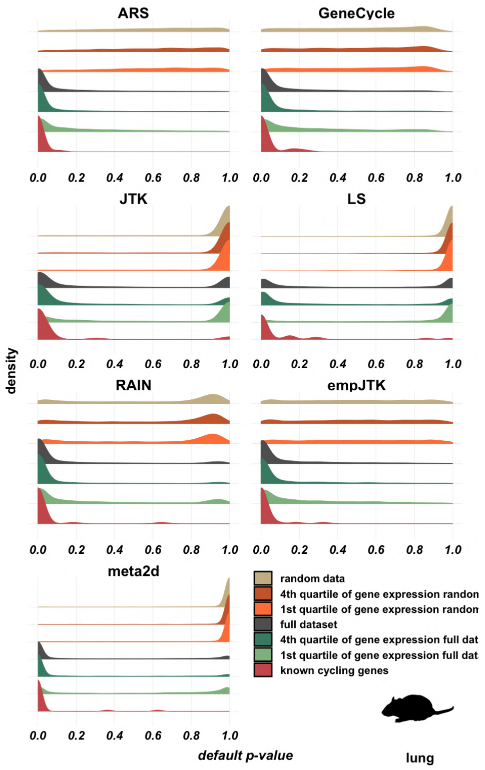

Fig. S38

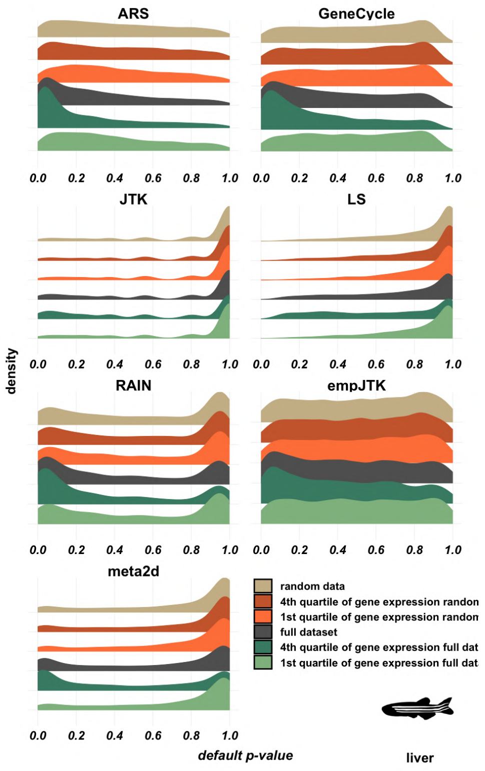

Fig. S39

raw *p*-values

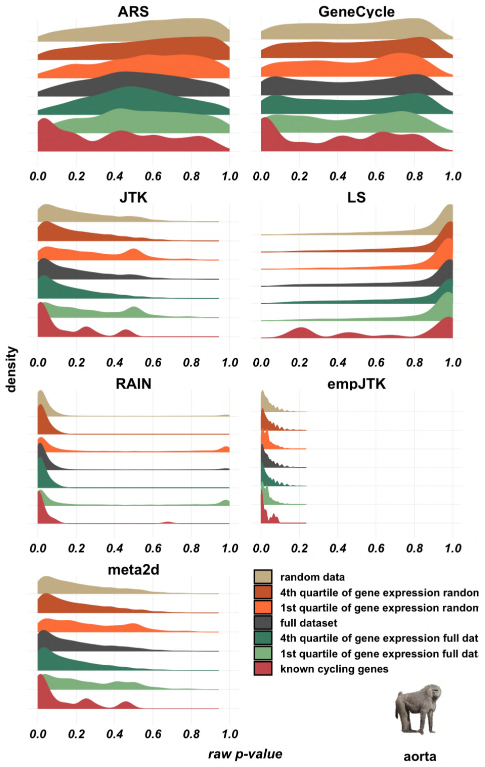

Fig. S40

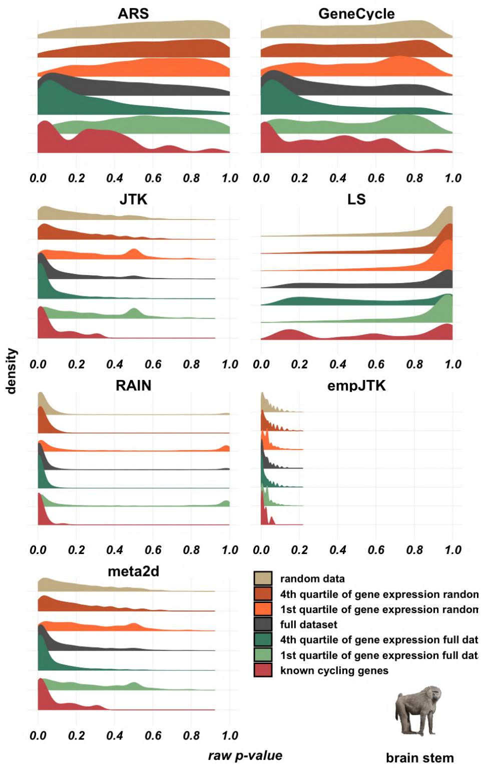

Fig. S41

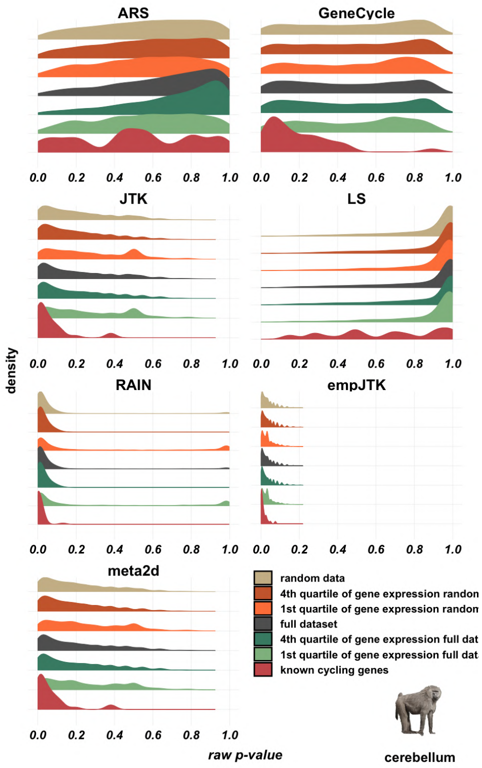

Fig. S42

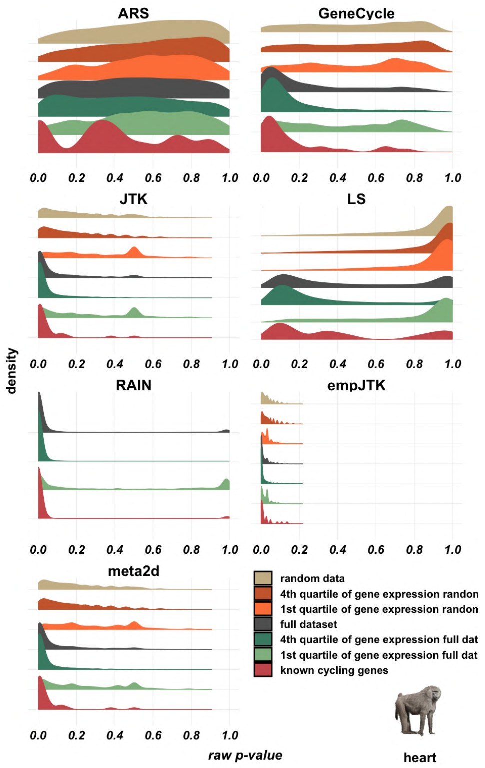

Fig. S43

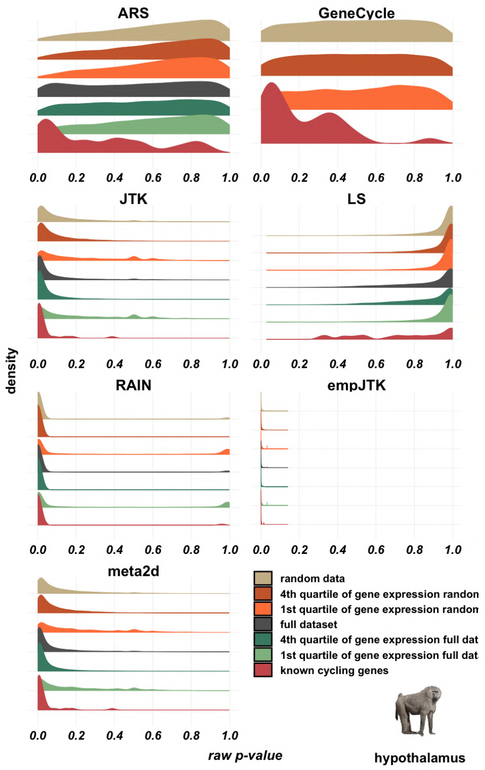

Fig. S44

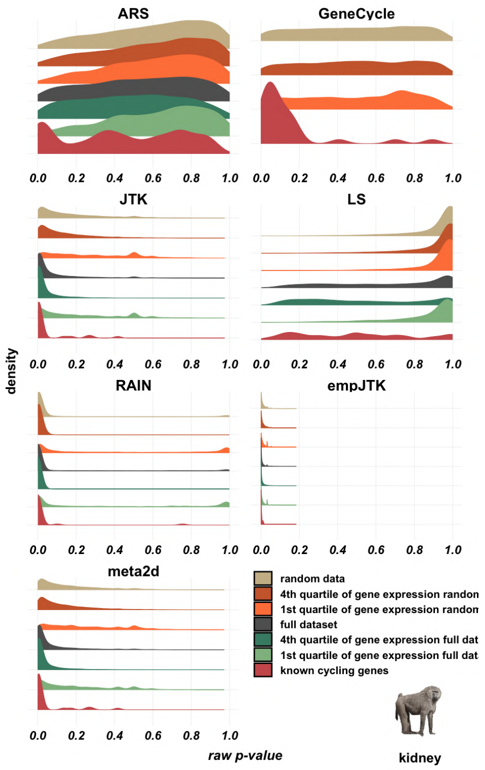

Fig. S45

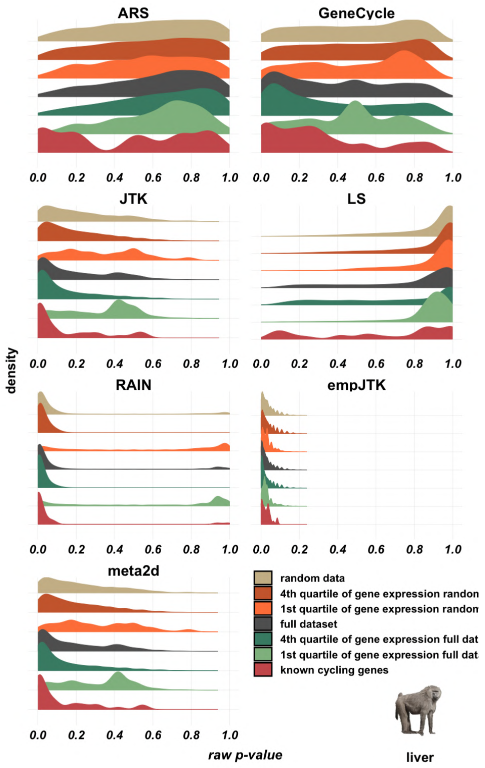

Fig. S46

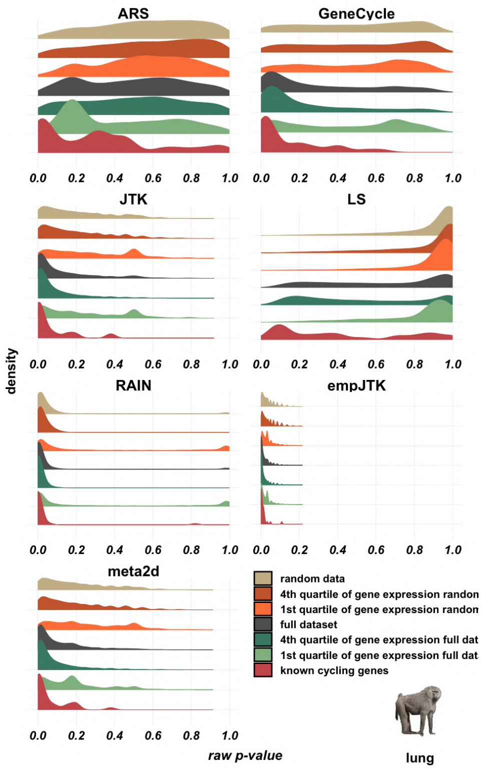

Fig. S47

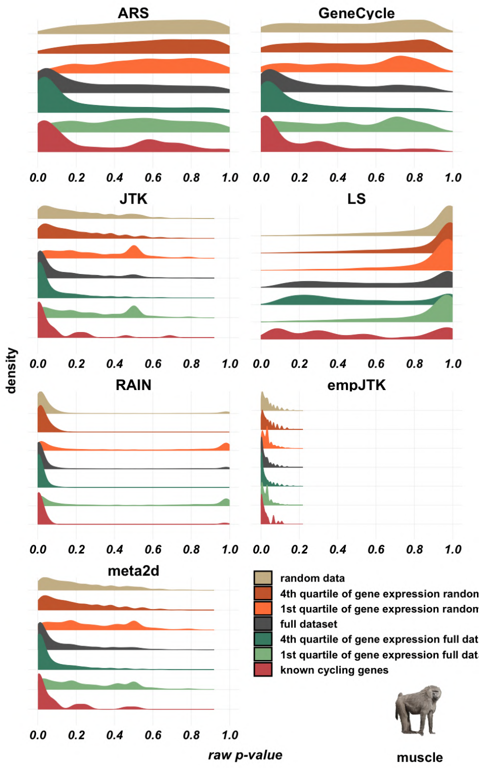

Fig. S48

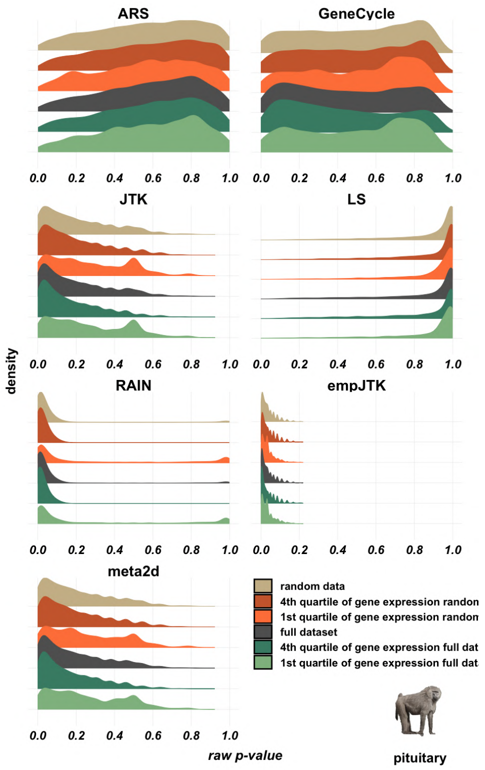

Fig. S49

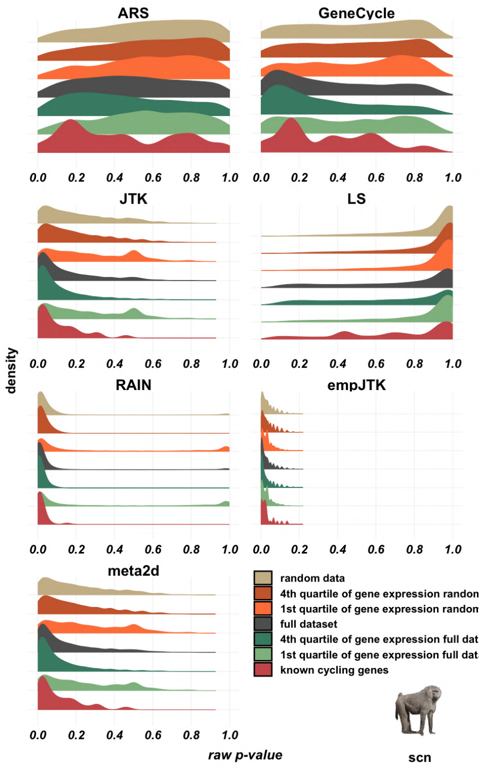

Fig. S50

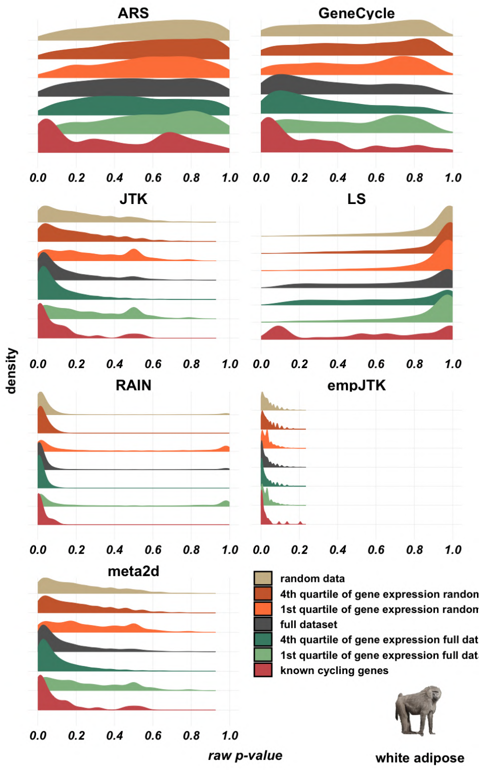

Fig. S51

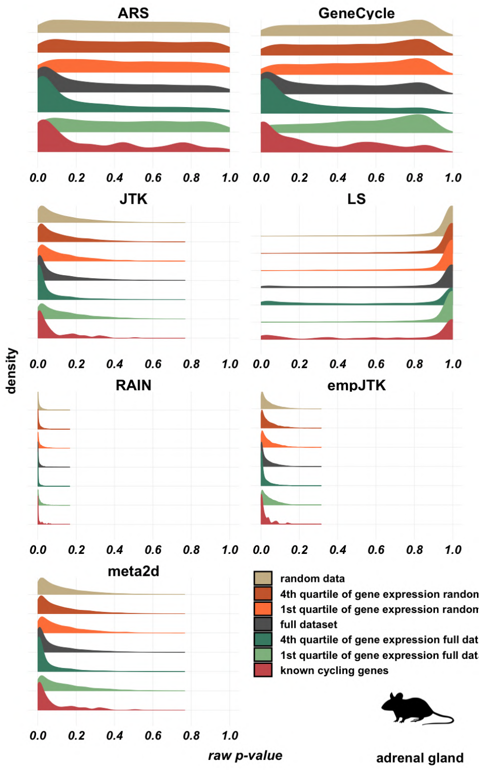

Fig. S52

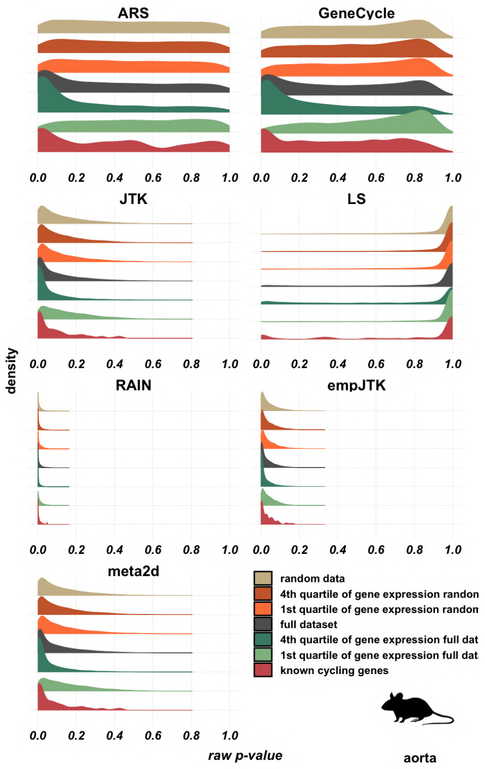

Fig. S53

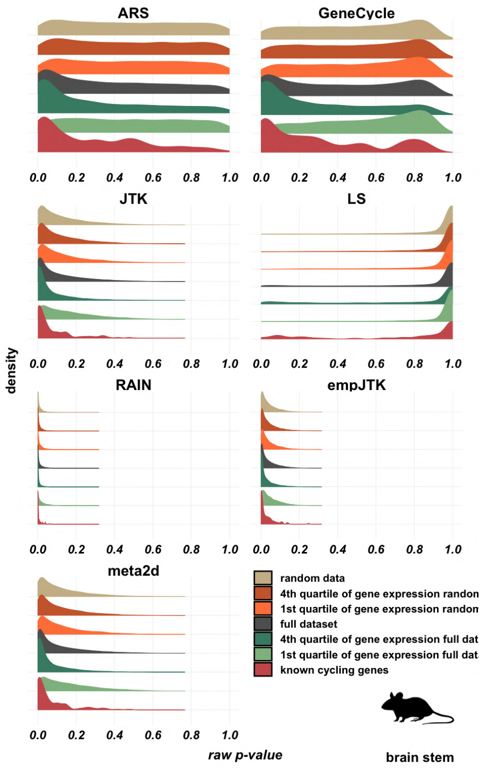

Fig. S54

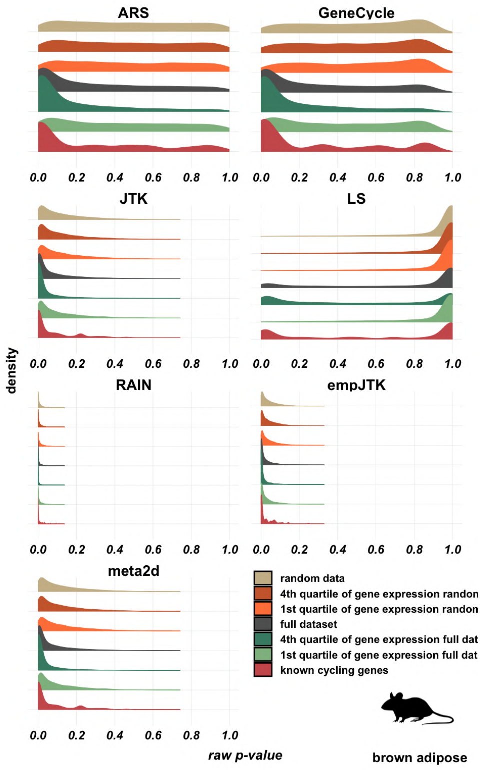

Fig. S55

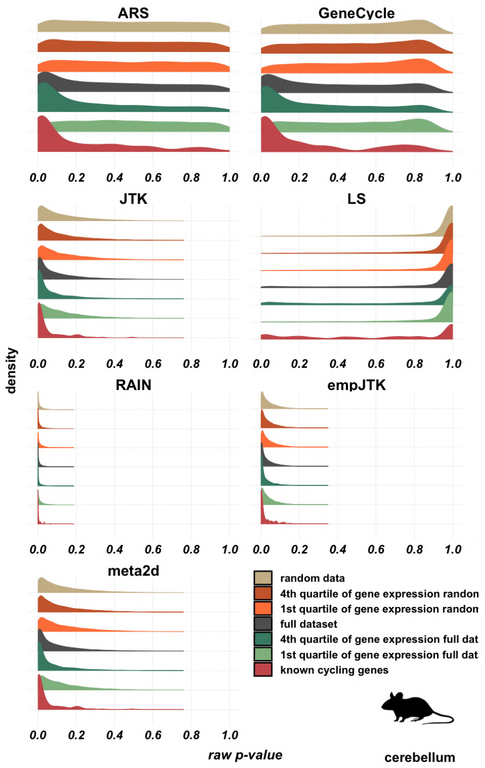

Fig. S56

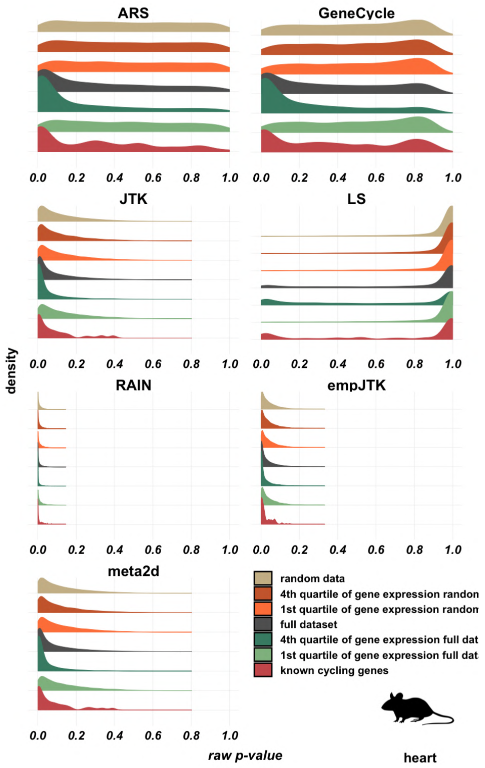

Fig. S57

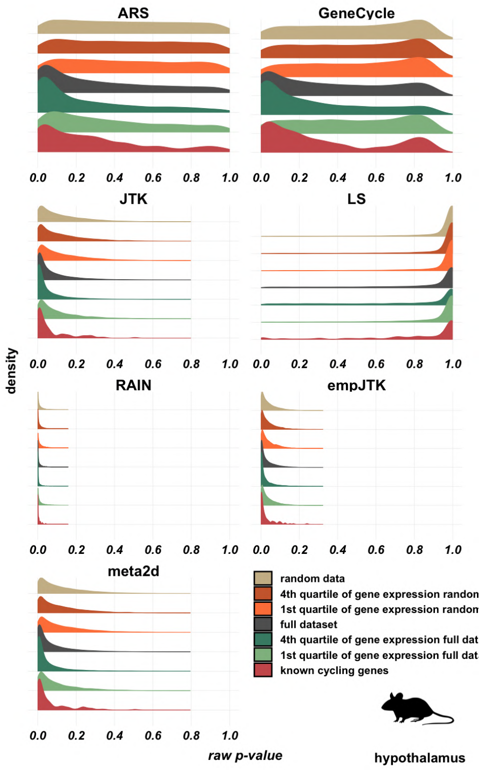

Fig. S58

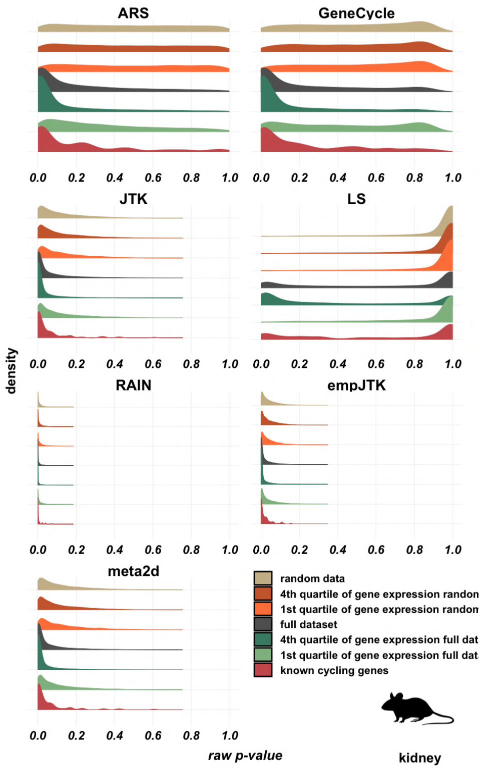

Fig. S59

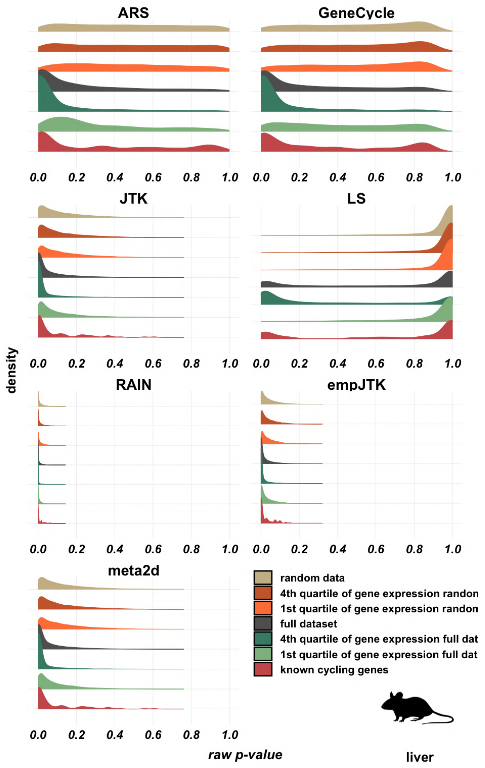

Fig. S60

Images credit: Anthony Caravaggi (mouse), Ian Quigley (zebrafish) both license CC BY-NC-SA 3.0, Wikipedia GNU GPL Muhammad Mahdi Karim (baboon), and Public Domain for other images (from http://phylopic.org/)
