# Supplementary material for: Methods detecting rhythmic gene expression are biologically relevant only for strong signal
Source: PLoS Comput Biol. 2020 Mar 17;16(3):e1007666. doi: 10.1371/journal.pcbi.1007666 (PMC7100990; doi:10.1371/journal.pcbi.1007666)
Supplement: S3 File — (PDF) [file pcbi.1007666.s004.pdf]

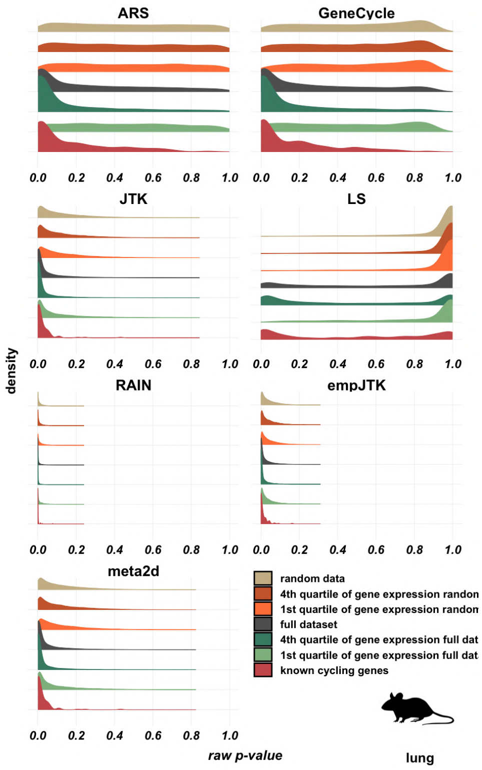

Fig. S61

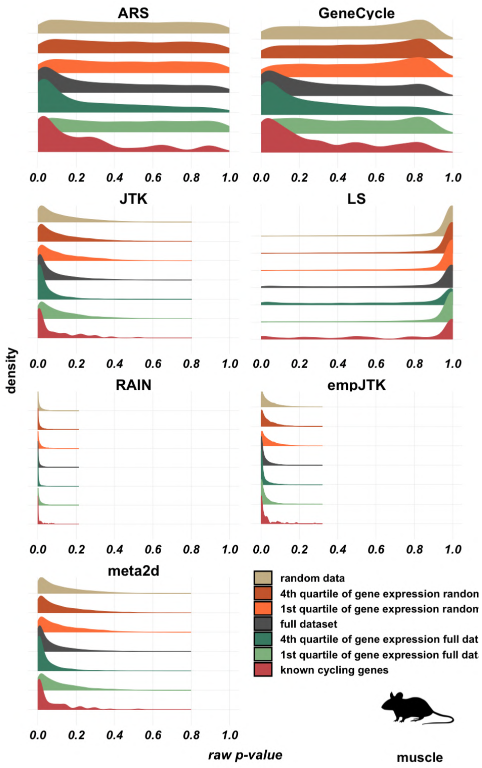

Fig. S62

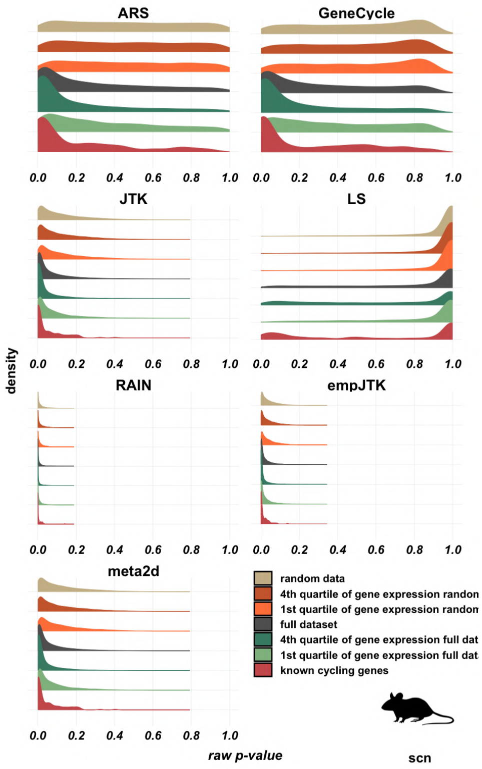

Fig. S63

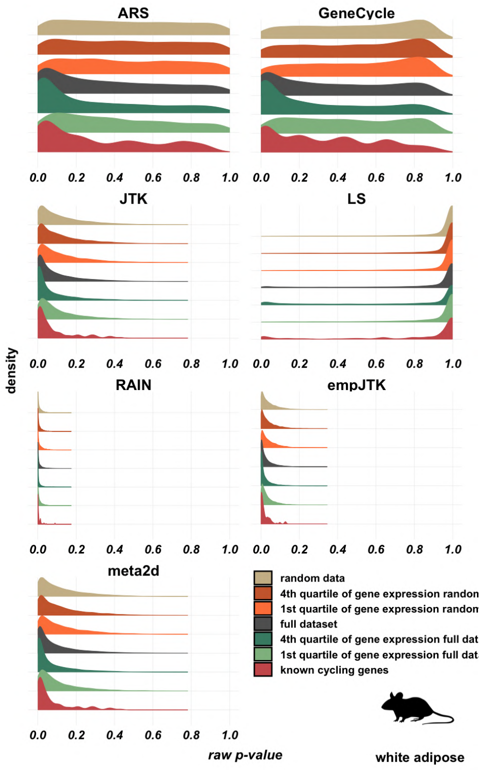

Fig. S64

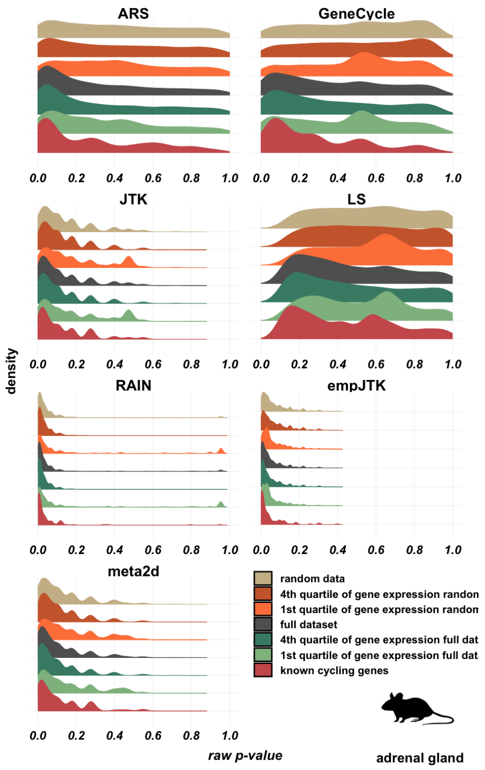

Fig. S65

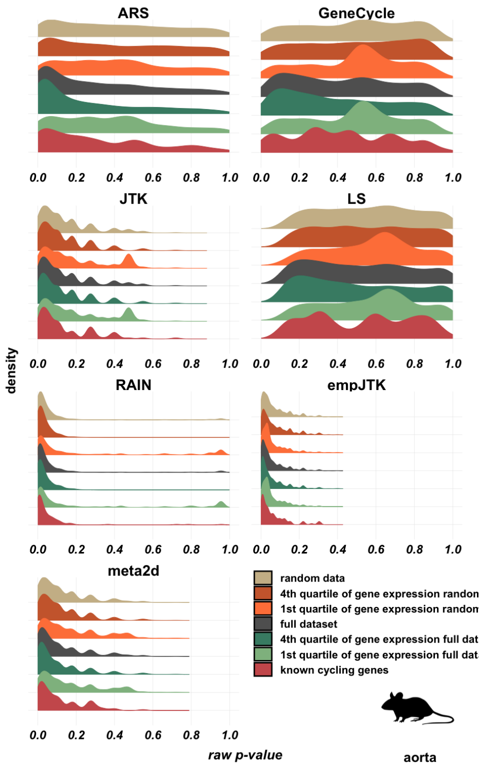

Fig. S66

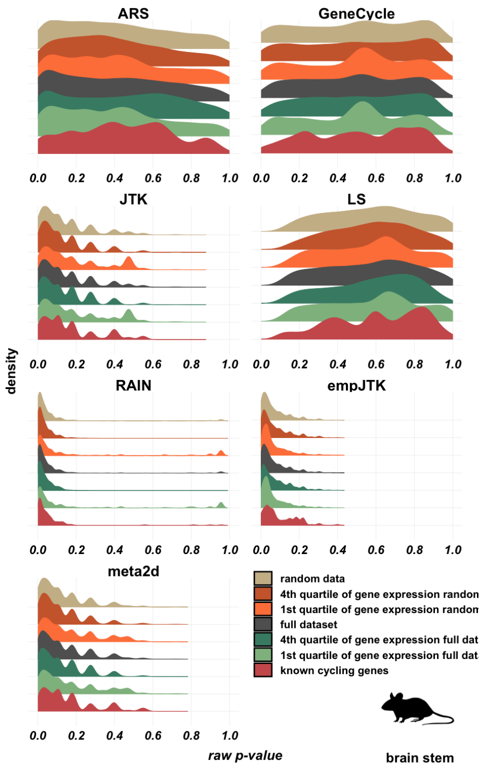

Fig. S67

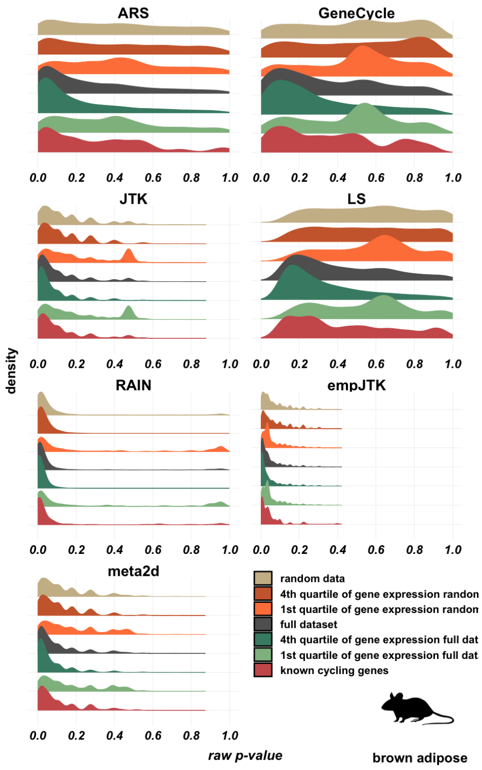

Fig. S68

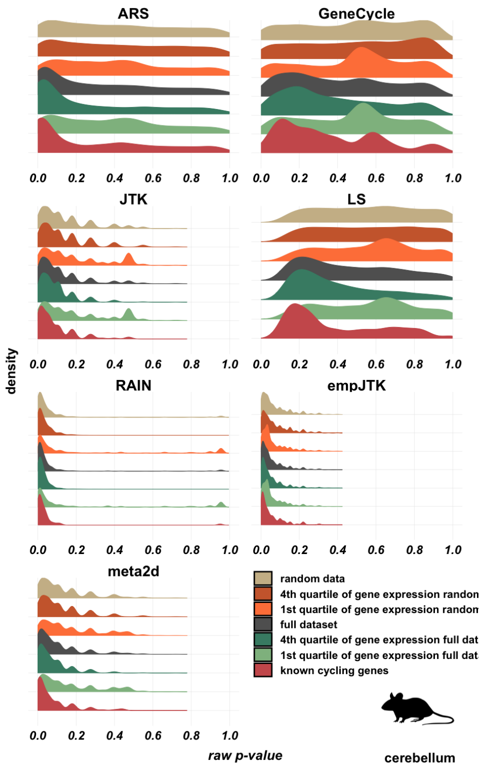

Fig. S69

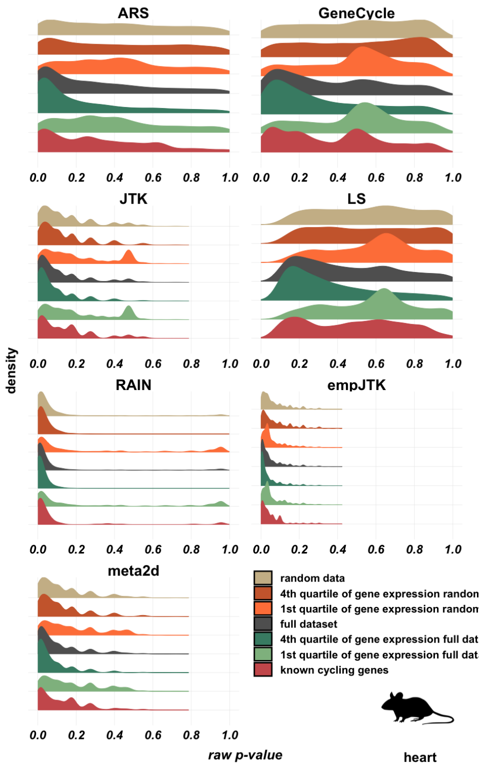

Fig. S70

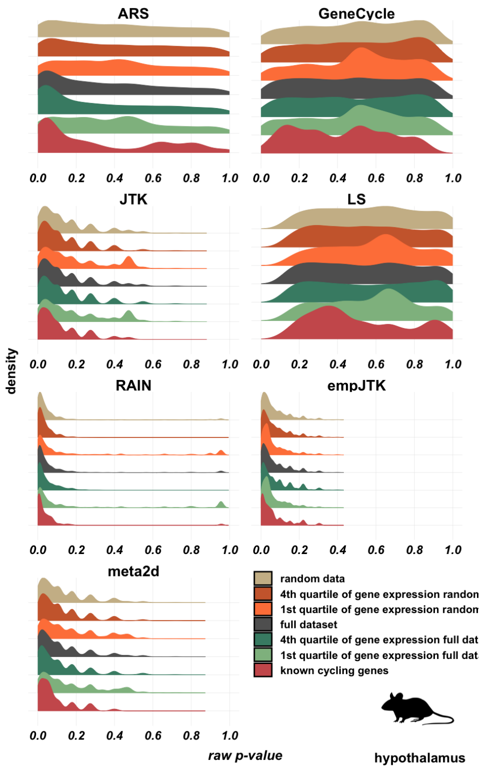

Fig. S71

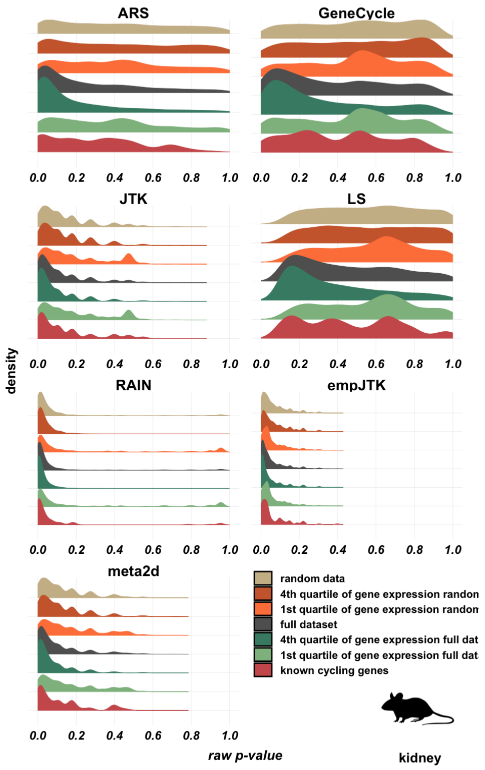

Fig. S72

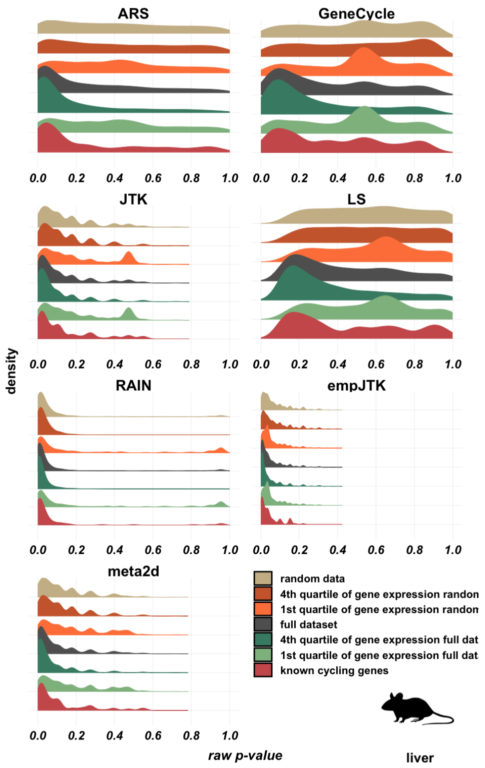

Fig. S73

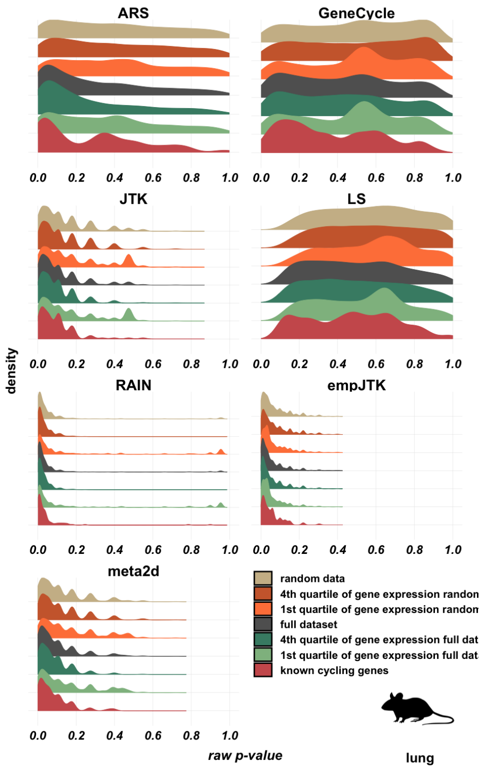

Fig. S74

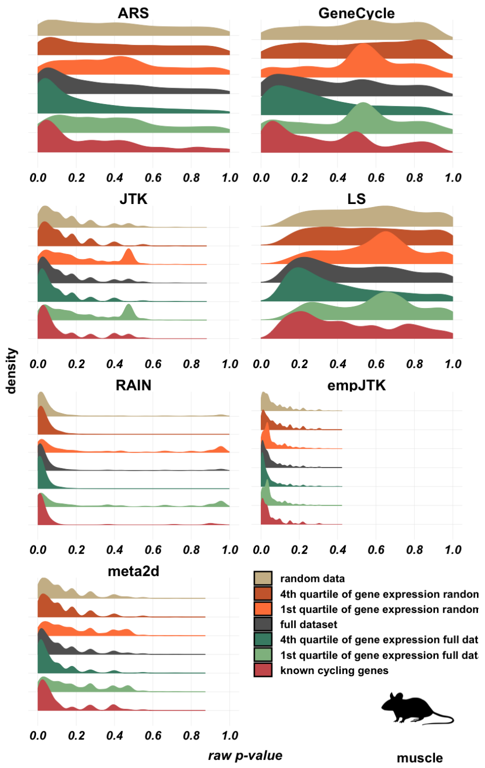

Fig. S75

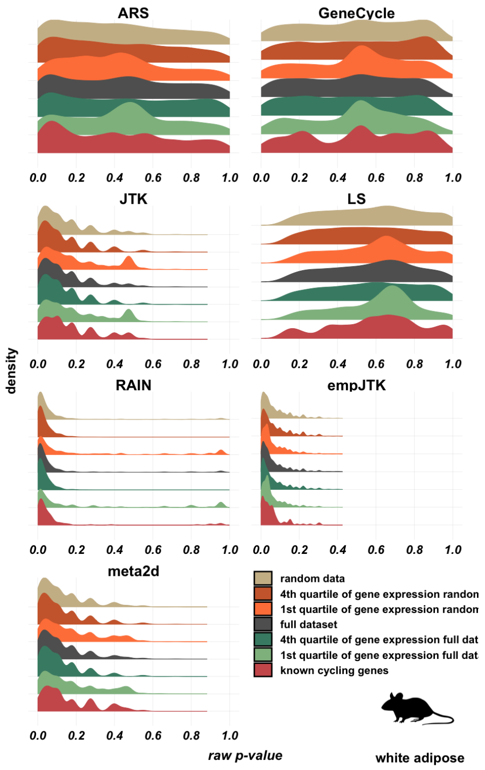

Fig. S76

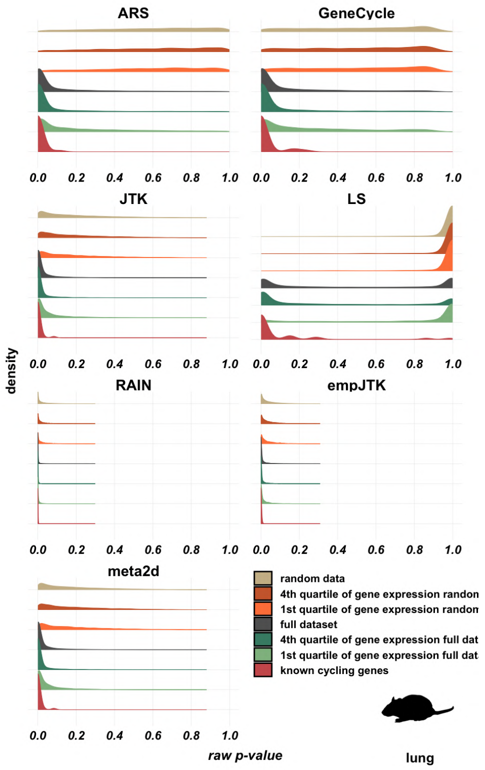

Fig. S77

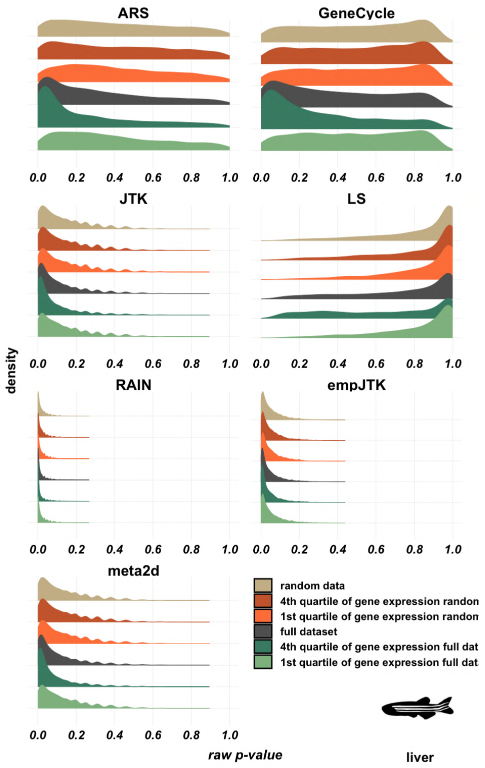

Fig. S78

BH.Q

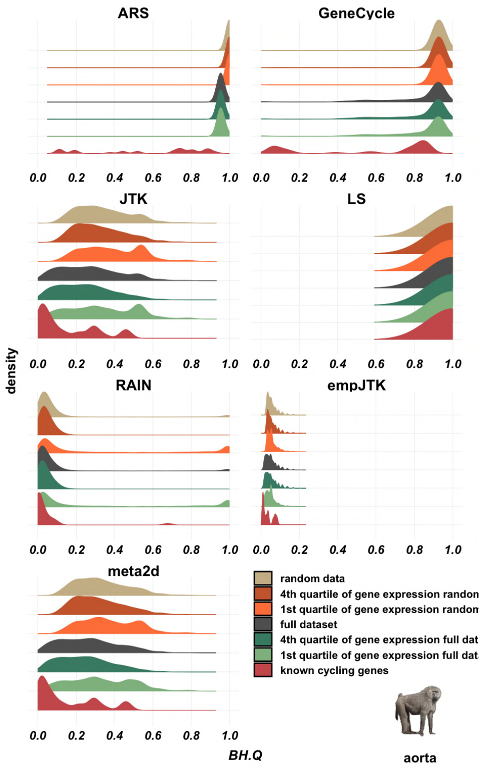

Fig. S78

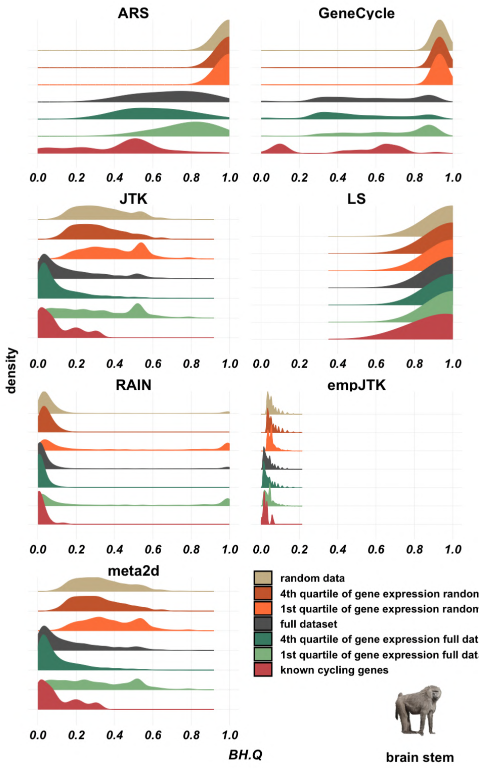

Fig. S78

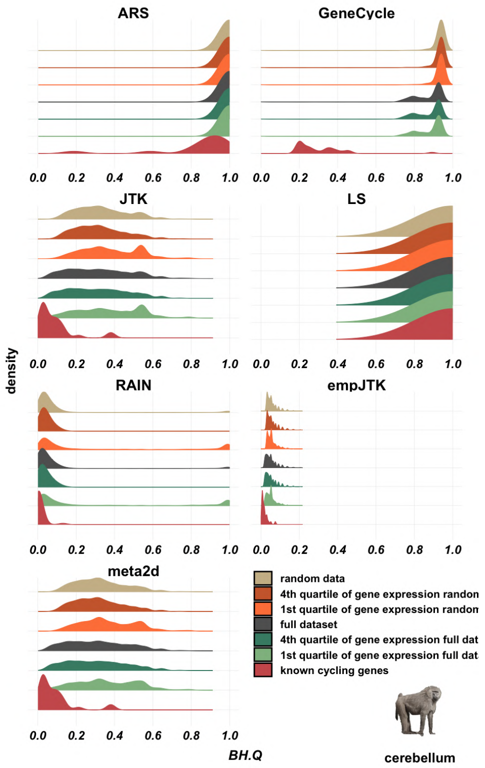

Fig. S78

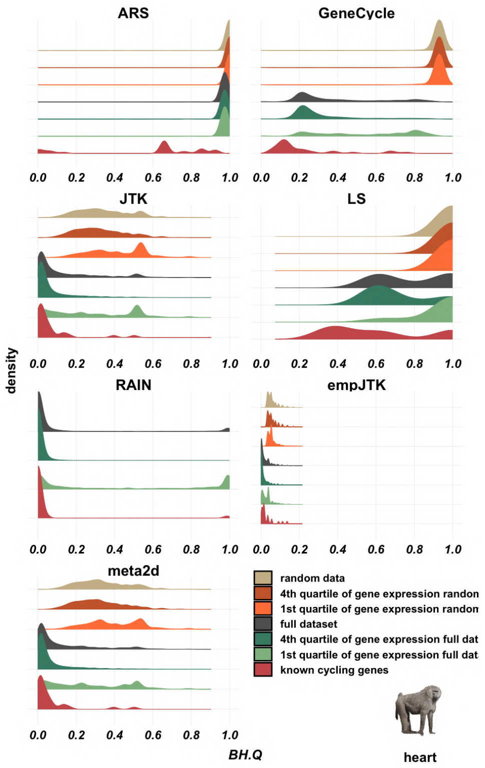

Fig. S78

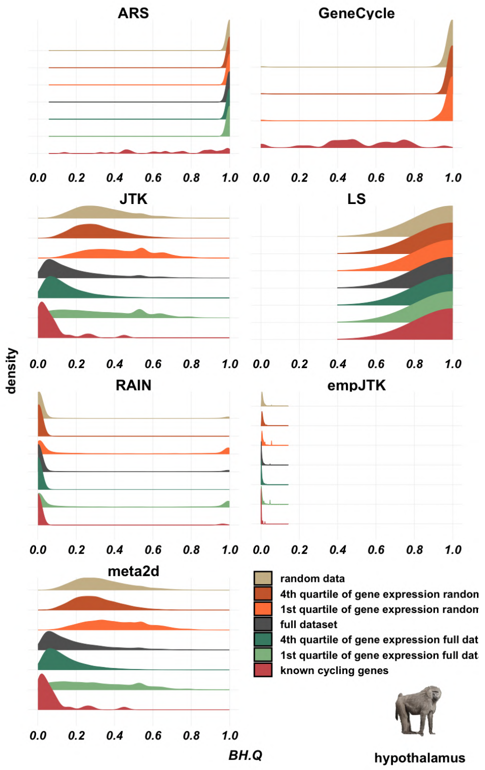

Fig. S78

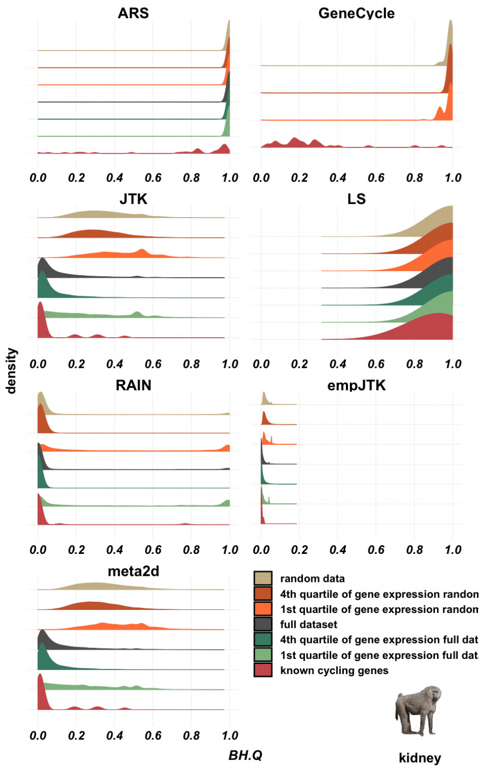

Fig. S78

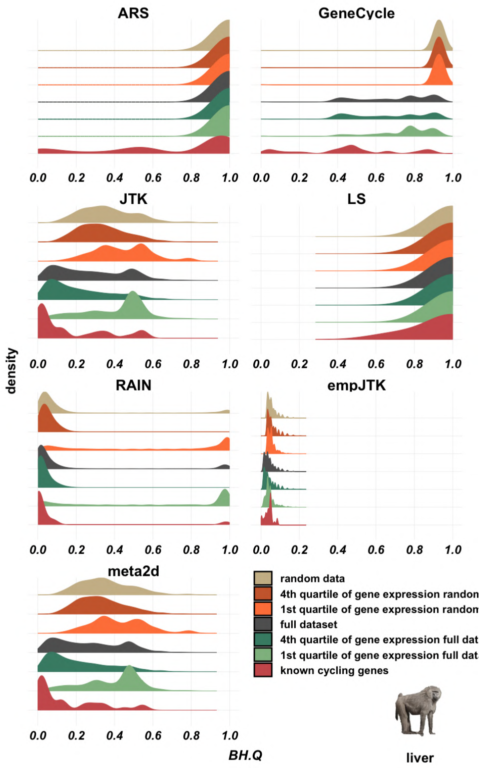

Fig. S78

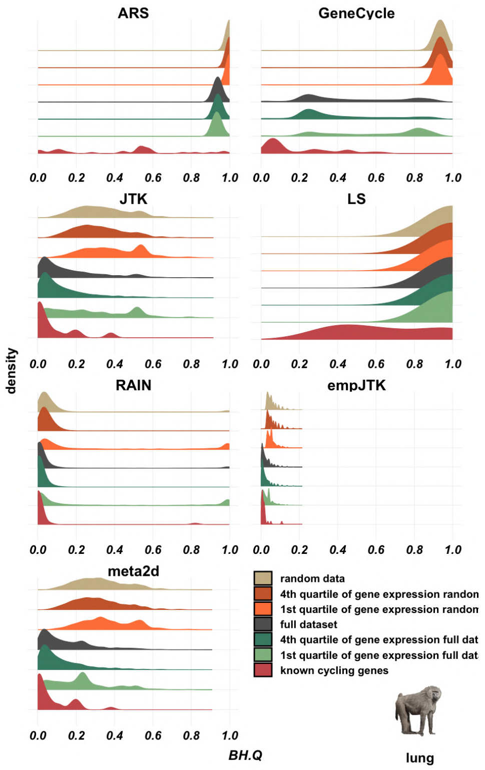

Fig. S78

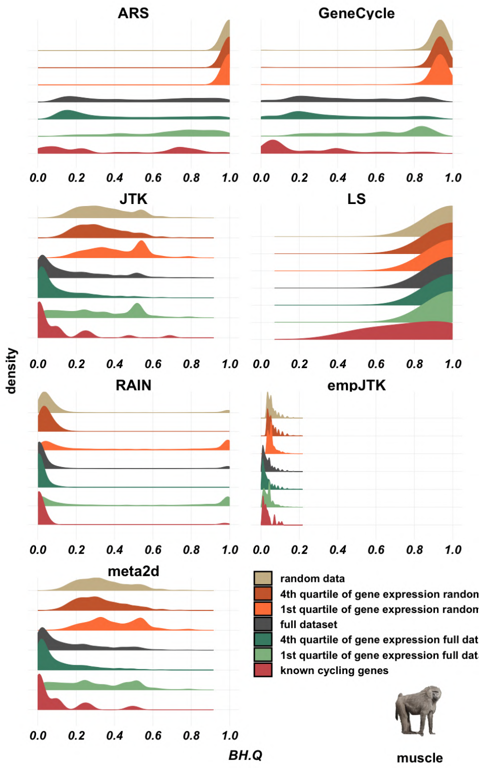

Fig. S78

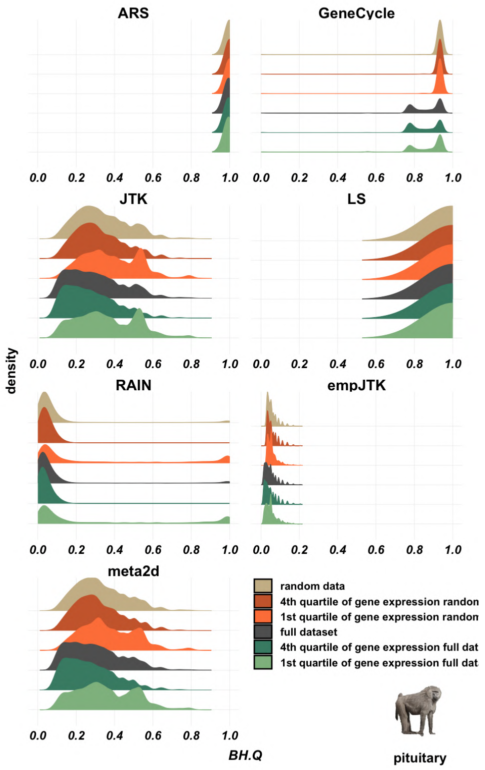

Fig. S78

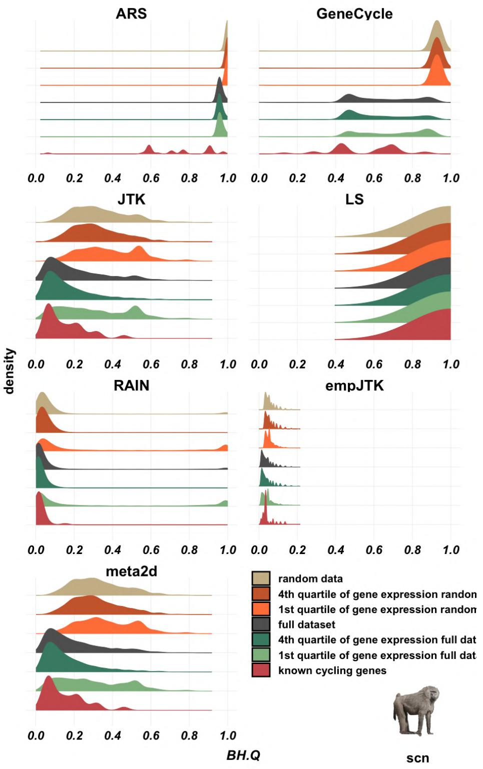

Fig. S78

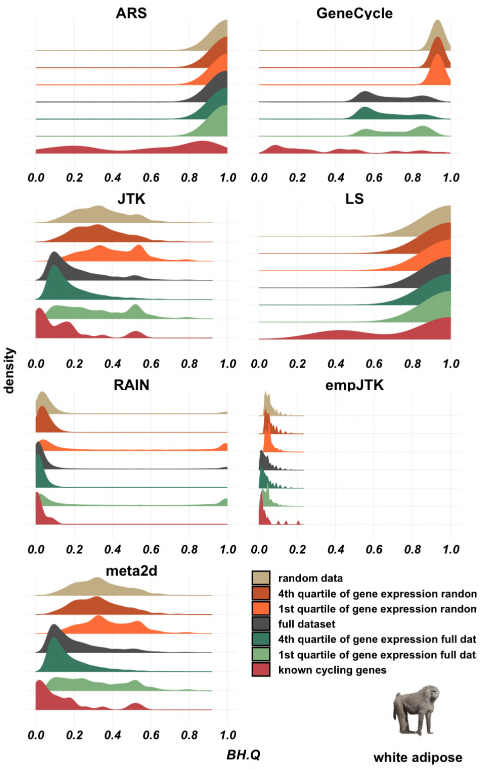

Fig. S78

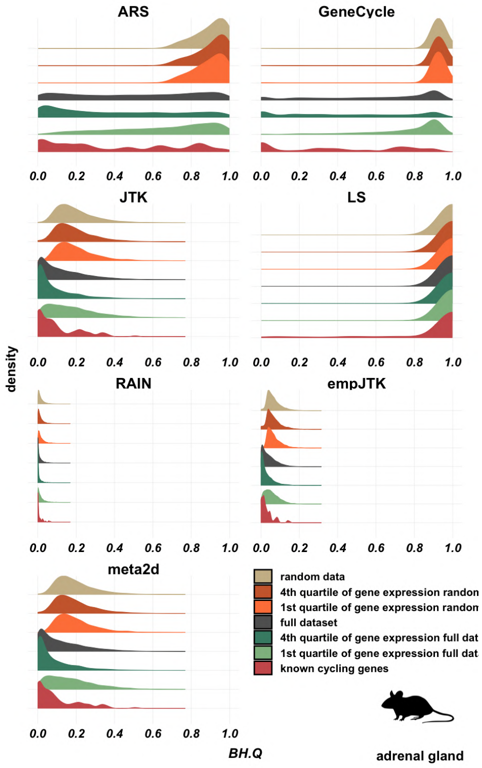

Fig. S78

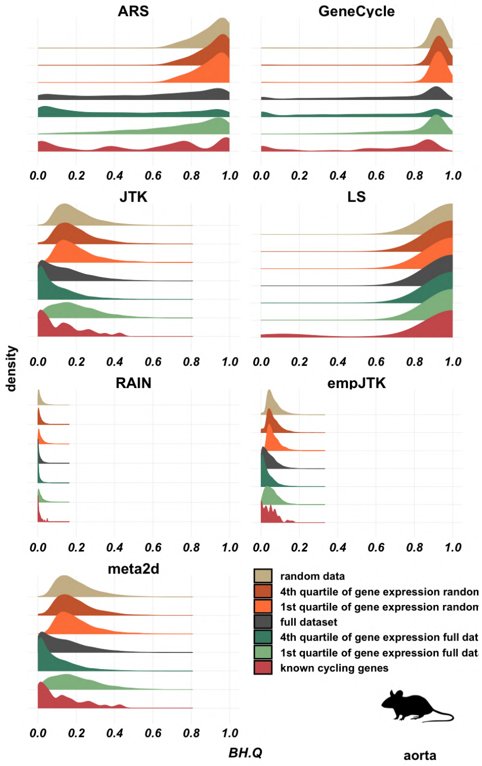

Fig. S78

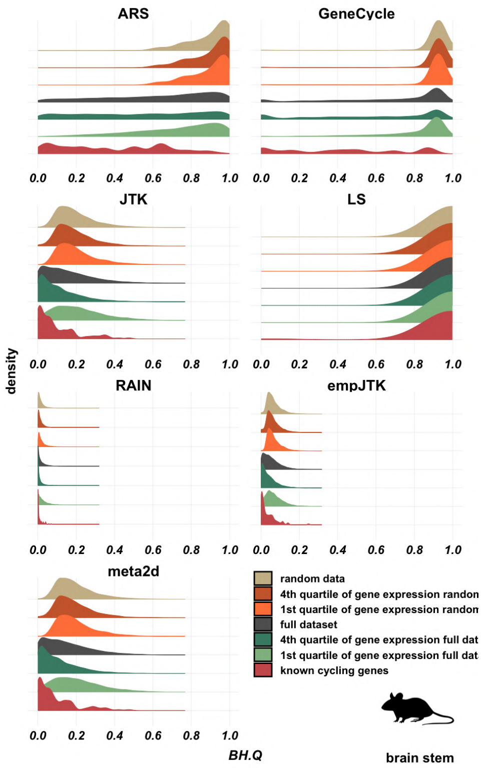

Fig. S78

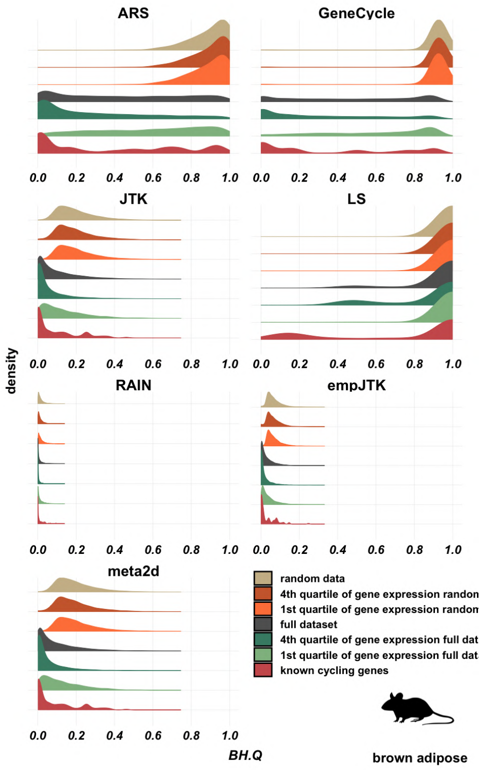

Fig. S78

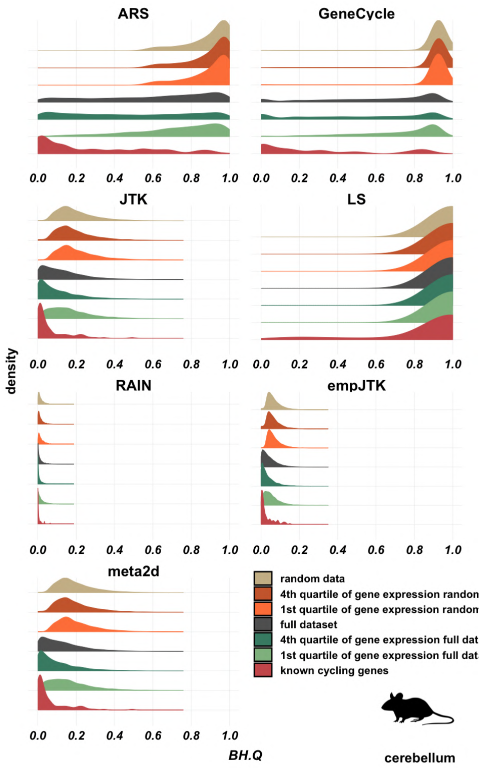

Fig. S78

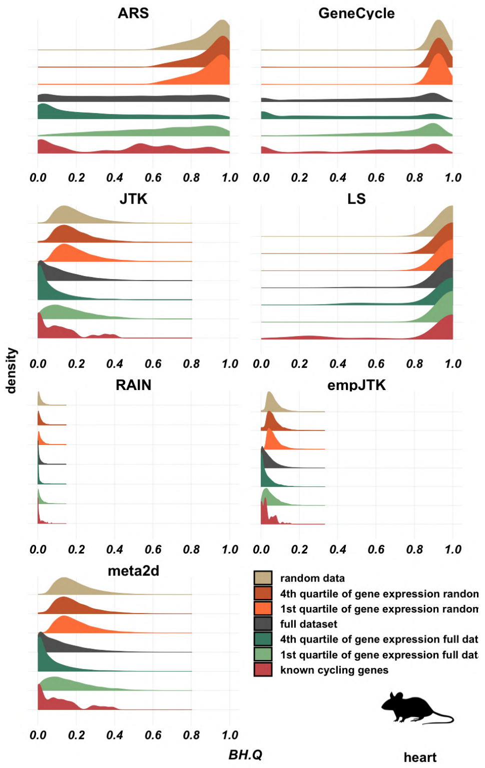

Fig. S78

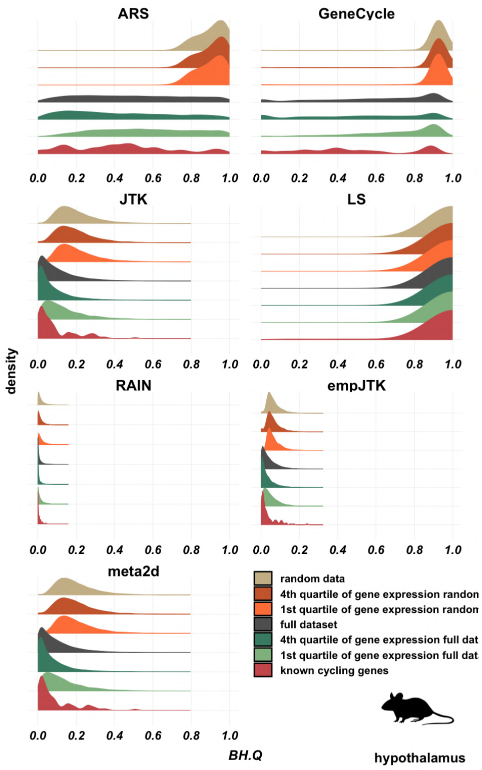

Fig. S78

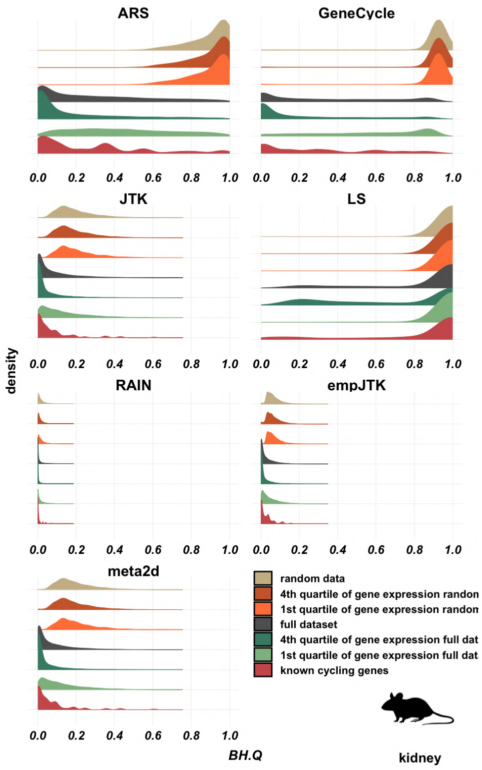

Fig. S78

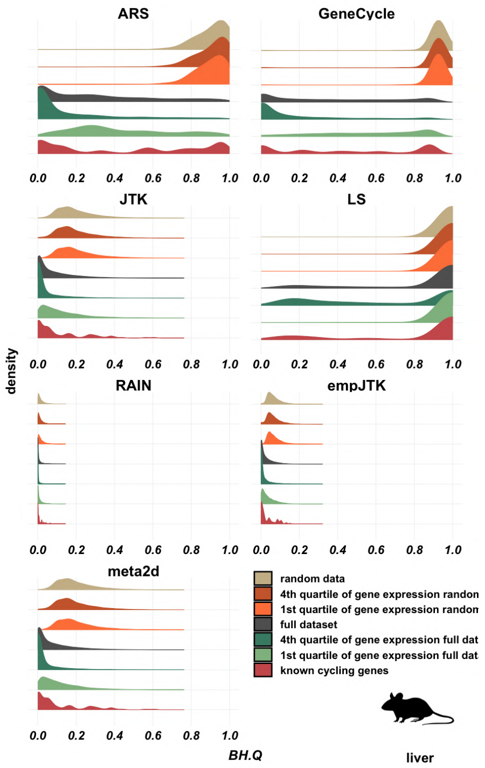

Fig. S78

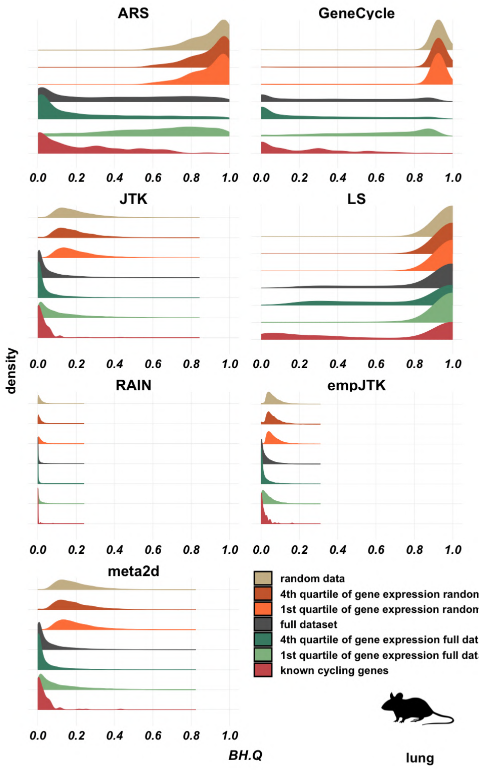

Fig. S78

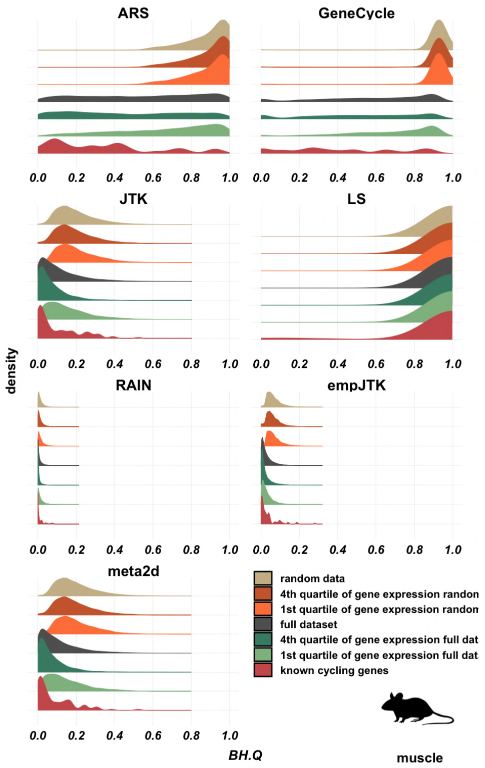

Fig. S78

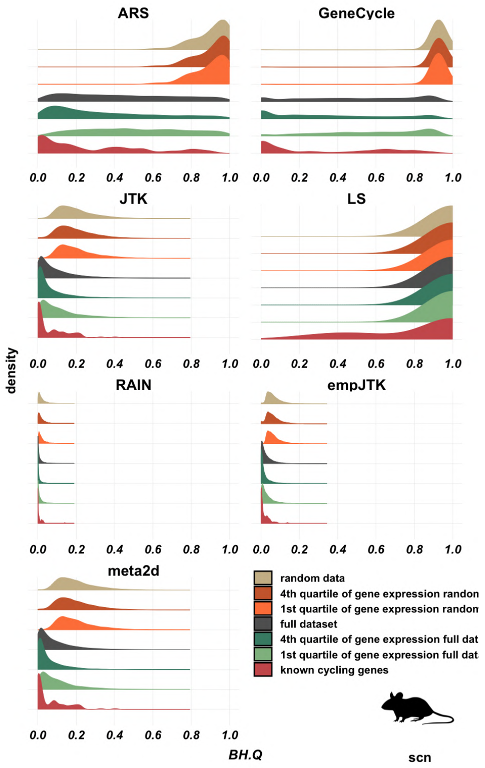

Fig. S78

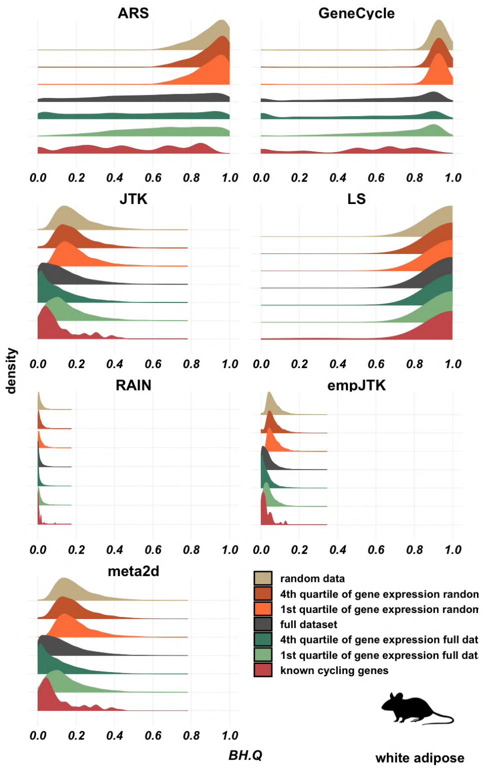

Fig. S78

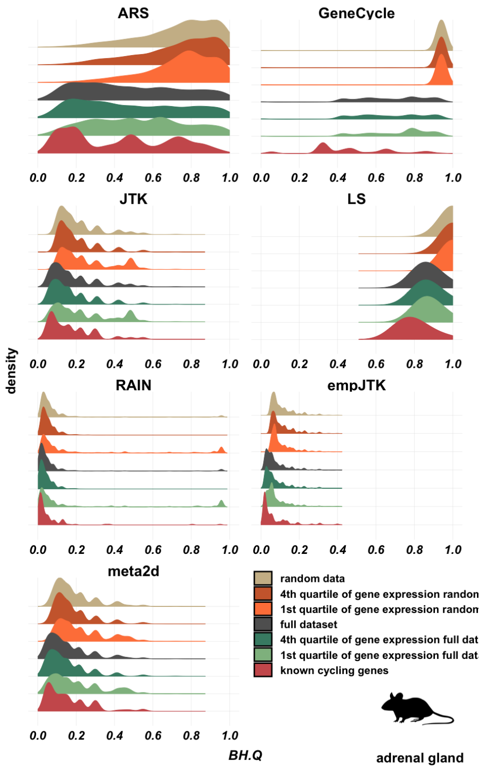

Fig. S78

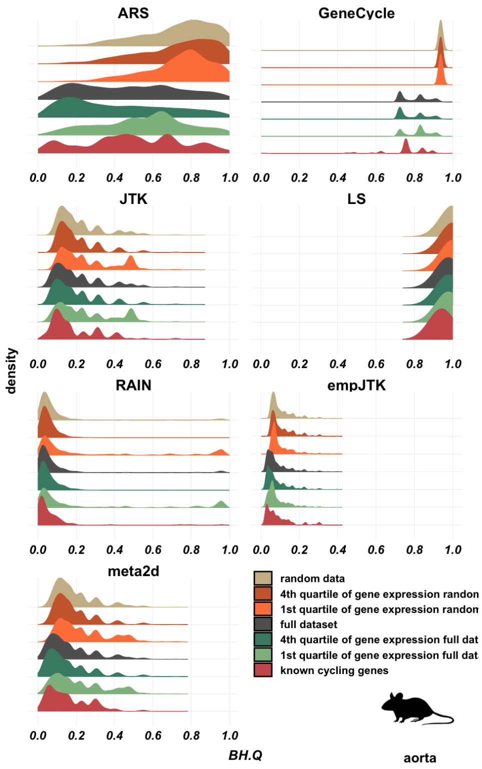

Fig. S78

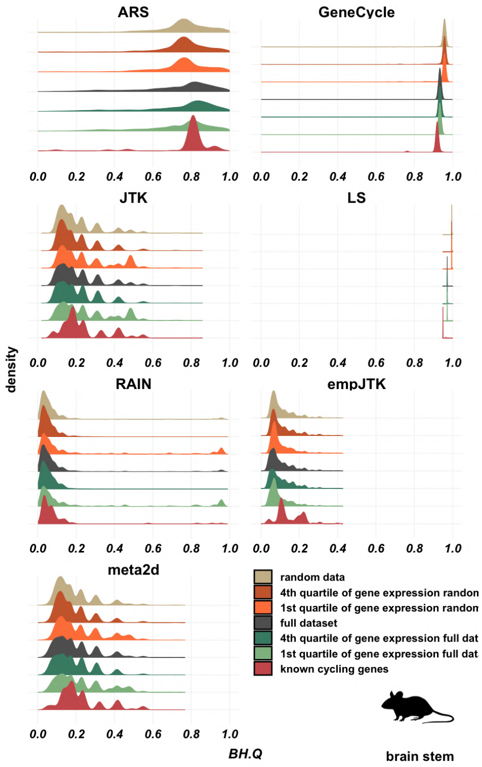

Fig. S78

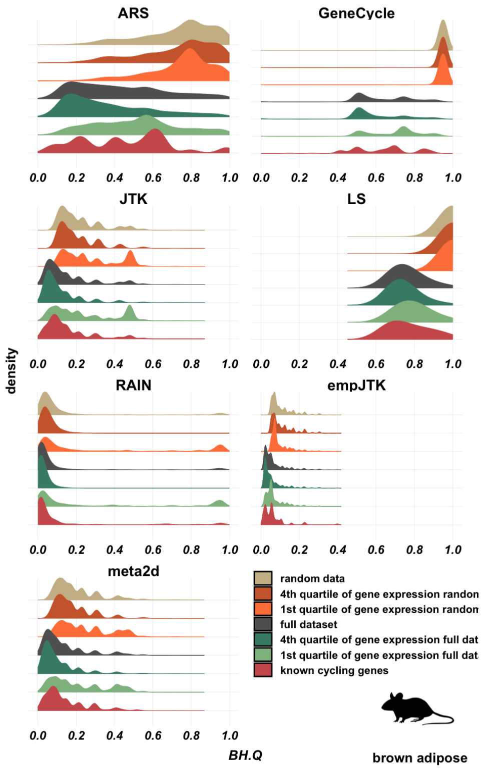

Fig. S78

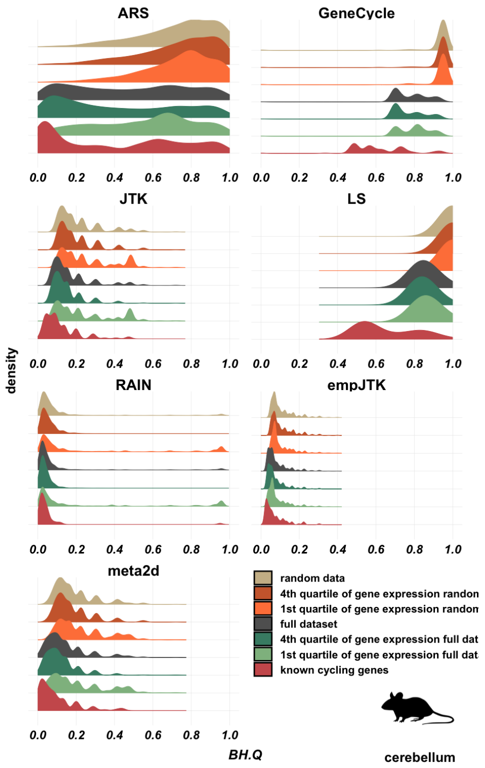

Fig. S78

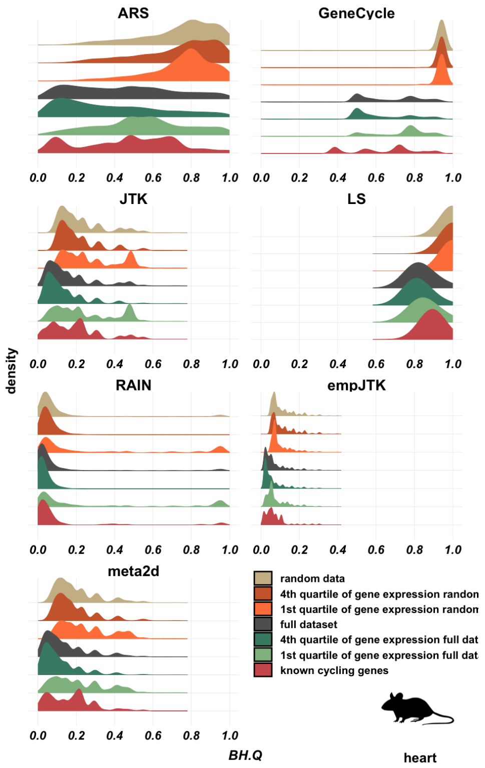

Fig. S78

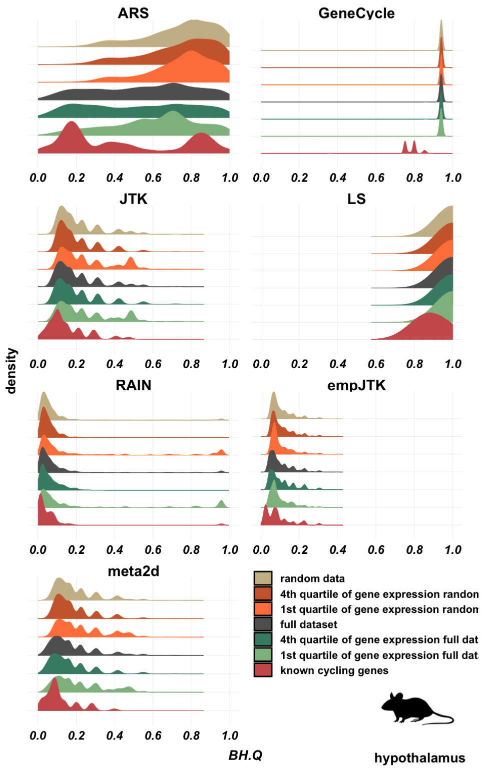

Fig. S78

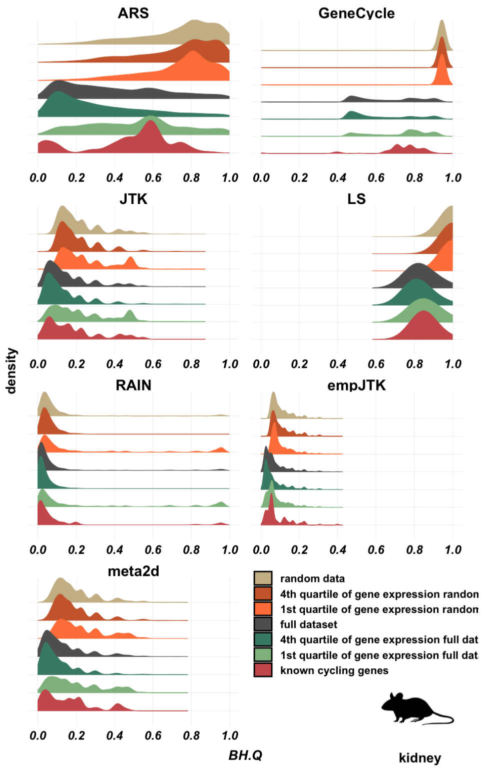

Fig. S78

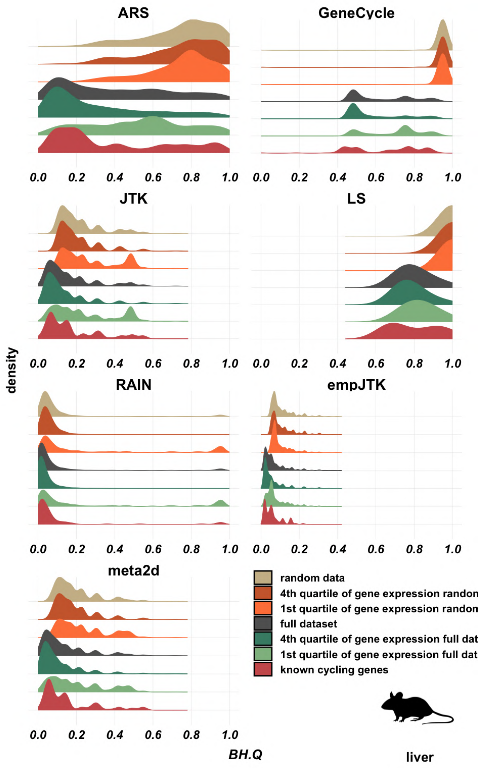

Fig. S78

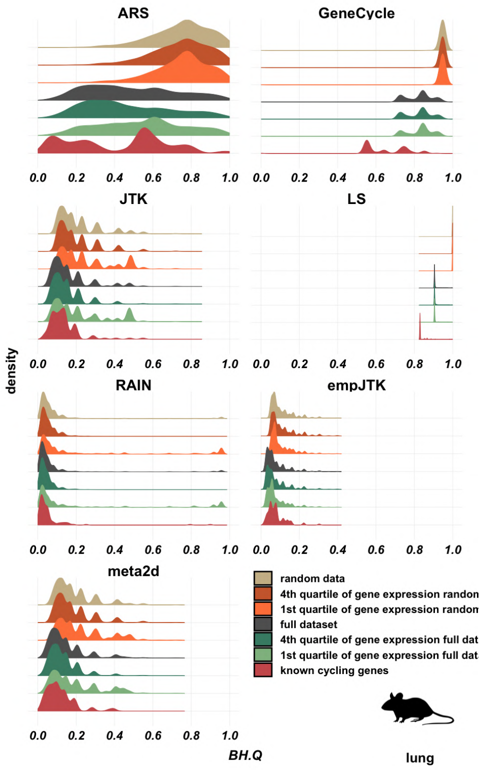

Fig. S78

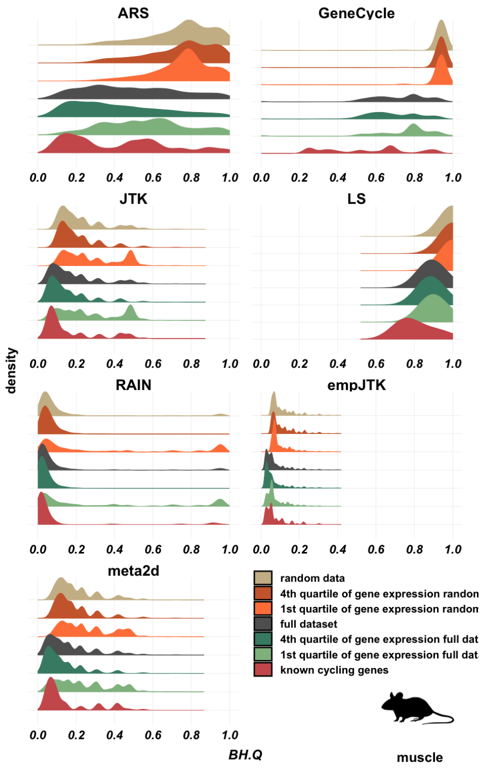

Fig. S78

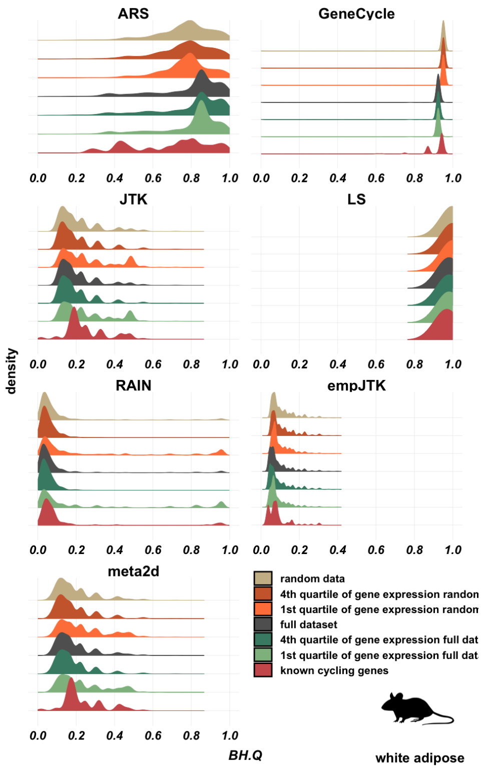

Fig. S78

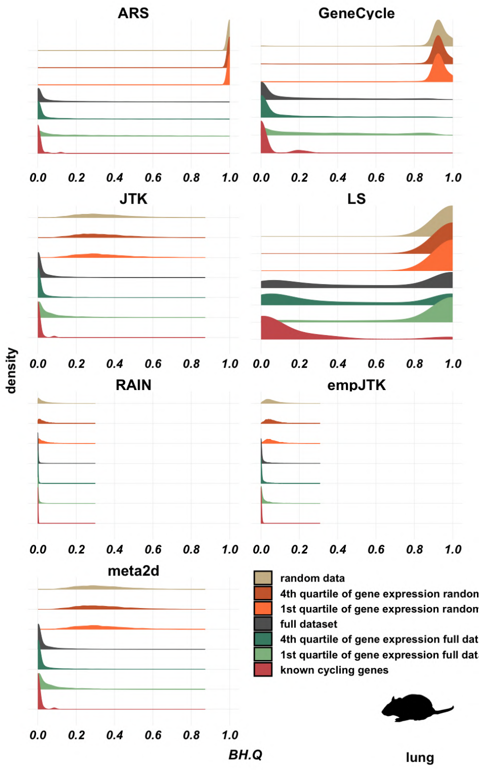

Fig. S78

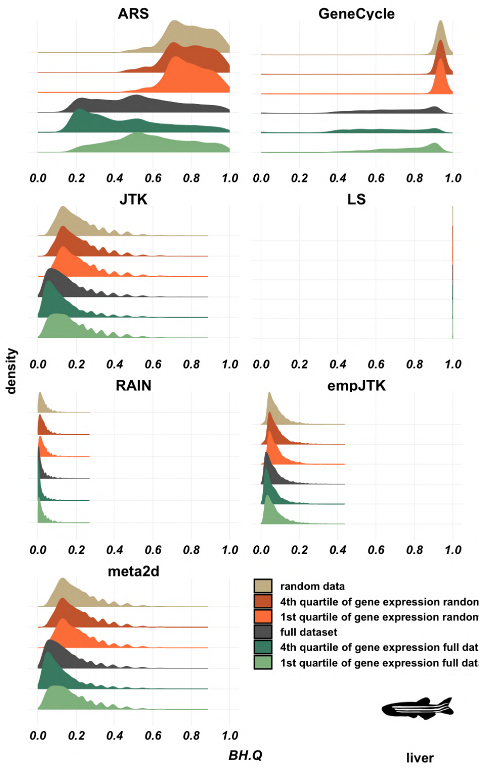

ARS
GeneCycle
JTK
LS
RAIN
empJTK
meta2d
density
0.0
0.2
0.4
0.6
0.8
1.0
BH.Q
random data
4th quartile of gene expression randon
1st quartile of gene expression randon
full dataset
4th quartile of gene expression full dat
1st quartile of gene expression full dat
liver

Fig. S78

Images credit: Anthony Caravaggi (mouse), Ian Quigley (zebrafish) both license CC BY-NC-SA 3.0, Wikipedia GNU GPL Muhammad Mahdi Karim (baboon), and Public Domain for other images (from http://phylopic.org/)
