# Supplementary material for: Methods detecting rhythmic gene expression are biologically relevant only for strong signal
Source: PLoS Comput Biol. 2020 Mar 17;16(3):e1007666. doi: 10.1371/journal.pcbi.1007666 (PMC7100990; doi:10.1371/journal.pcbi.1007666)
Supplement: S4 File — p-values density distribution of rhythmic orthologs vs non-rhythmic orthologs obtained for the seven methods applied to different vertebrate datasets. Orthologous genes detected as rhythmic in the same organ of two species have a stronger statistical signal of rhythmicity than those detected as not-rhythmic in at least one species. From all species_1 genes, only species_1-species_2 one-to-one orthologs are kept. Considering homologous tissues, these orthologs are separated into two groups: genes for which the ortholog is detected as rhythmic in this tissue of species_2, called rhythmic orthologs; and the remaining one-to-one orthologs. (PDF) [file pcbi.1007666.s005.pdf]

VERTEBRATES

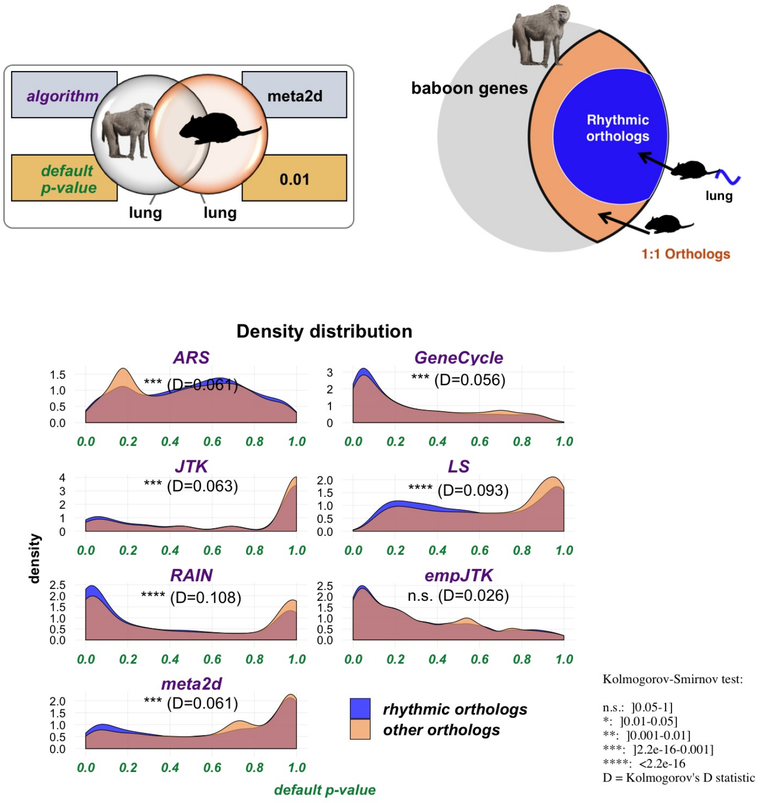

Fig. S1

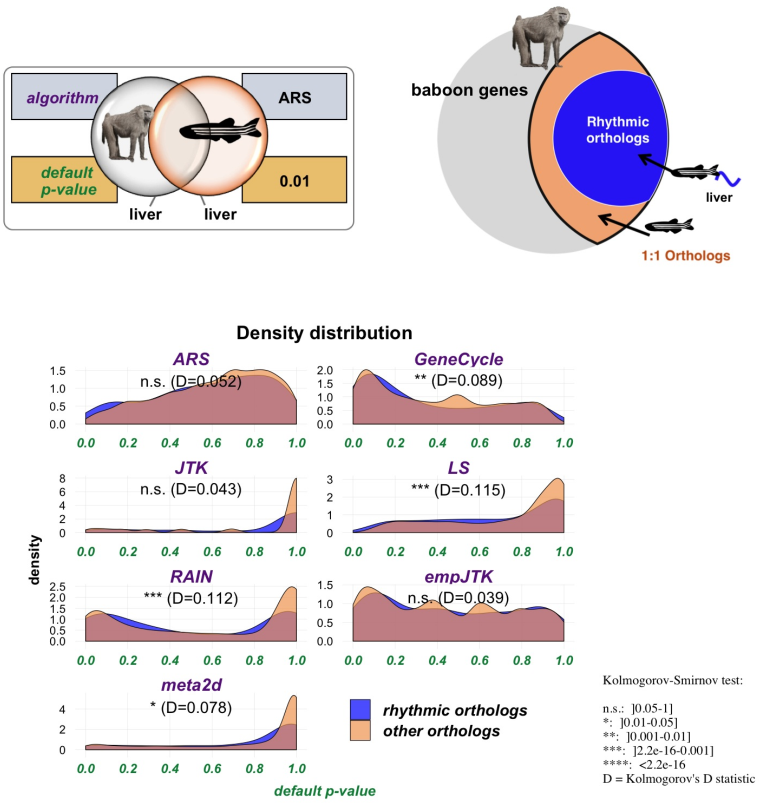

Fig. S2

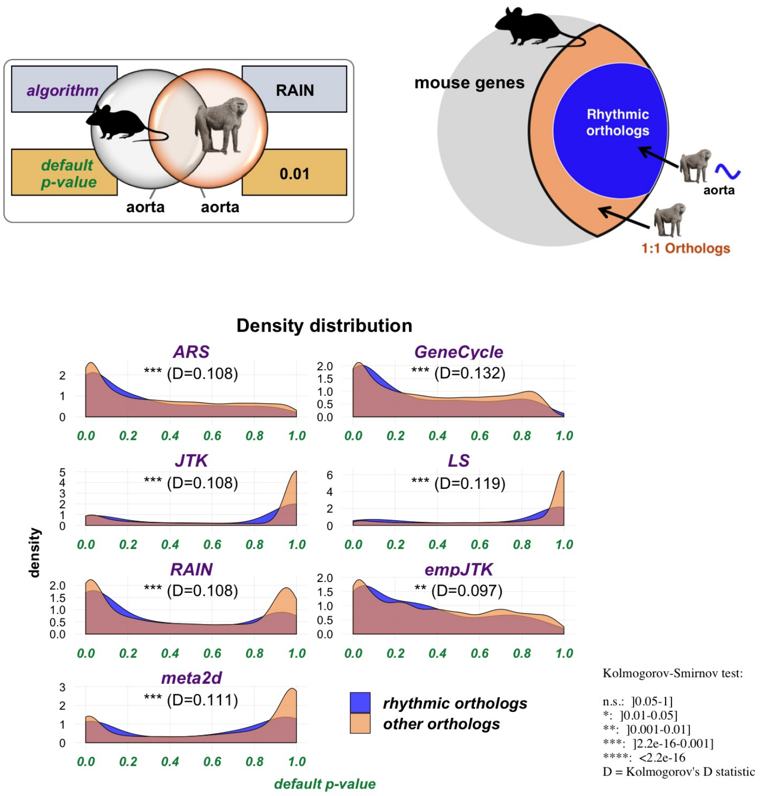
algorithm
RAIN
default p-value
0.01
aorta
aorta
mouse genes
Rhythmic orthologs
aorta
1:1 Orthologs
Density distribution
ARS
*** (D=0.108)
GeneCycle
*** (D=0.132)
JTK
*** (D=0.108)
LS
*** (D=0.119)
RAIN
*** (D=0.108)
empJTK
** (D=0.097)
meta2d
*** (D=0.111)
density
default p-value
rhythmic orthologs
other orthologs
Kolmogorov-Smirnov test:
n.s.:]0.05-1]
*:]0.01-0.05]
**:]0.001-0.01]
***:]2.2e-16-0.001]
****: <2.2e-16
D = Kolmogorov's D statistic

Fig. S3

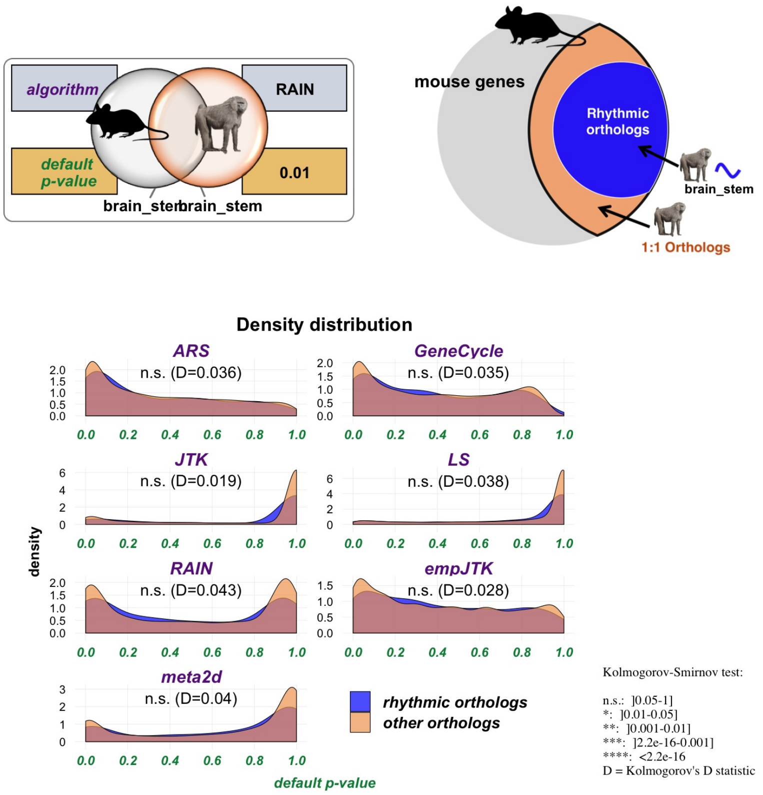
algorithm
RAIN
default p-value
0.01
brain_stem
brain_stem
mouse genes
Rhythmic orthologs
brain_stem
1:1 Orthologs
Density distribution
ARS
n.s. (D=0.036)
GeneCycle
n.s. (D=0.035)
JTK
n.s. (D=0.019)
LS
n.s. (D=0.038)
RAIN
n.s. (D=0.043)
empJTK
n.s. (D=0.028)
meta2d
n.s. (D=0.04)
density
default p-value
rhythmic orthologs
other orthologs
Kolmogorov-Smirnov test:
n.s.:]0.05-1]
*:]0.01-0.05]
**:]0.001-0.01]
***:]2.2e-16-0.001]
****: <2.2e-16
D = Kolmogorov's D statistic

Fig. S4

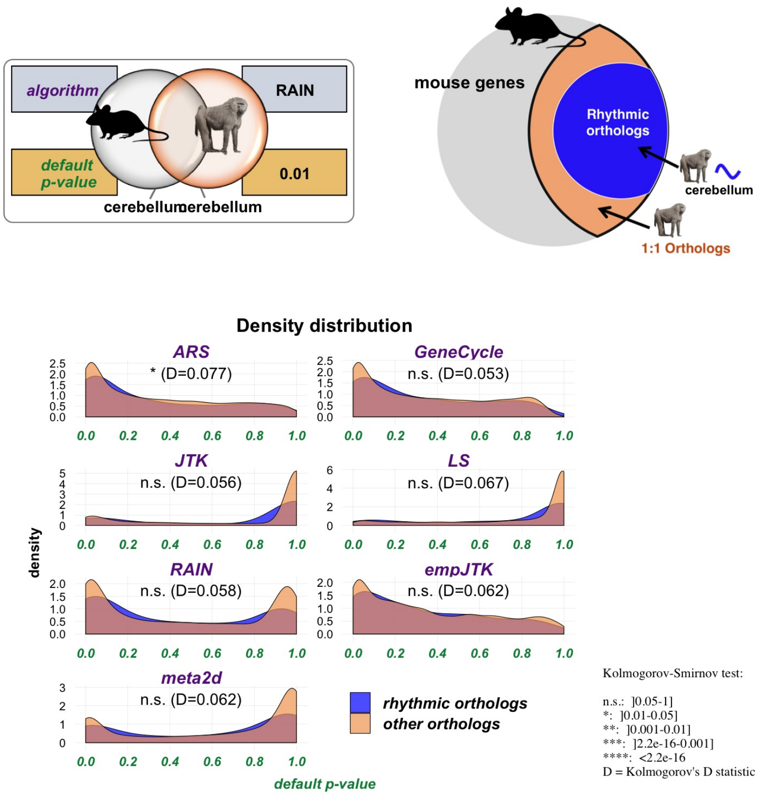
algorithm
RAIN
default p-value
0.01
cerebellum
cerebellum
mouse genes
Rhythmic orthologs
cerebellum
1:1 Orthologs
Density distribution
ARS
* (D=0.077)
GeneCycle
n.s. (D=0.053)
JTK
n.s. (D=0.056)
LS
n.s. (D=0.067)
RAIN
n.s. (D=0.058)
empJTK
n.s. (D=0.062)
meta2d
n.s. (D=0.062)
density
default p-value
rhythmic orthologs
other orthologs
Kolmogorov-Smirnov test:
n.s.:]0.05-1]
*:]0.01-0.05]
**:]0.001-0.01]
***:]2.2e-16-0.001]
****: <2.2e-16
D = Kolmogorov's D statistic

Fig. S5

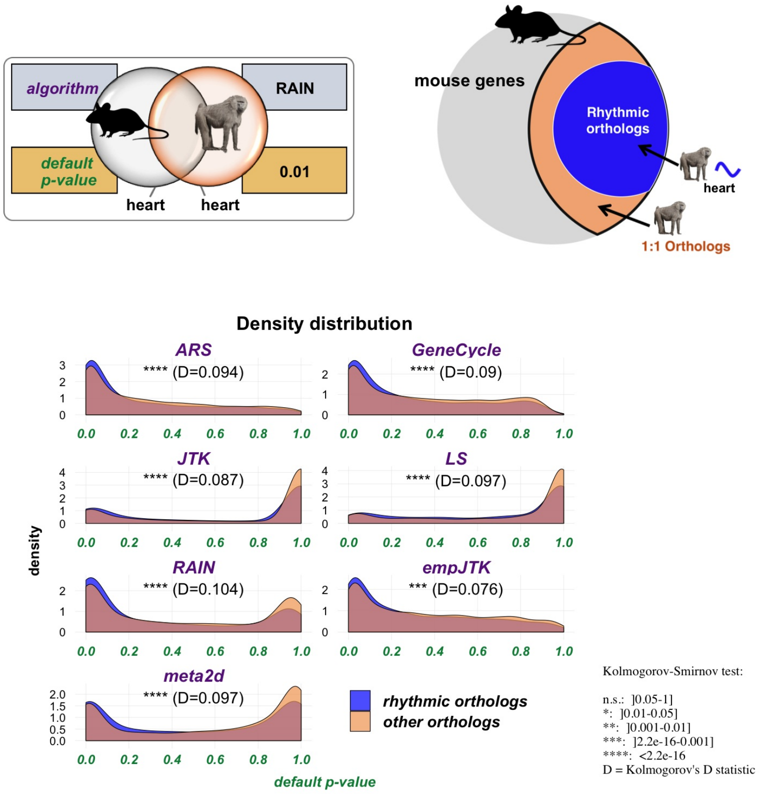
algorithm
RAIN
default p-value
0.01
heart
heart
mouse genes
Rhythmic orthologs
heart
1:1 Orthologs
Density distribution
ARS
**** (D=0.094)
GeneCycle
**** (D=0.09)
JTK
**** (D=0.087)
LS
**** (D=0.097)
RAIN
**** (D=0.104)
empJTK
*** (D=0.076)
meta2d
**** (D=0.097)
density
default p-value
rhythmic orthologs
other orthologs
Kolmogorov-Smirnov test:
n.s.:]0.05-1]
*:]0.01-0.05]
**:]0.001-0.01]
***:]2.2e-16-0.001]
****: <2.2e-16
D = Kolmogorov's D statistic

Fig. S6

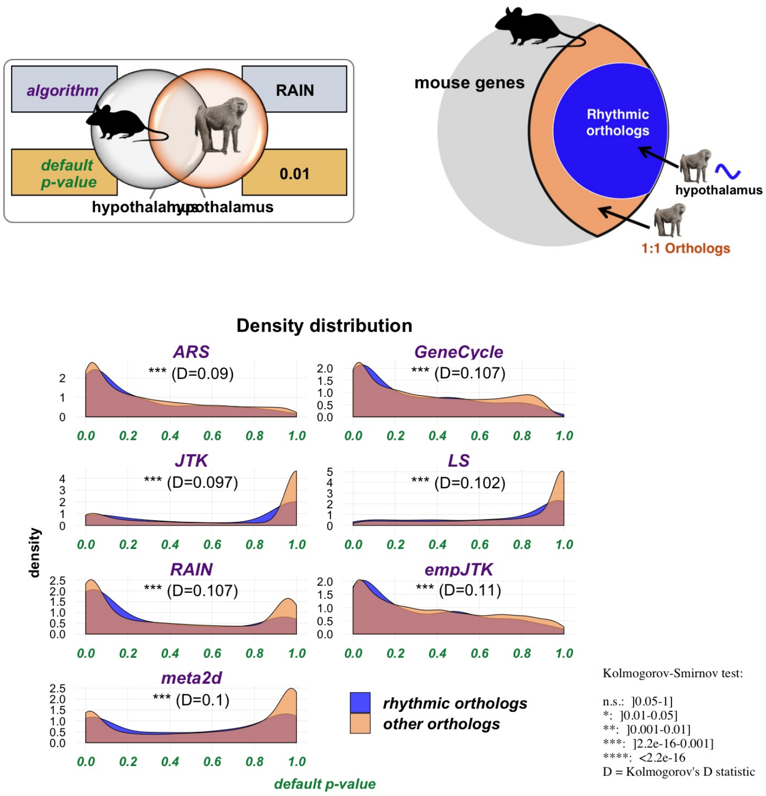
algorithm
RAIN
default p-value
0.01
hypothalamus
hypothalamus
mouse genes
Rhythmic orthologs
hypothalamus
1:1 Orthologs
Density distribution
ARS
*** (D=0.09)
GeneCycle
*** (D=0.107)
JTK
*** (D=0.097)
LS
*** (D=0.102)
RAIN
*** (D=0.107)
empJTK
*** (D=0.11)
meta2d
*** (D=0.1)
density
default p-value
rhythmic orthologs
other orthologs
Kolmogorov-Smirnov test:
n.s.:]0.05-1]
*:]0.01-0.05]
**:]0.001-0.01]
***:]2.2e-16-0.001]
****: <2.2e-16
D = Kolmogorov's D statistic

Fig. S7

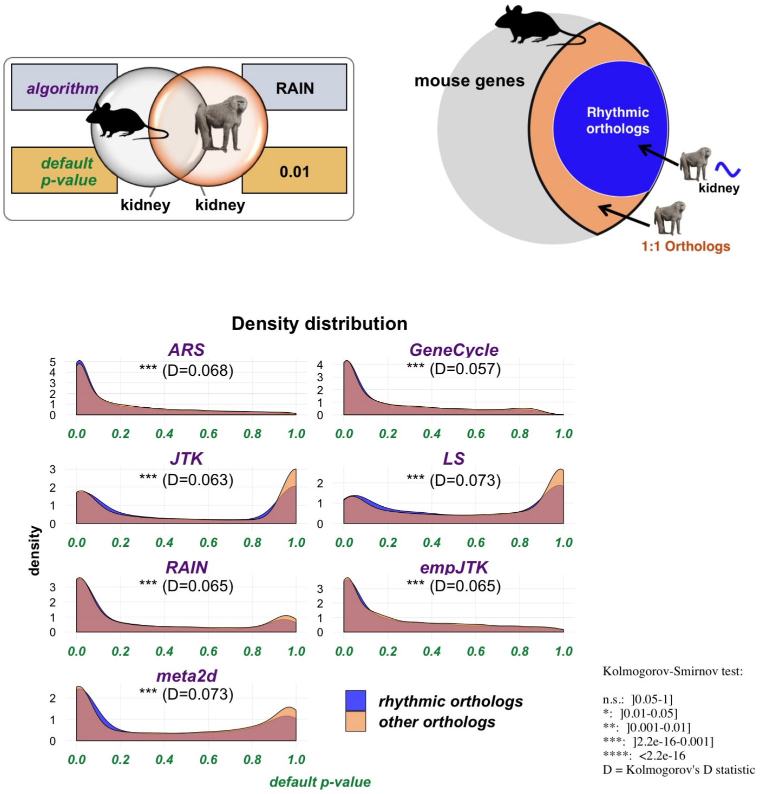
algorithm
RAIN
default p-value
0.01
kidney
kidney
mouse genes
Rhythmic orthologs
kidney
1:1 Orthologs
Density distribution
ARS
*** (D=0.068)
GeneCycle
*** (D=0.057)
JTK
*** (D=0.063)
LS
*** (D=0.073)
RAIN
*** (D=0.065)
empJTK
*** (D=0.065)
meta2d
*** (D=0.073)
density
default p-value
rhythmic orthologs
other orthologs
Kolmogorov-Smirnov test:
n.s.:]0.05-1]
*:]0.01-0.05]
**:]0.001-0.01]
***:]2.2e-16-0.001]
****: <2.2e-16
D = Kolmogorov's D statistic

Fig. S8

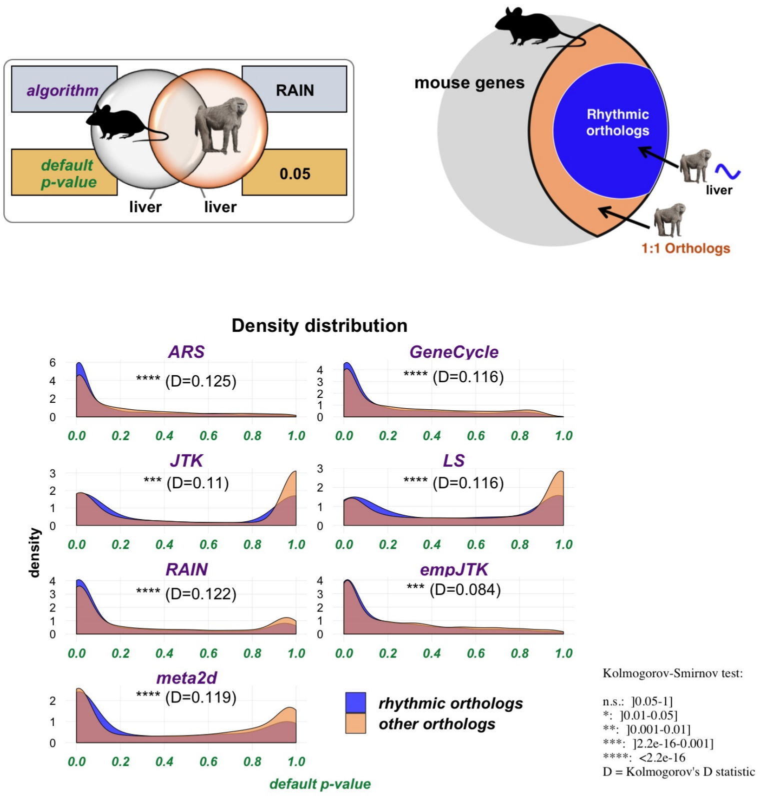

Fig. S9

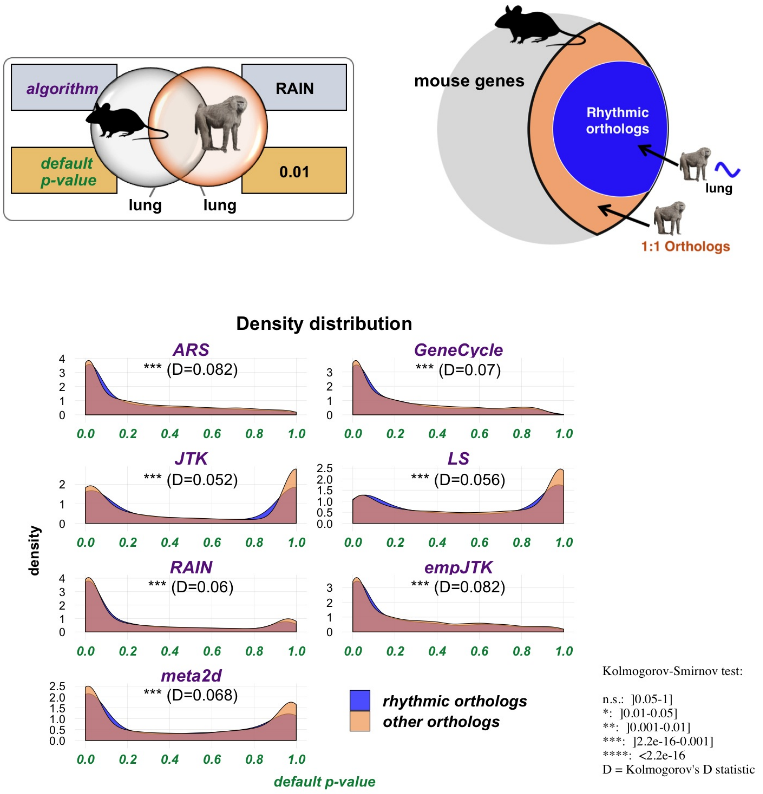

Fig. S10

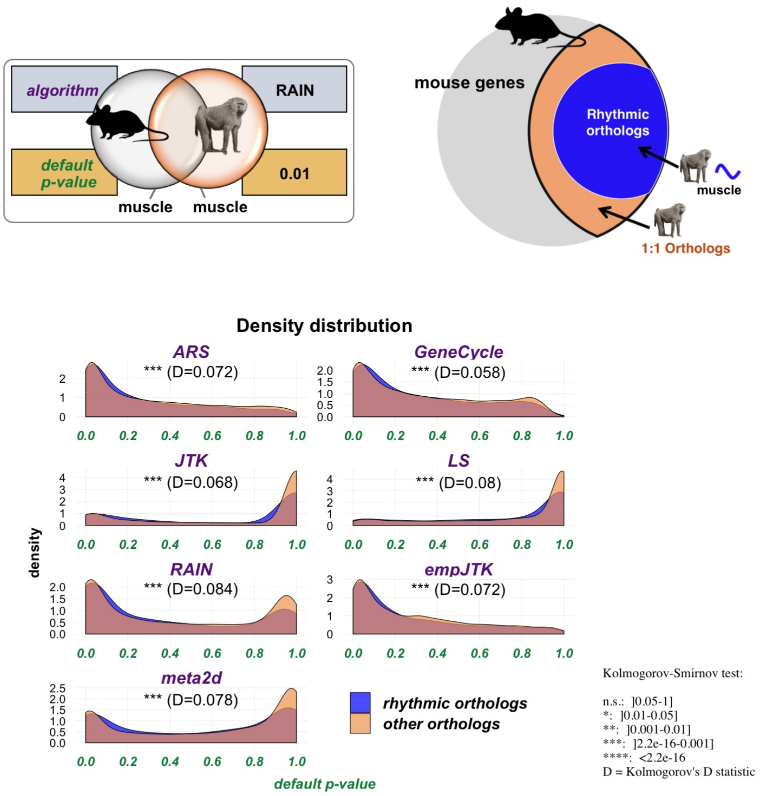

Fig. S11

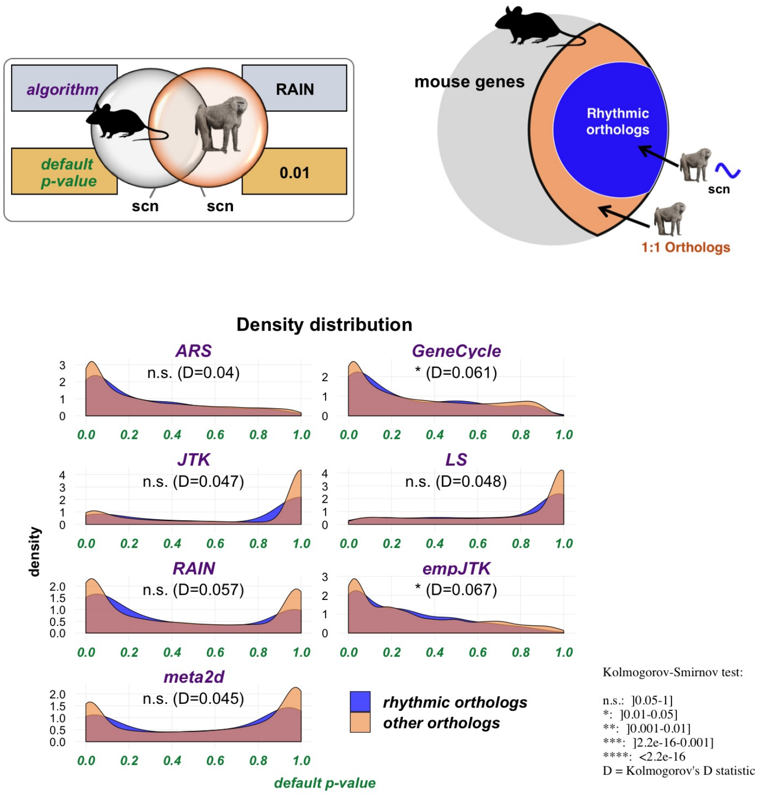

Fig. S12

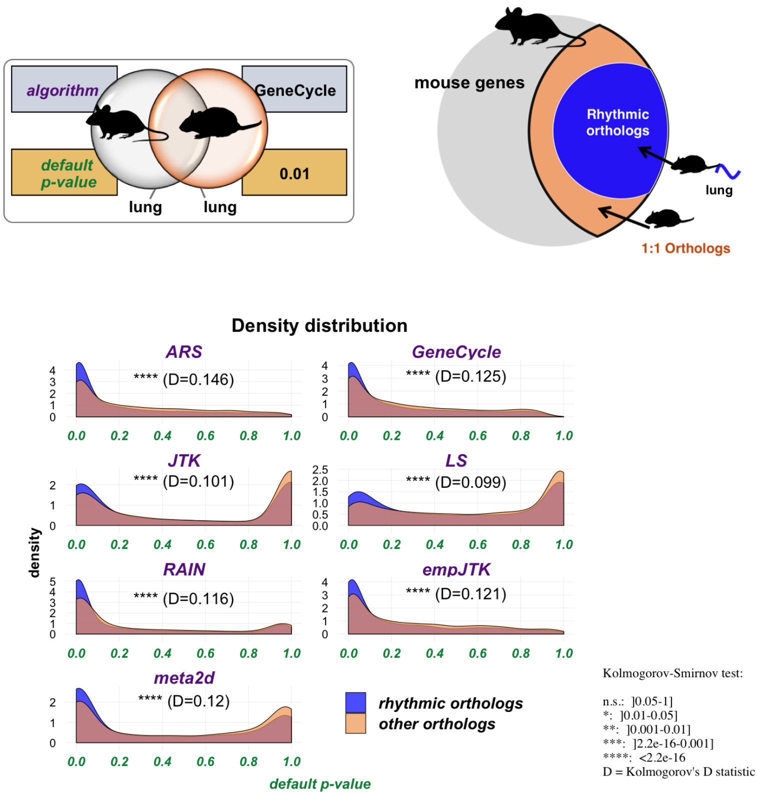

Fig. S13

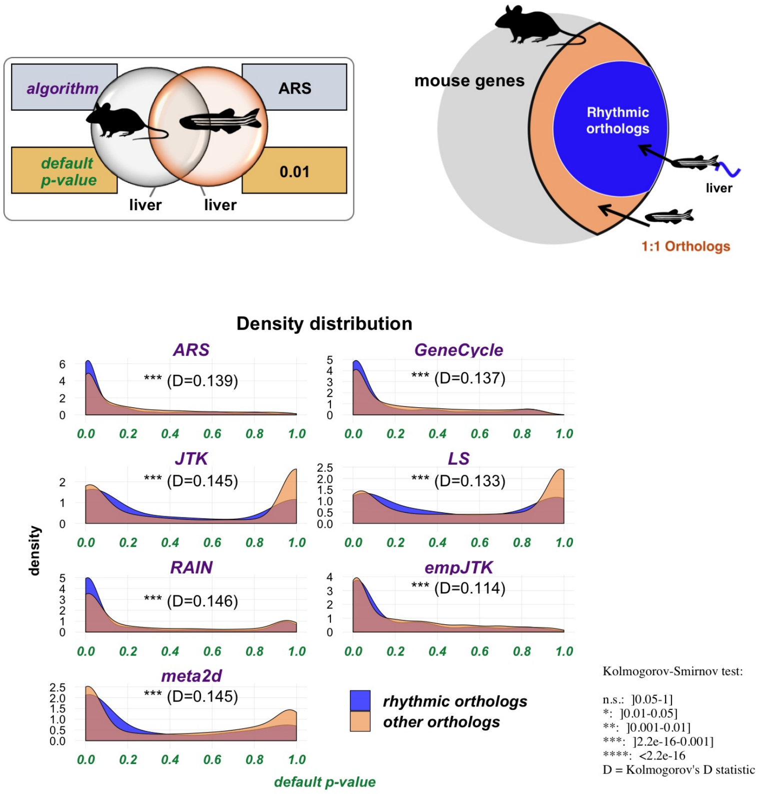

Fig. S14

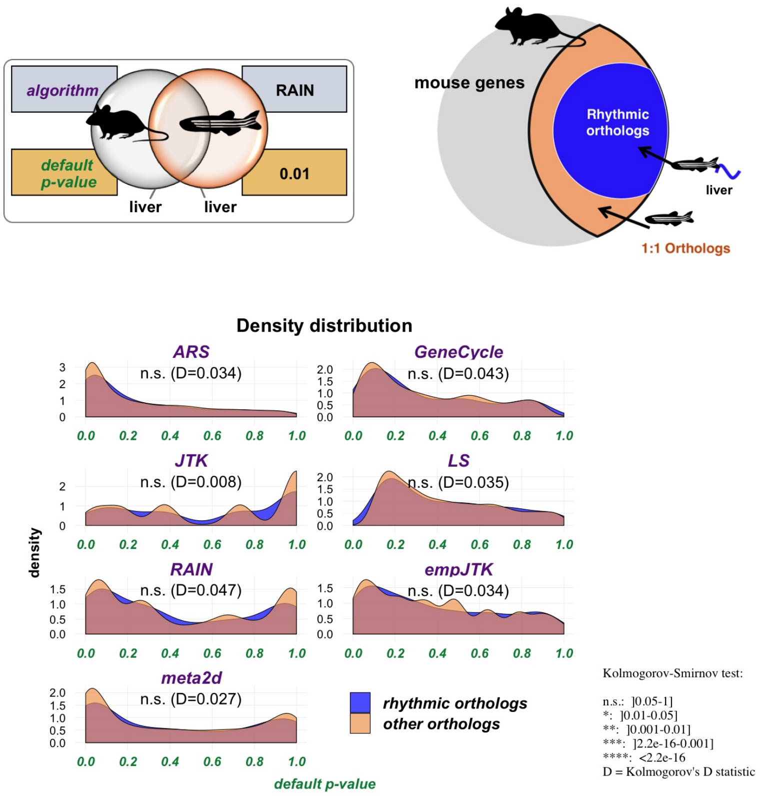

algorithm
RAIN
default p-value
0.01
liver
liver
mouse genes
Rhythmic orthologs
liver
1:1 Orthologs
Density distribution
ARS
n.s. (D=0.034)
GeneCycle
n.s. (D=0.043)
JTK
n.s. (D=0.008)
LS
n.s. (D=0.035)
RAIN
n.s. (D=0.047)
empJTK
n.s. (D=0.034)
meta2d
n.s. (D=0.027)
density
default p-value
rhythmic orthologs
other orthologs
Kolmogorov-Smirnov test:
n.s.:]0.05-1]
*:]0.01-0.05]
**:]0.001-0.01]
***:]2.2e-16-0.001]
****: <2.2e-16
D = Kolmogorov's D statistic

Fig. S15

Images credit: Anthony Caravaggi (mouse), Ian Quigley (zebrafish) both license CC BY-NC-SA 3.0, Wikipedia GNU GPL Muhammad Mahdi Karim (baboon), and Public Domain for other images (from http://phylopic.org/)
