# Supplementary material for: Methods detecting rhythmic gene expression are biologically relevant only for strong signal
Source: PLoS Comput Biol. 2020 Mar 17;16(3):e1007666. doi: 10.1371/journal.pcbi.1007666 (PMC7100990; doi:10.1371/journal.pcbi.1007666)
Supplement: S5 File — The benchmark gene set is composed of species_1-species_2 orthologs, detected rhythmic in the homologous tissue of species_2 by the ARS, GeneCycle, or empJTK method with default p-value ≤ 0.01 or 0.05. See Fig 6 for definitions of sets A and B. The black line is the Naive method which orders genes according to their median expression levels (median of time-points), from highest expressed to lowest expressed gene, then, for each gene, calculates the proportion of rhythmic orthologs among those with higher expression. (PDF) [file pcbi.1007666.s006.pdf]

VERTEBRATES

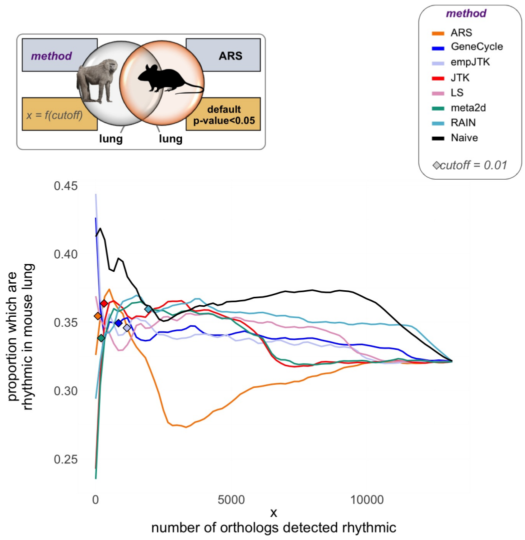

Fig. S1

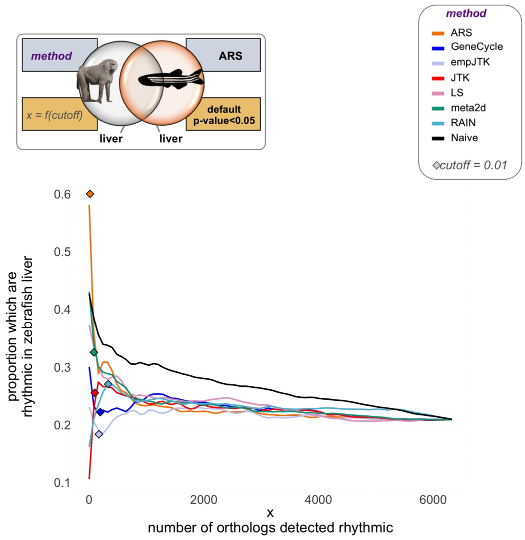

Fig. S2

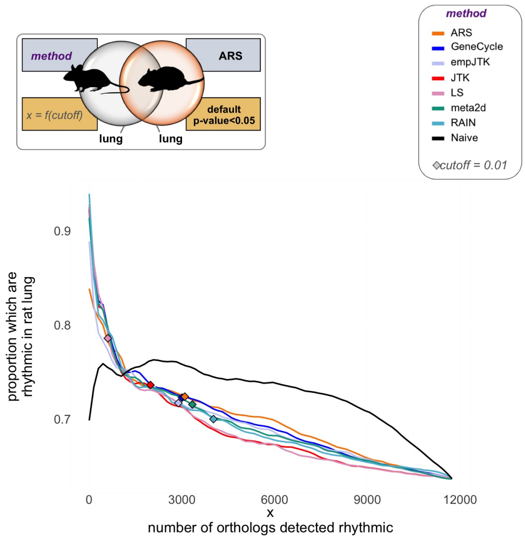

Fig. S3

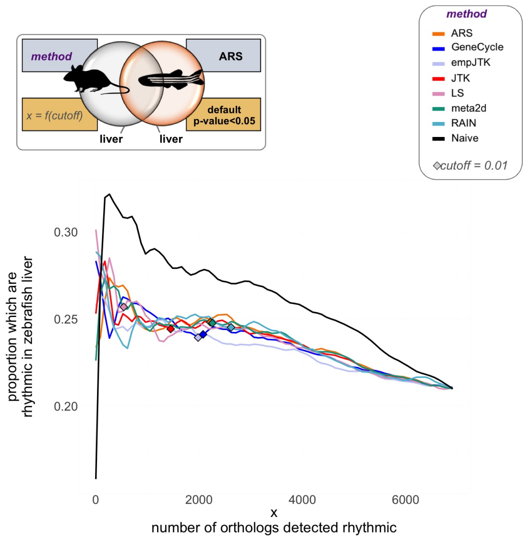

Fig. S4

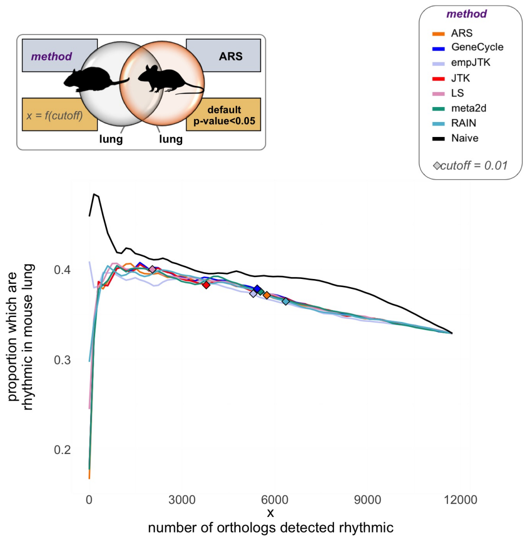

Fig. S5

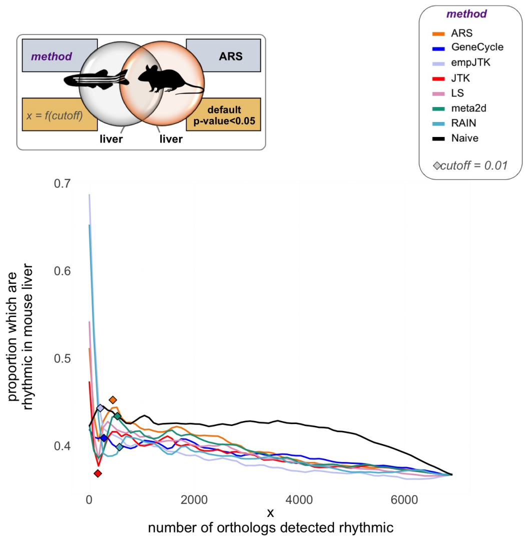

Fig. S6

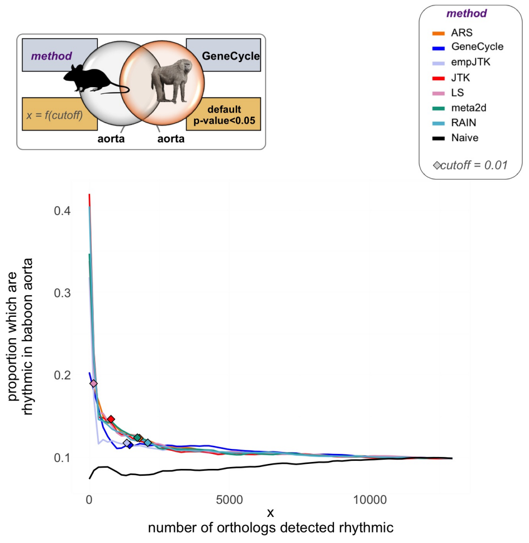

Fig. S7

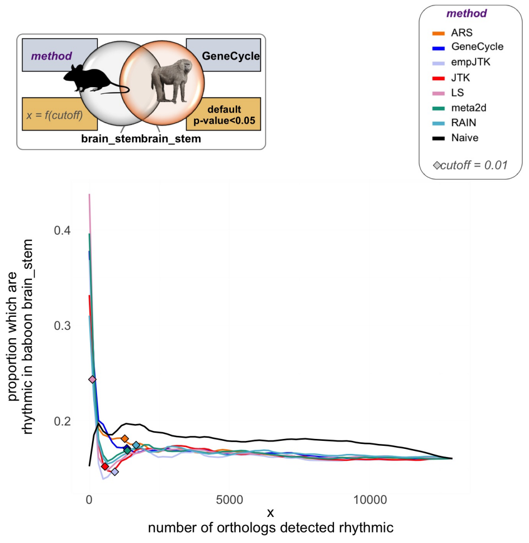

Fig. S8

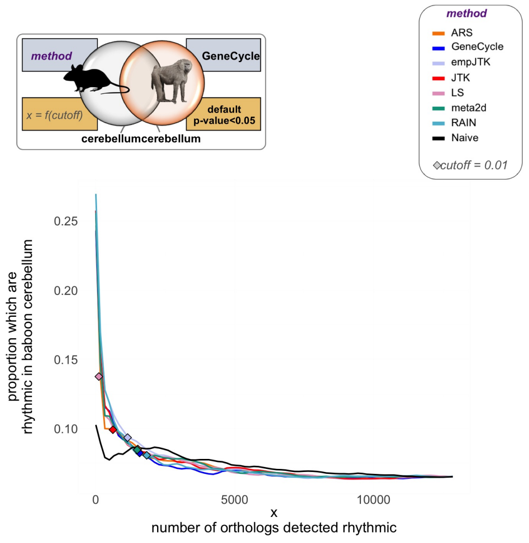

Fig. S9

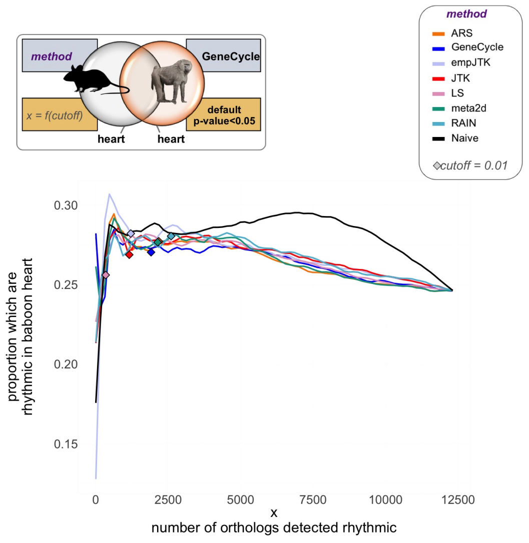

Fig. S10

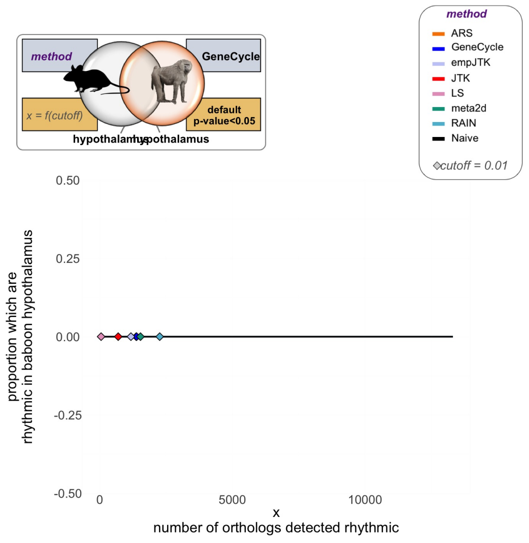

Fig. S11

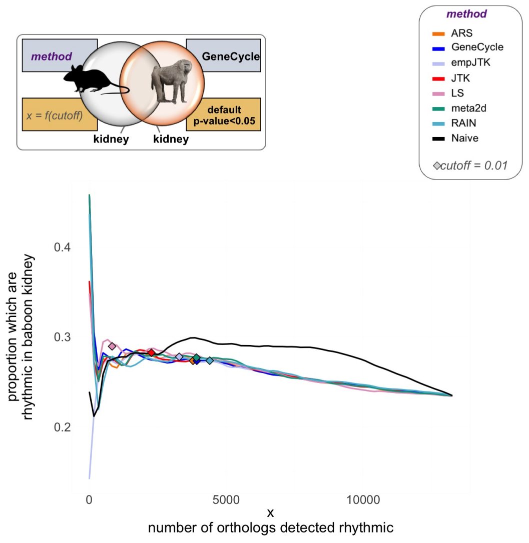

Fig. S12

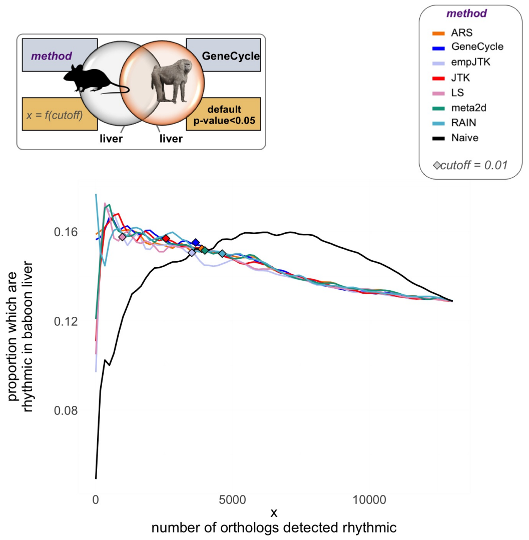

Fig. S13

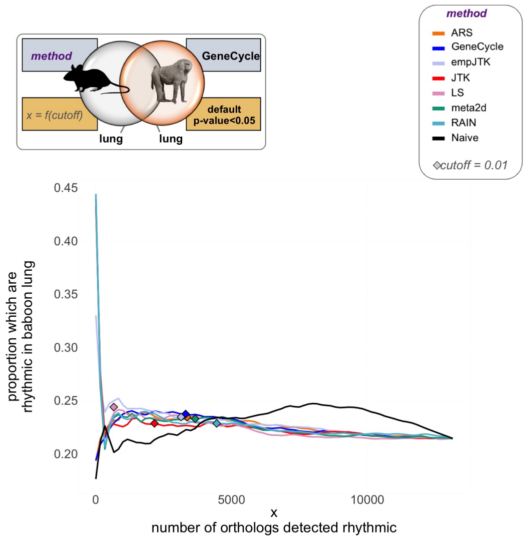

Fig. S14

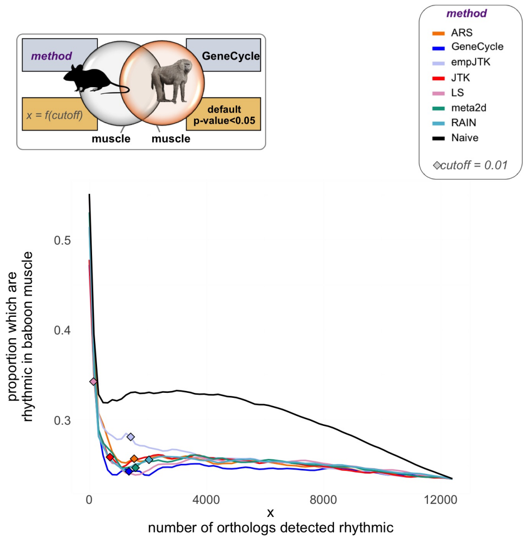

Fig. S15

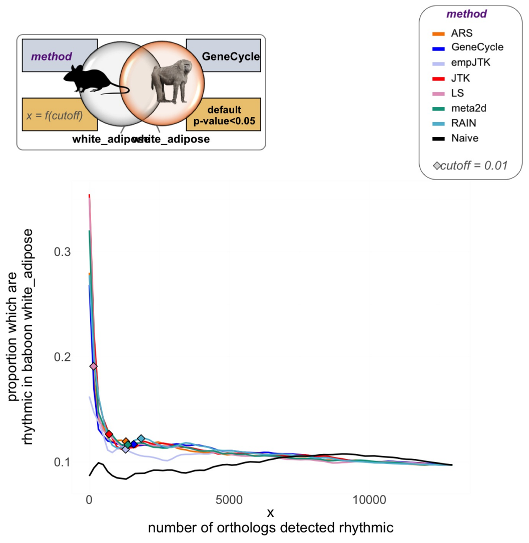

Fig. S16

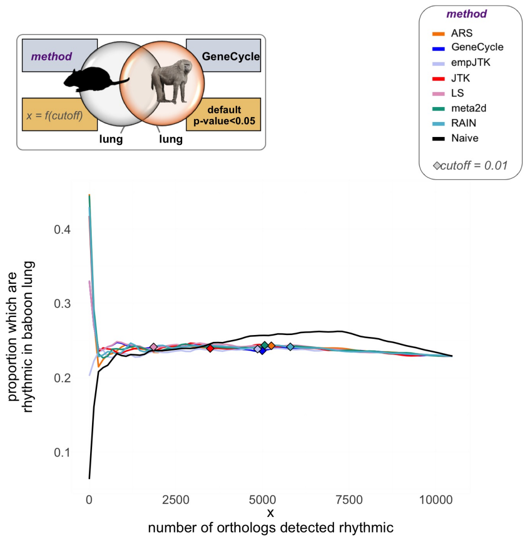

Fig. S17

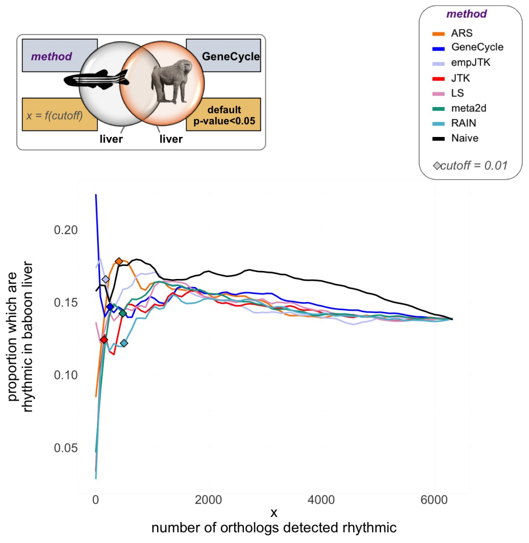

Fig. S18

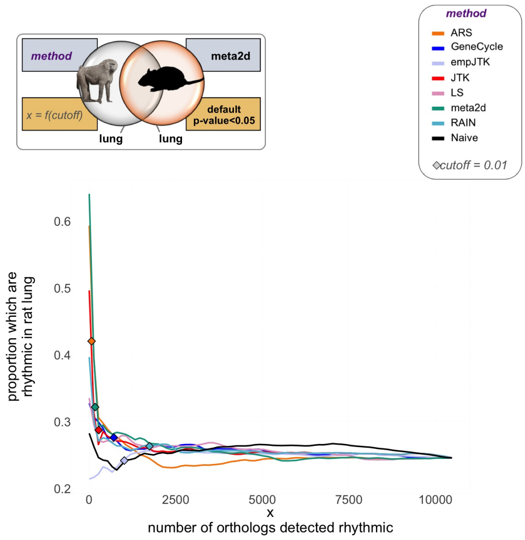
method
meta2d
x = f(cutoff)
default
p-value<0.05
lung
lung
method
ARS
GeneCycle
empJTK
JTK
LS
meta2d
RAIN
Naive
cutoff = 0.01
proportion which are
rhythmic in rat lung
0.6
0.5
0.4
0.3
0.2
0
2500
5000
7500
10000
x
number of orthologs detected rhythmic

Fig. S19

Images credit: Anthony Caravaggi (mouse), Ian Quigley (zebrafish) both license CC BY-NC-SA 3.0, Wikipedia GNU GPL Muhammad Mahdi Karim (baboon), and Public Domain for other images (from http://phylopic.org/)
