# Supplementary material for: Methods detecting rhythmic gene expression are biologically relevant only for strong signal
Source: PLoS Comput Biol. 2020 Mar 17;16(3):e1007666. doi: 10.1371/journal.pcbi.1007666 (PMC7100990; doi:10.1371/journal.pcbi.1007666)
Supplement: S6 File — (PDF) [file pcbi.1007666.s007.pdf]

INSECTS

Density distribution of raw and default *p*-values

default *p*-values

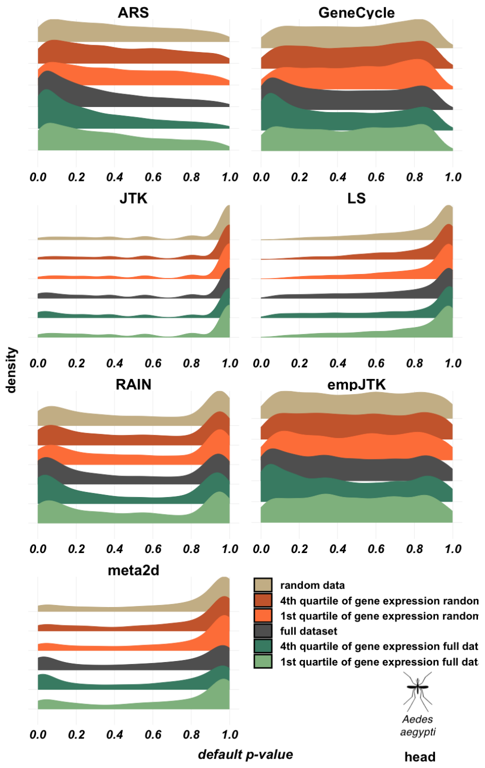

Fig. S1

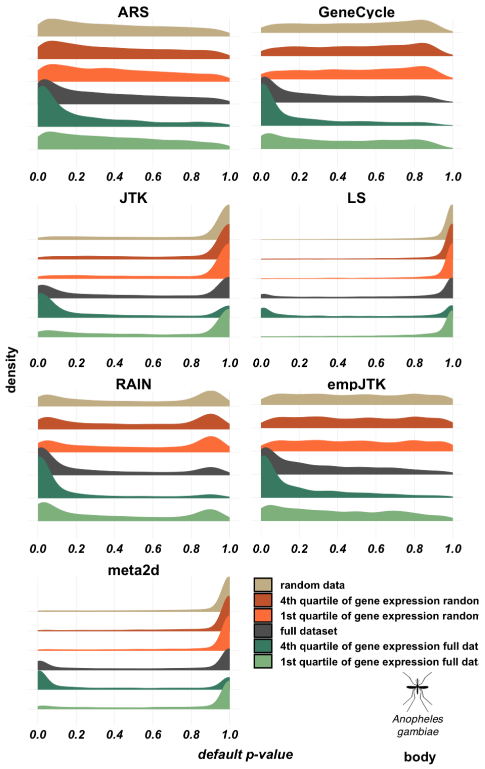

Fig. S2

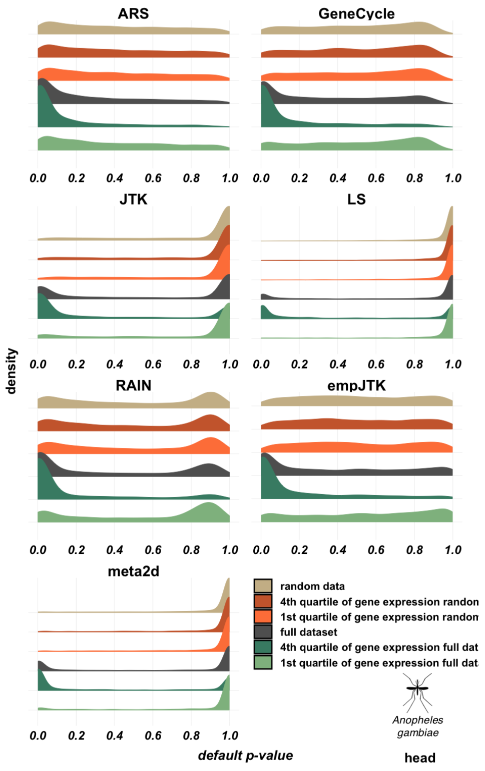
ARS
GeneCycle
JTK
LS
RAIN
empJTK
meta2d
density
0.0 0.2 0.4 0.6 0.8 1.0
default p-value
random data
4th quartile of gene expression randon
1st quartile of gene expression randon
full dataset
4th quartile of gene expression full dat
1st quartile of gene expression full dat
Anopheles gambiae
head

Fig. S3

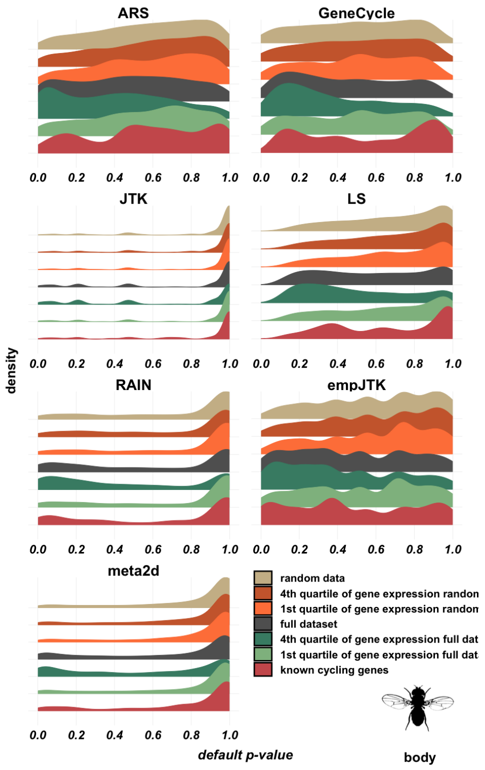
ARS
GeneCycle
JTK
LS
RAIN
empJTK
meta2d
density
0.0 0.2 0.4 0.6 0.8 1.0
default p-value
random data
4th quartile of gene expression randon
1st quartile of gene expression randon
full dataset
4th quartile of gene expression full dat
1st quartile of gene expression full dat
known cycling genes
body

Fig. S4

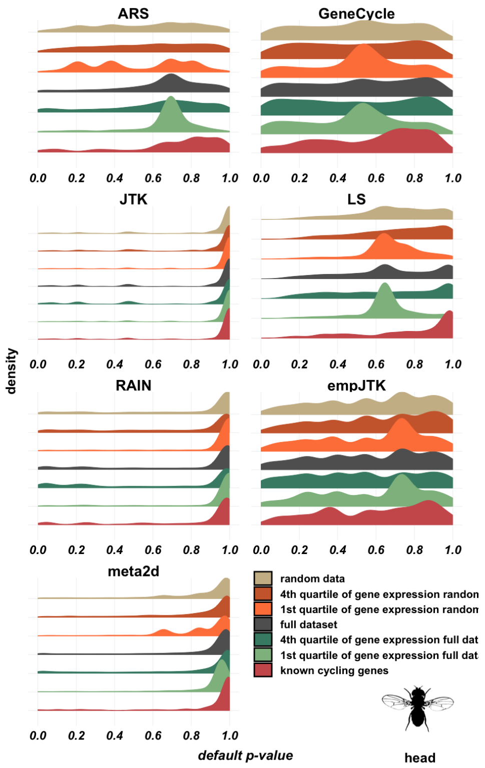

Fig. S5

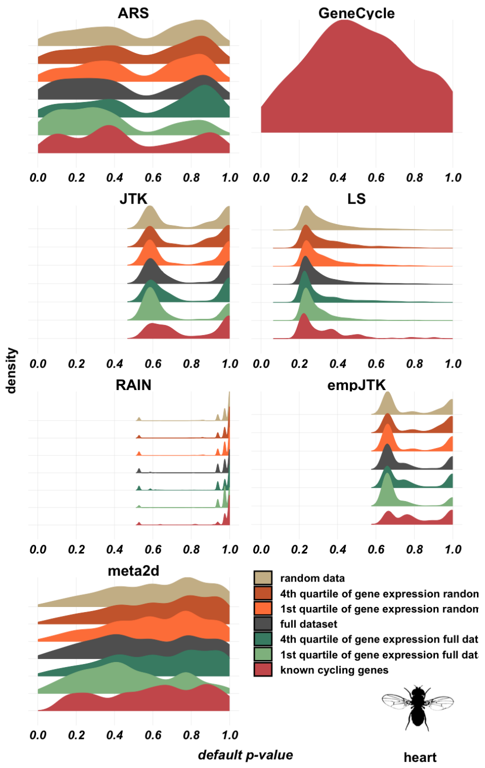

Fig. S6

raw *p*-values

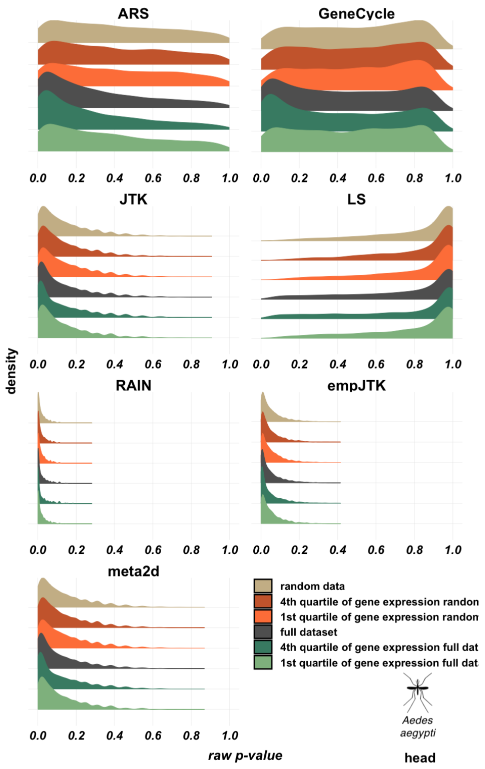
ARS
GeneCycle
JTK
LS
RAIN
empJTK
meta2d
density
0.0
0.2
0.4
0.6
0.8
1.0
random data
4th quartile of gene expression randon
1st quartile of gene expression randon
full dataset
4th quartile of gene expression full dat
1st quartile of gene expression full dat
Aedes aegypti
raw p-value
head

Fig. S7

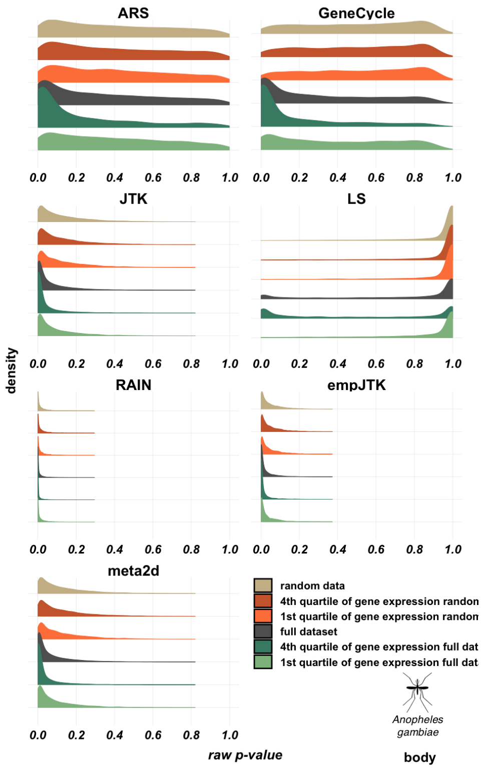
ARS
GeneCycle
JTK
LS
RAIN
empJTK
meta2d
density
0.0
0.2
0.4
0.6
0.8
1.0
random data
4th quartile of gene expression randon
1st quartile of gene expression randon
full dataset
4th quartile of gene expression full dat
1st quartile of gene expression full dat
Anopheles gambiae
raw p-value
body

Fig. S8

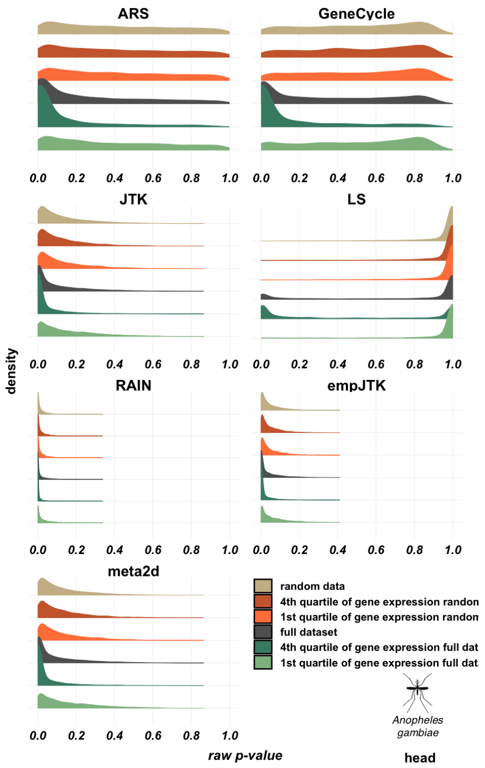

Fig. S9

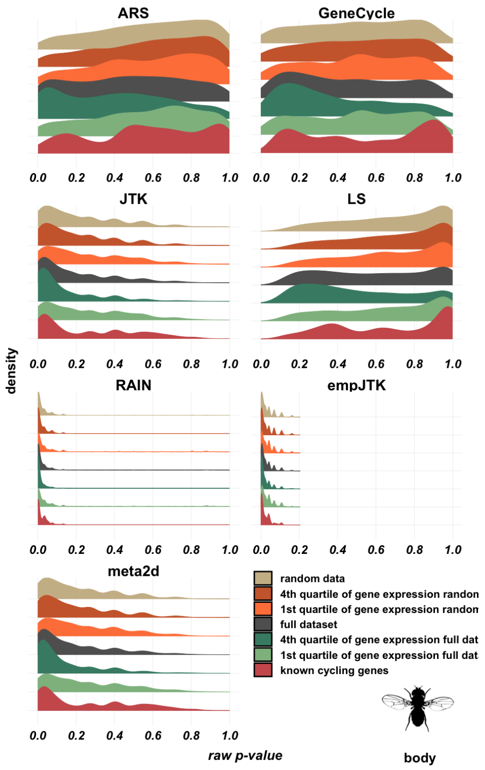

Fig. S10

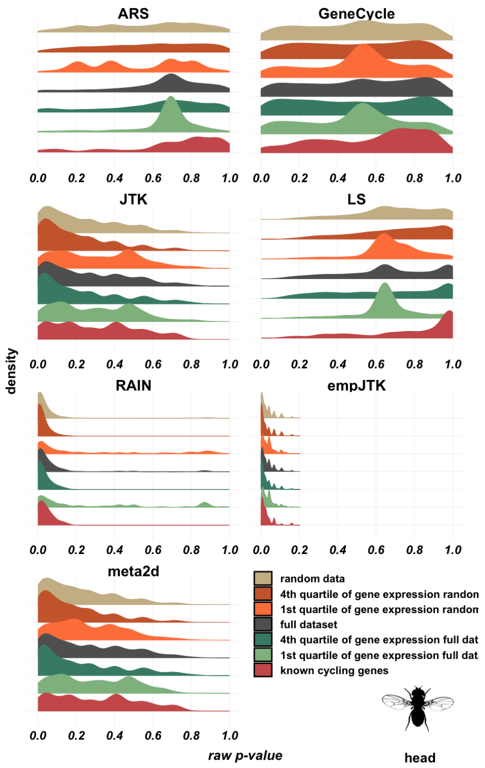

Fig. S11

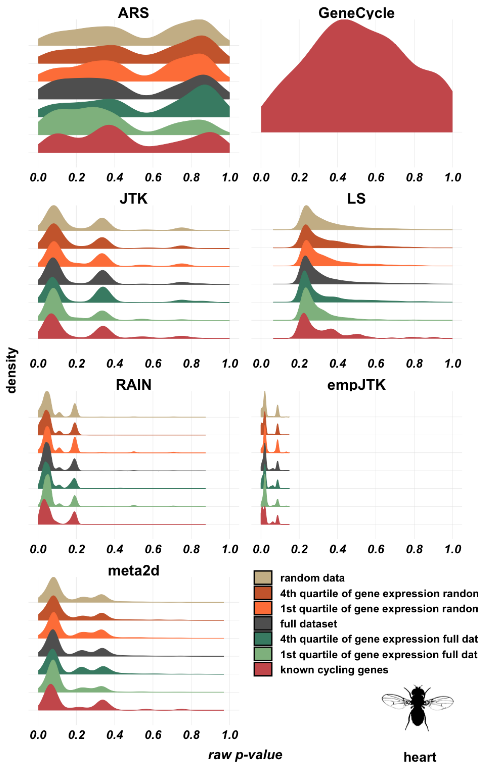

Fig. S12

Density distribution of *p*-values: rhythmic vs non-rhythmic orthologs

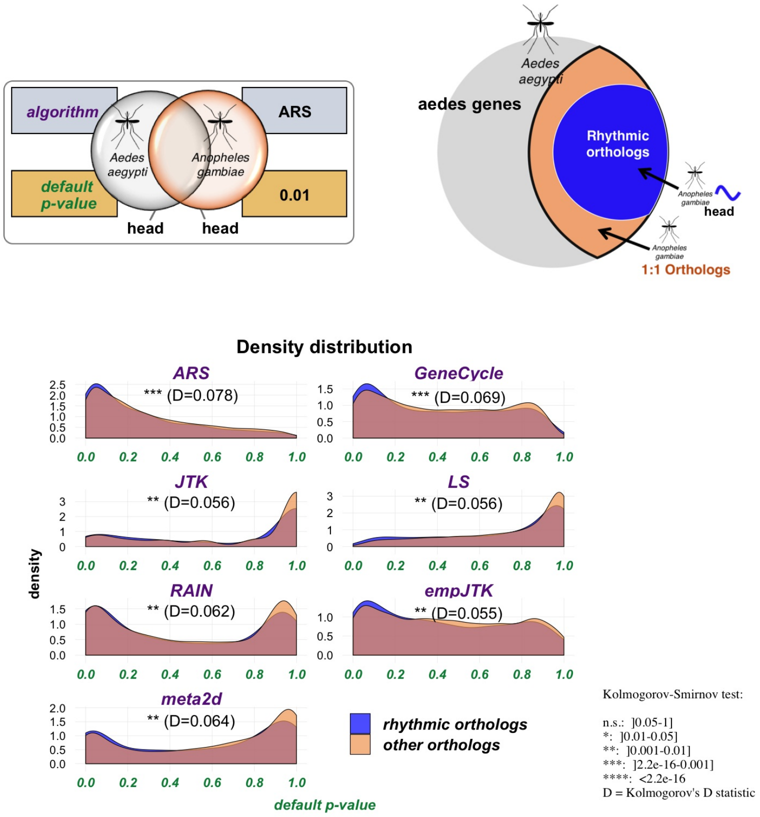

Fig. S13

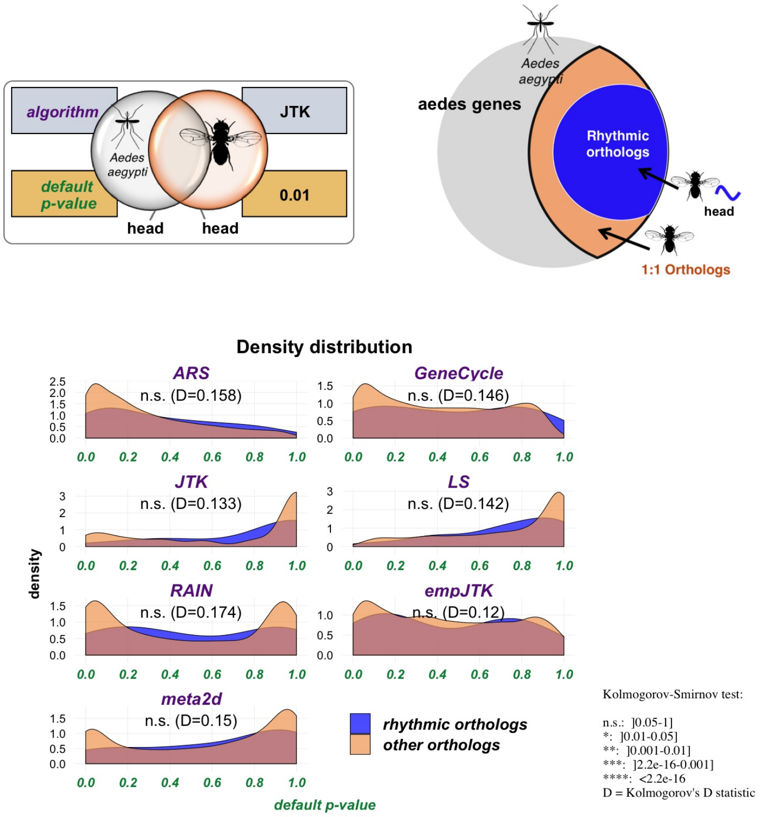

Fig. S14

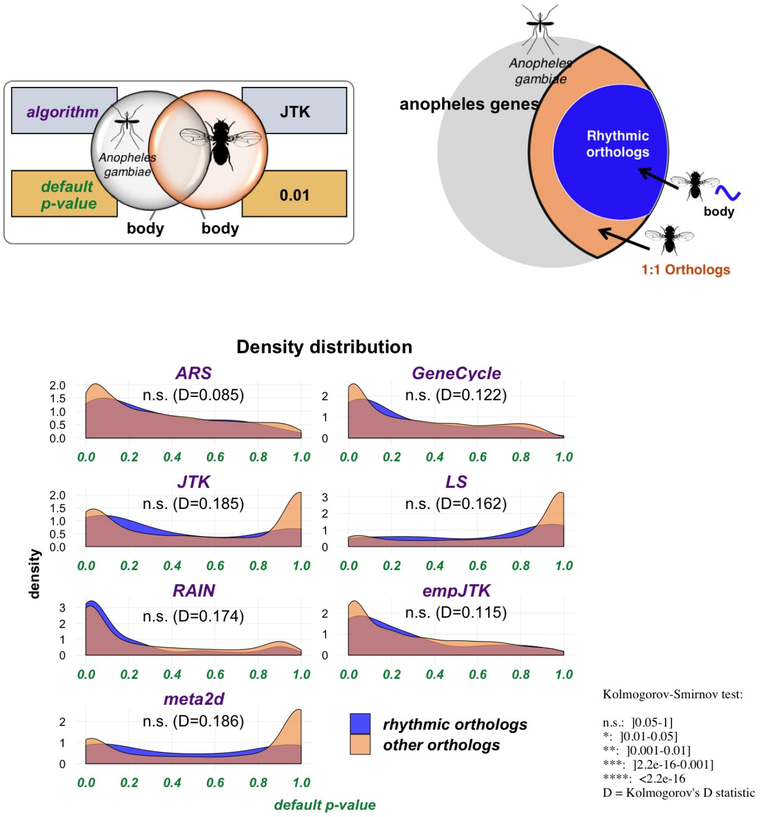

Fig. S15

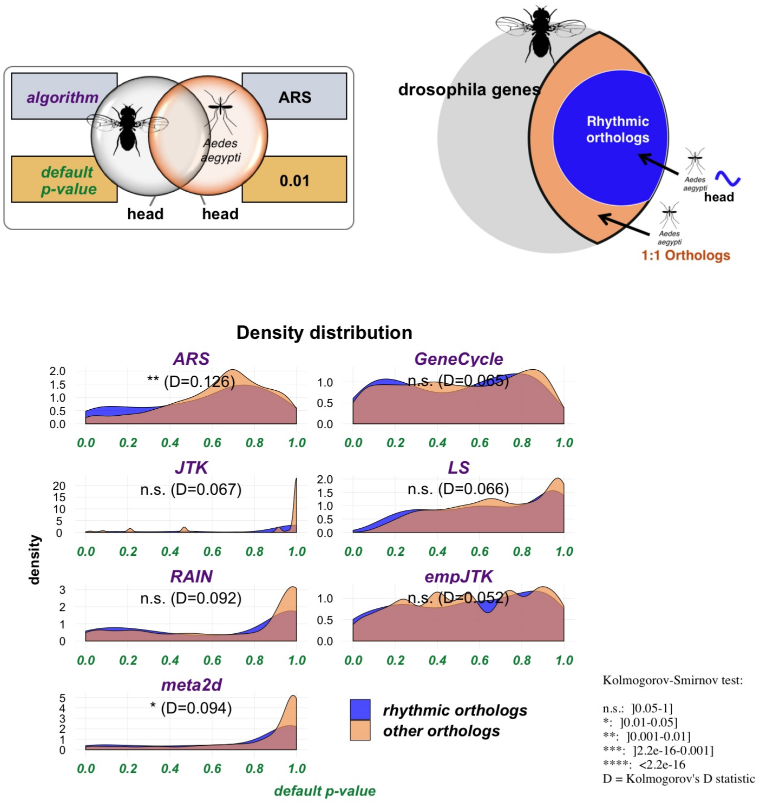

Fig. S16

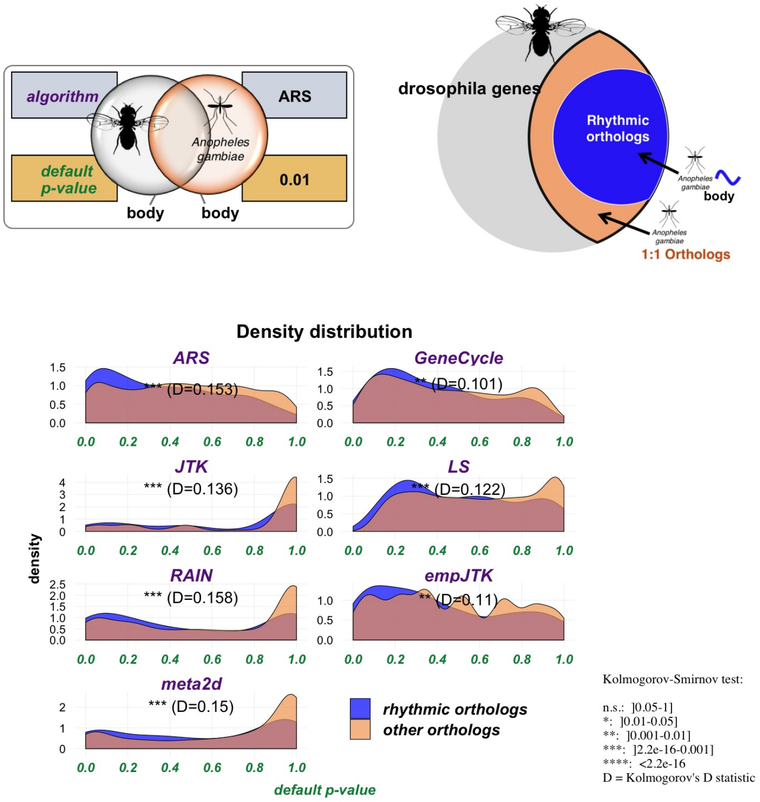

Fig. S17

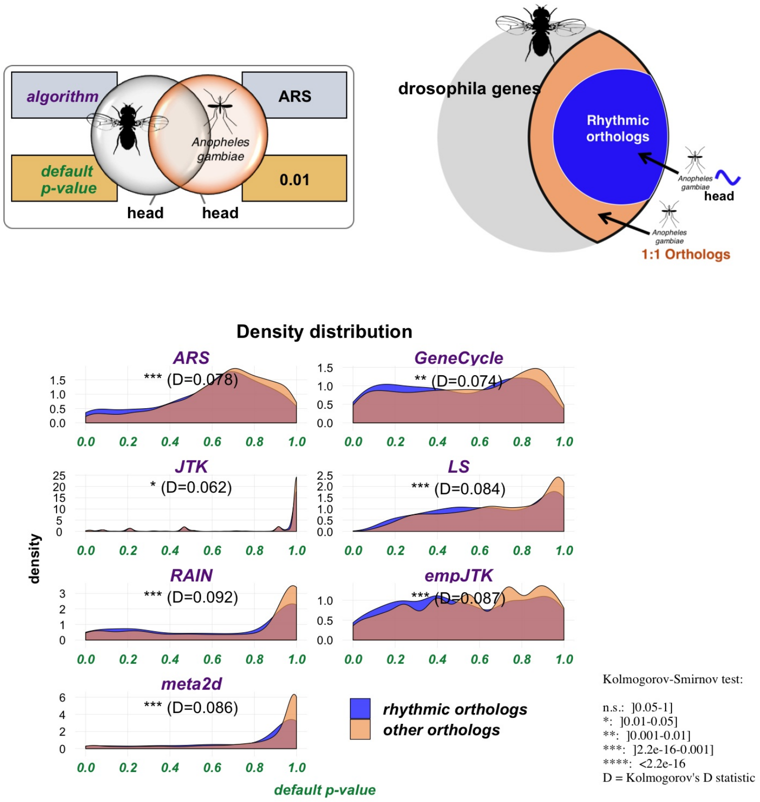

Fig. S18

Variation of the proportion A/B as a function of the number of orthologs detected rhythmic

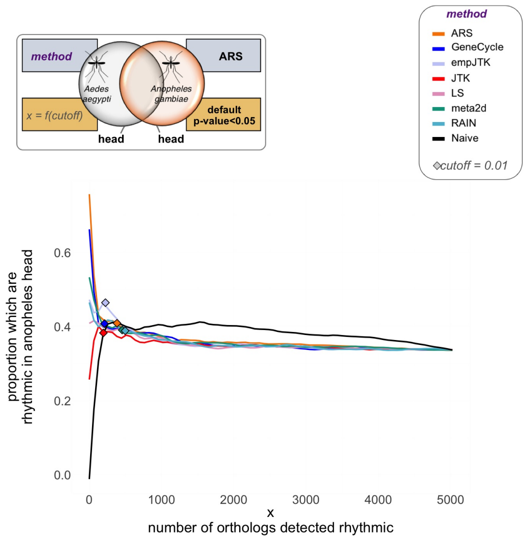

Fig. S19

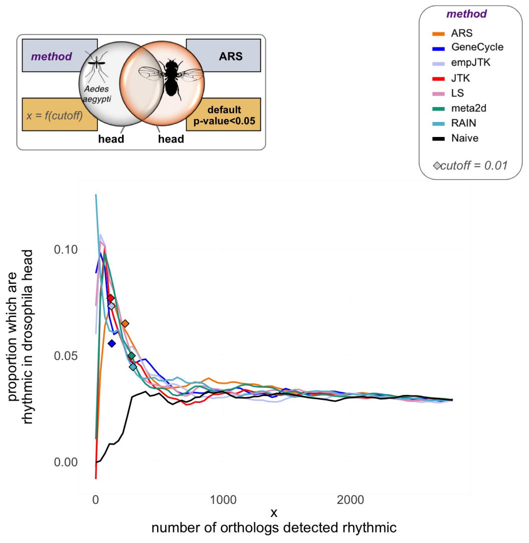

Fig. S20

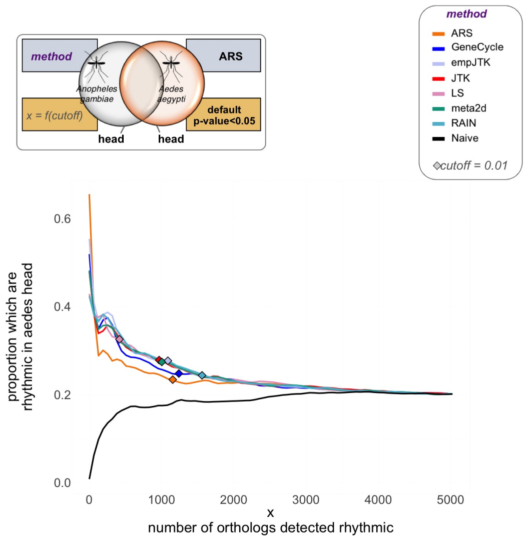

Fig. S21

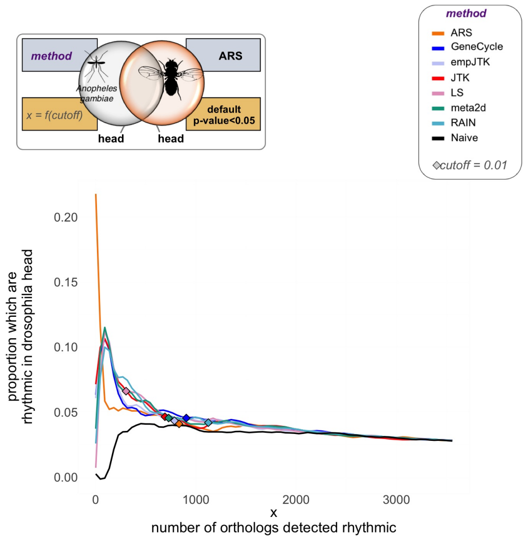

Fig. S22

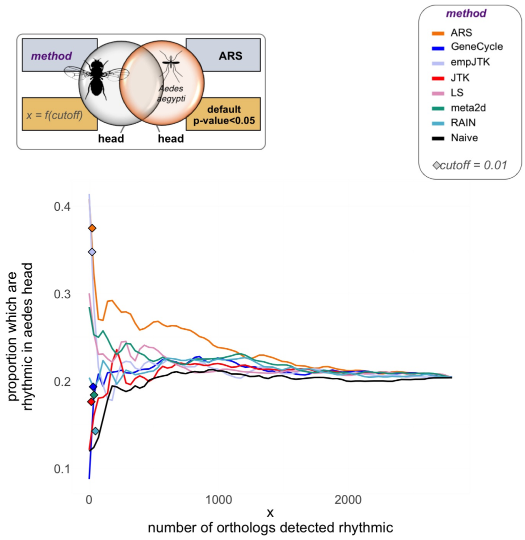

Fig. S23

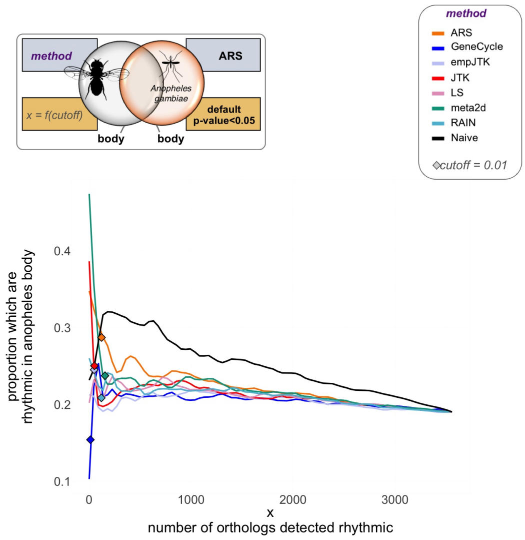

Fig. S24

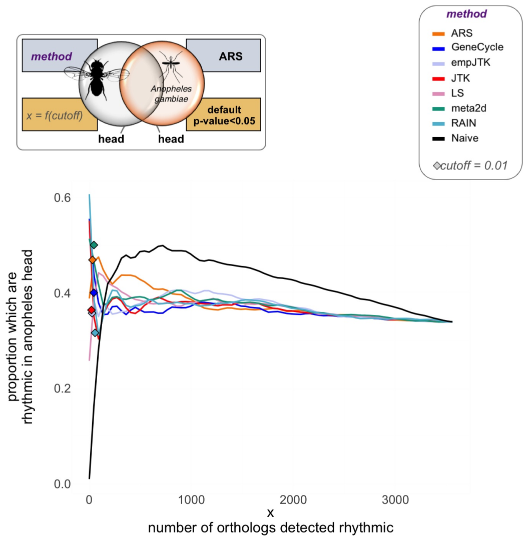
method
ARS
x = f(cutoff)
default
p-value<0.05
Anopheles gambiae
head
head
method
ARS
GeneCycle
empJTK
JTK
LS
meta2d
RAIN
Naive
cutoff = 0.01
proportion which are rhythmic in anopheles head
0.6
0.4
0.2
0.0
0
1000
2000
3000
x
number of orthologs detected rhythmic

Fig. S25

Images credit: Anthony Caravaggi (mouse), Ian Quigley (zebrafish) both license CC BY-NC-SA 3.0, Wikipedia GNU GPL Muhammad Mahdi Karim (baboon), and Public Domain for other images (from http://phylopic.org/)
