# Supplementary material for: Methods detecting rhythmic gene expression are biologically relevant only for strong signal
Source: PLoS Comput Biol. 2020 Mar 17;16(3):e1007666. doi: 10.1371/journal.pcbi.1007666 (PMC7100990; doi:10.1371/journal.pcbi.1007666)
Supplement: Attachment — Submitted filename: Review.docx [file pcbi.1007666.s008.docx]

**Reviewer #1**: Comments for PCOMPBIOL-D-19-01419
————————
This manuscript works to compare different rhythm detection methods using identification of orthologous rhythmic genes between species as a measurement of method accuracy. It identifies methods that perform better at this, and finds an association with expression level. It also re-enforces the previous findings that several of the methods do not have well-behaving p-values.

This thorough and extensive manuscript will be a beneficial addition to the circadian rhythm detection literature but would be significantly improved by addressing the major and minor issues which are within the scope of the paper, detailed below:

The manuscript can be primarily improved along these points

1) Clarification of the definition of strong signal

If I am not mistaken, the way you are defining “strong rhythmic signal” is just as genes with high expression. Why are you doing it this way and not using amplitudes? I can imagine a time series with high expression but small amplitude (1000 -> 1002 -> 1000 -> 998 -> 1000), and there are several papers with suggestions on how to define amplitude (Max - min, SD, Sqrt(2)*(Max-min) etc. I think this ends up making your argument a little misleading. If you mean “high expression genes” then say that instead of “strong rhythmic signal”.

There seems to be a misunderstanding. By “strong rhythmic signal” we mean genes with small p-values only. Even if strong rhythmic genes are enriched in highly expressed genes, that is not our definition. We did use a subset just of genes with high expression, but this was as a control, not as a signal of rhythmicity. We have improved the caption of Figure 6:

*Methods designed for rhythm detection in gene expression show an advantage only for the genes with a strong rhythmic signal, i.e. related to very small p-values.*

The rhythmic signal depends on how it is assessed by the method. In this revision, we have now investigated the relationship between this rhythmic signal force and the amplitude (Cf new figure in Additional file 1; Fig. S5), and indeed there is more signal detected within high amplitude genes.

Building on this, it is not clear to me why we should be concerned that rhythm detection methods are preferential to high-amplitude time series (that is, time series with a high signal(amplitude)-to-noise ratio. If anything, this seems like it would be preferable, and in fact methods like Bootstrap eJTK from Hutchison et al 2018 try to explicitly incorporate this.

If a method aims to detect all rhythmic genes, but only detects those with high amplitude, then it is a limitation. If a method aims explicitly to detect high amplitude rhythmic genes, and does that, then it is indeed fine.

We have added the following text to the Discussion:

*BooteJTK compares the noise to the amplitude of a time series, in addition to evaluating the rank order of the values, and thus might provide a more relevant rhythm detection by improving the variance estimation from biological replicates \cite{Hutchison3}.*

2) Though it is in your title, you do not actually define what you mean by “reliable”. Past definitions have used “able to distinguish signal from noise” as a proxy, or “able to identify the same gene when downsampled from the same biological experiment (Hutchison et al 2018) but here it seems you use “find genes rhythmic in one organism to have rhythmic orthologues in a different organism”. It is not clear that this gives more information about the differences or limitations of the methods or biological insight into differences between the organisms, tissues, or experimental conditions being compared. I think you need to make a stronger argument why we should expect the particular genes that you are comparing across organisms should be rhythmic, and the failure to identify them as such is a limitation of the methods as opposed to a interesting biological finding. As mentioned above, comparing across several organisms at once might be a way to strengthen this argument, or looking at “known rhythmic genes” such as the KEGG annotated ones (though see below for concerns about using these genes with the expectation that their expression profiles will be rhythmic).

We have changed the title to reflect a more general message:

"Methods detecting rhythmic gene expression are biologically relevant only for strong signal"

We have also replaced the word "reliable" with "relevant" everywhere in the text, as it is correct that we had not defined it well, nor do we truly test reliability.

*These results imply that even with a stringent p-value threshold, such as 0.01, the rhythmic nature of some of the genes considered rhythmic is not relevant.*

Concerning downsampling, we assessed the performance of methods according to their ability to detect rhythm signal and separate it correctly by producing an expected p-values distribution and being insensitive to down-sampling (Fig 3b). But indeed, this does not verify whether it concerns the same genes or not (also noted in remark n°5 of reviewer 4). Thus, we have added Fig 3c that shows that genes with small p-values tend to have also small p-values after down-sampling. We also added a sentence citing the BooteJTK method:

*Of note, for time-series with low sampling frequency, a recent improvement of empJTK, called BooteJTK, allows to detect rhythms robustly relative to sampling frequency \cite{Hutchison3}.*

Concerning our expectation of conservation between organisms, the only thing we expect is that a method which detects more genes with biologically relevant rhythmicity should also detect more conservation of rhythmicity. This is both justified in principle, because evolutionary conservation implies relevance to the functioning of the organism, and in practice, since orthologs rhythmic in one species have more chance to have a small p-value in another species (Fig 5d) (same homologous tissue).

We have added this justification to the Results.

3) Comparison across species only and not experimental design

While I think your use of orthologous genes in homologous tissues is an useful one, I think it is less useful when you confound the results comparing experiments done in LD vs. DD. While there should be an overlap for LD vs DD, the literature has already shown sufficient difference that making the comparison you have chosen with mouse DD vs rat LD weakens your results and your argument. I think your use of orthologous genes would be stronger if you used homologous organs in two different species where both organisms are tested under LD or DD conditions.

If you are going to compare across experimental designs and species simultaneously, then it might be interesting to look across more than two species at a time, as using that overlap among several species should mitigate some of the confounding from looking across experimental designs and be a stronger criterion on which to compare different methods.

The comparison between 2 species with one under LD and the other under DD should indeed produce less consistency, but we expect the order of more to less informative methods to be kept. We checked that there still be a consistency in the rhythmic signal between both conditions (LD versus DD) for a dataset for which we had both setups from the same experiment. We added Supp Fig. S14 and this information in the main text:

*Of note, the comparison of species under different conditions (light-dark versus dark-dark) is a limitation in itself since the overlap of the rhythmic transcriptome between these two conditions has been shown to be low \cite{Rund,Leming,Wijnen}. However, we found a good correlation of p-values obtained between these two conditions in the head of Anopheles gambiae (R=0.605, Additional file 1: Fig S14) suggesting that this limitation does not hide most of the conserved signal.*

4) Use and interpretation of p-values

I think something else to note here is that the expected proportion of overlap for these methods run on the same data is already between 60-70% (Hutchison et al. 2018), even within the same tissue, lab, and experimental collection. In your Figure 7, though, you show quite high levels of overlap. This discrepancy may be explained because you are not multiple-hypothesis correcting your rhythmicity results. This brings up a concern of its own. In lines 448-450 you mention that 0.01 might not be stringent enough of a cutoff. Why aren’t you multiple-hypothesis correcting in that case? Do your results change when you multiple-hypothesis correct?

Our results are robust to using multiple testing correction, as shown in Fig S15. We had forgotten to reference this in the main text, which was a mistake. We have now corrected that oversight and clarified this point:

*We made the same observation using an FDR-based threshold (FDR* ≤ *0.01 or FDR* ≤ *0.1, Additional file 1: Fig. S15b).*

Building on this, in your section at line 315 “The issue of significance” and “ARS, empJTK, and GeneCycle produce consistent p-values”, you correctly distinguish between the methods that generate correct p-values and those that don’t, but I think it might be worth discussing further the meaning and point of p-values as heuristics for rhythmicity. At a small scale, the overlap of the top 100 genes might vary widely, but be important because it will determine what individual genes to next conduct an experiment on, be it a knock out, or a new chronotherapy target. At that level, differences between the methods become important, and whether the gene has a 5% of being a false positive or 0.5% chance becomes very important. At a large scale, it might determine what biological processes to experimentally intervene upon, and the false positive rate becomes important there as well. I think merely ranking the order of genes misses this aspect of the rhythm detection methods.

Thank you for this interesting discussion. We have added FDR thresholds of 5% and 0.5% in two places to check this.

Sup. Fig S9 (Additional file 1) reproduces Fig. 4a with these thresholds, and shows that with a very low false positive tolerance all methods except LS overlap largely.

We have reproduced Fig. 6b highlighting the same thresholds (Fig S15a), which shows that even with such a stringent threshold as FDR 0.5% ortholog conservation of rhythmicity is weak for all methods. We didn't add this to the manuscript, as it does not add much information relative to the existing Fig. 6b.

Overall, it seems that the methods do not control well for false positive rates.

5) Alternative conclusions of your results

The title of your paper is “Methods detecting rhythmic gene expression are only reliable for strong signal”. I do not know if this statement can be distinguished from other statements that could be drawn from the same data, such as
- For genes with low expression, it is difficult to tell if their rhythmicity is conserved across species
- Rhythmicity is not conserved among genes with low expression.
- If orthologous genes have discordant expression levels, it is less likely that they will both be identified as rhythmic
- Methods detecting rhythmic gene expression agree more often for highly expressed genes

We agree that these different statements can be drawn from our results, and we have changed the title to reflect the general message:

"Methods detecting rhythmic gene expression are biologically relevant only for strong signal"

————————
Minor points:

Figure 1a:

I do not understand the placement of “Positive elements” as it seems to me it is next to an arrow that is a repressor

Indeed, we corrected this, thank you.

Figure 1b:

i) I don’t know what the goal of reporting the raw p-values is for JTK, meta2d, RAIN, and empJTK. It is clear from the design of the methods that they will all under-estimate p-values before correction, which you show, but I do not know what value to your argument or the discussion is added by having the raw p-values included in what is already a very busy (but very informative) figure.

The aim is to show that we don’t obtain the expected p-values distribution for JTK, meta2d, and RAIN, before or after correction, except for the corrected empJTK. It could have been argued that the distributions of corrected p-values were not expected to behave like standard p-values, so it is important to show also the behaviour of the uncorrected ones. Also this allows to show that the default correction allowed to empJTK to retrieve a uniform distribution under the null which was not the case for the 3 other methods.

ii) I think it would be worth including a subset image on the far right with a high right peak, to demonstrate the case where false negatives predominate and signal is discarded. It is arguably as important for a method to have a low false negative rate as a low false positive rate, and there are several examples in the figure (meta2d, JTK, RAIN) that show large right-sided peaks.

We do not know of a case which produces an expected theoretical distribution with a high right peak of p-values. Either the difference of the mean is 0 (no signal) so p-values are uniformly distributed, or it is not 0, meaning that there is a signal thus a skew towards small p-values. The only case we know of which can produce such a peak is a one-sided test on data which actually have a difference in the other direction than tested, as evoked in the text.

We have consulted statisticians, who confirmed that there is not a good theoretical expectation for such a right peak.

iii) Do we expect the RNA expression of all the ‘KEGG circadian entrainment’ genes to have rhythmic RNA expression? There are several cases where the protein levels are rhythmic and the RNA is not, cases where rhythmic protein modifications drive circadian involvement in genes that do not have RNA expression rhythmicity, and cases where protein levels are constant but the protein binds with a rhythmically expressed protein. All these cases would be indications for inclusion in in ‘KEGG circadian

No indeed we do not. But the 99 genes of KEGG circadian entrainment pathway for the mouse include obviously core clock genes which have rhythms at transcriptomic level and many genes which are known to have rhythms at transcriptomic level (in their RNA accumulation). So we expect these “KEGG circadian entrainment” genes to be enriched in having rhythms at RNA level, relative to all protein coding genes. That is why we presume that this set of genes constitutes an interesting group of genes to investigate the capacity of the methods to detect rhythmic signal in RNA accumulation.

We clarified this in the caption of the Figure 1:

*(99 genes from KEGG "circadian entrainment" among which we expect a large proportion of rhythmic mRNA accumulation.)*

Line 119:
You argue here that our analyses should not be biased towards high expressing time series, but is there i) a biological argument that these are more relevant or ii) an analytical argument that distinguishing strong signals that rise above the noise is an important thing to look for (the idea behind BooteJTK Hutchison et al 2018)?

It seems that we were not clear in the text on this, so we have clarified.

Our argument is not that analyses should not be biased towards highly expressed genes, since they clearly have more signal, but that (i) a method which is able to detect at least some lowly expressed genes as rhythmic is preferable, and (ii) a method should not detect almost all highly expressed genes as rhythmic. Added in the main text:

*We think that (i) a method which is able to detect at least some lowly expressed genes as rhythmic is preferable, and (ii) a method should not detect almost all highly expressed genes as rhythmic.*

Concerning signals above the noise, we have added a discussion of this recent paper improving empJTK:

*BooteJTK compares the noise to the amplitude of a time series, in addition to evaluating the rank order of the values, and thus might provide a more relevant rhythm detection by improving the variance estimation from biological replicates \cite{Hutchison3}.*

Figure 2a
i) The figure might be legible if instead of p-values you plotted -log10(p-values). If you’re trying to plot a uniform distribution it makes sense to deal with p-values in their original state, but for these scatterplots I think it would help delineate signal from noise.
Very good suggestion, thank you, we have modified the figure in this way (now Supp Figure S4a)

Figure 3
The caption should read “Fewer time points per cycle” instead of “Less time points”

Done (now Figure 2).

Figure 5c and 5d
After some time looking at the figures, I still don’t understand how if the “upset diagrams” are showing the intersection of genes in the top 6000 across methods, as you reduce methods I would expect you have to have higher numbers of intersection (more genes would be rhythmic in both empJTK and ARS than in empJTK, ARS, and LS, since some genes rhythmic in empJTK and ARS won’t be rhythmic in LS, and therefore excluded from that intersection). That I have put in a good effort to understand these plots and still cannot suggests that the explanation should be more explicit, or the data should be replotted.

Each intersection shows the interdiff() and not the intersection ; as illustrated in Figure 4a with the Venn diagram in which we see that ARS and GeneCycle for instance have 109 common genes that are detected rhythmic only by these two methods. If a gene is also detected rhythmic by another method, then it will not be indexed into ARS-GeneCycle intersection. It’s exclusive. That is why we add the Venn diagram to the first plot to help the understanding of Upset plots. We have also added a more precise description in the caption of Figure 4:

*Each intersection is exclusive, i.e. one gene can appear in only one intersection.*

Figure 6d

Is this distribution comparison the best way of comparing across tissues? I would expect a scatterplot comparing p-values for each orthologous gene would better support your argument for overlap or divergence of methods.

(Now Figure 5d)
We agree with this comment, and surprisingly we find no correlation. We have added Fig S12b and modified the main text:

*Similar results were obtained in other species comparisons (Additional file 1: Fig S13), with a stronger signal for evolutionarily close species such as mouse and rat (Additional file 1: Fig S12a), although we found no consistent correlations of the orthologs p-values between the rat and the mouse (Additional file 1: Fig S12b).*

294-295: Give X et al before citation in the same way you do in 297

Done.

412: This statement is untrue. empJTK accepts as inputs any time points as the header.

Thank you for this clarification, we have modified the text:

*Except for LS, RAIN, and empJTK, all other methods studied here assume equally spaced time-points.*

326: Are you saying we shouldn’t pay attention to rhythmic genes with low expression? Are there any biologically plausible reasons that genes that are not rhythmic across species would have high expression vs. low expression levels?

There must be a misunderstanding, the text around line 326 does not treat of expression levels, but of significance levels. By "strong rhythmic signal", we mean genes with small p-values. We just say that we don’t really know how relevant is the rhythmicity of genes with a weak signal of rhythmicity (0.01<p-val<0.05 for instance).

But, indeed rhythmic genes are enriched in high expressed genes. One explanation might be their expression cost (e.g. Wang et al. 2015 10.1016/j.celrep.2015.10.043).

**Reviewer #2**: Review – Methods detecting rhythmic gene expression are only reliable for strong signal.

In this manuscript the authors focus on the importance of identifying correctly the nycthemeral transcriptome or the rhythmic expression patterns of certain genes, which has been linked with relevant biological processes. The authors enumerate several methods commonly used to detect rhythmic genes and aim at benchmarking these different approaches (7 different methods) by evaluating their performance in real datasets compared to randomized ones and also by including homology context (evolutionary conservation). The study objective is twofold: define an approach to benchmark future methods based on biological data rather than simulated data, and provide recommendations on the design of time-series experiments as well as the choice of detection method.

The manuscript is nicely written, clear and detailed. The stated problem is relevant and clearly demonstrated in the statistical evaluation of the results (p-values distributions analysis) from the selected methods (JTK, LS, ARS, meta2d, empJTK, RAIN and GeneCycle), which indeed show different ability to detect true rhythmic genes.

A concern about the manuscript is that when the authors explain the benchmarking methods, it is not clear whether the methods are benchmarked for their ability to detect rhythmicity or their capacity to predict functionally relevant genes. The methods are built to perform the former and that should be the main criterion to evaluate them. The benchmarking approaches used are correct; however, the concern raised by the authors about identifying non-functionally relevant genes with rhythmic expression patterns is a general concern and not specific for these methods. Thus, this should be made clearer in the text.

We agree, we have added clarifications in the main text (INTRODUCTION):

*While the rhythm detection methods have been designed based on the rhythmic profiles of gene expression, rather than benchmarking them based on this rhythmic profile, we used the biological relevance of genes detected rhythmic. Notably we considered that orthologs which conserved their rhythmic expression can formed a suitable reference group of rhythmic genes.*

We have also updated the title to:

*Methods detecting rhythmic gene expression are biologically relevant only for strong signal.*

In more detail, there are several concepts that need clarification or further discussion:
1) Conservation of rhythmicity and biological relevance.
In several parts of the text, especially in the abstract, the authors seem to use conserved rhythmicity in orthologs as biological/functional relevance and unconserved rhythmicity as biological irrelevance.
For instance the authors write in the abstract: ‘In this study, we show that the nycthemeral rhythmicity at the gene expression level is biologically functional and that this functionality is more conserved between orthologous genes than between random genes’, ‘Rhythmic genes defined with a standard p-value threshold of 0.01 for instance could include genes whose rhythmicity is biologically irrelevant’.
The authors show indeed that rhythmicity is enriched among orthologs, which could indicate that this expression pattern is conserved for functional reasons. This has also been shown in other contexts (i.e. posttranslational modifications – functional sites) and can in principle be used as a way to prioritize these rhythmic genes. However, unconserved rhythmic expression patterns can also be functionally/biologically relevant. The authors mention this in the text: ‘It should be noted that this does not imply that we expect all rhythmic behavior to be conserved between orthologs’. However, this is somehow not reflected and clear in other parts of the manuscript. Even though the way the authors use homology to benchmark the methods is appropriate because a higher number of rhythmic orthologs is expected among true rhythmic genes, there is as well an unknown percentage of true positives among the unconserved rhythmic genes. This should be made clearer in the text. Also, it would very beneficial if the authors could provide some examples of conserved rhythmic genes with known relevant function (i.e. overlap with known cycling genes) and/or examples of clear false positives among the unconserved ones.

We have added the following text:

*An unknown proportion of the genes reported as rhythmic but not conserved will be true positives, whose rhythmicity evolved recently in one species or was lost in the other. This would only be a problem if a method would somehow favour these non-conserved ones while reporting true positives; we do not see any reason to expect such a behavior.*

2) Use of p-values. The authors compare the overlap of rhythmic genes detected by the different methods and observe that “the same genes seem to be called rhythmic by all methods but the threshold of significance appear(s) inconsistent. These results suggest an issue with the significance of p-value thresholds for the methods… Thus the methods agree on a large number of rhythmic genes, but not necessarily on the order of significance 210 among them”. The studied methods follow different approaches to detect rhythmicity, would the authors expect equal p-value ranking by all methods?

We agree with the reviewer that, since not all the methods are testing for the same pattern, they should not be expected to find the exactly same p-values or ranking, and that we should be clearer about this. Yet most methods do share a common pattern, i.e. sinusoidality. Moreover, all methods are described as retrieving "rhythmic" patterns, and ideally would be expected to produce very correlated results.

We have modified the text accordingly:

*Some methods are expected to produce different p-values because their underlying assumptions are different, i.e. other than sinusoidal for RAIN and empJTK. But the bulk of the methods are designed to find sinusoidal patterns and thus should ideally produce similar p-values, or at least similar ordering of results.*

Also, it is not clear whether the ‘issue with the significance of p-values thresholds’ is specifically an issue attributed to the studied methods or if this is more a common misuse of p-values (lower p-value, stronger evidence). Clarifying these points would help the reader, constrain the significance of p-values and at the same time benefit the argument of incorporating biological context.

While we agree that there are issues in science with the (mis)use of p-values, for a same dataset and a same question the most supported patterns should have the lowest p-values in a consistent manner. Thus, comparing the methods p-value output does measure their capacity to detect support for rhythmic patterns.

Moreover, all methods are meant to be used by considering their output of p-values with a defined threshold, and thus calling genes "rhythmic". This is indeed how they are used in the papers from which we have taken the data.

We have modified the text accordingly:

*While in principle effect size is sometimes more relevant than p-value, these methods are all used in practice to produce p-values, define a threshold, and provide a list of "rhythmic genes", thus consistency of these p-values is important.*

3) Known Cycling Genes. The authors show the distribution of p-values for known cycling genes for each of the methods; however, it might also be useful to see the number of those genes predicted as rhythmic by each method.

This number will depend on the threshold chosen. We wanted to compare the distribution of p-values when methods are applied to a group of known cycling and with the other distributions. (Also see reply Reviewer 1 Minor points: Figure 1b: iii) )

4) Access to the code. It would be very beneficial to have access to the code to reproduce the results or replicate them in other datasets or other methods. Could the authors make the code available in a repository (i.e. GitHub)?

We agree with the reviewer, and apologize for not putting the link in the previously submitted version of the manuscript: <https://github.com/laloumdav/rhythm_detection_benchmark>

(Added in the main text in “Availability of data and scripts”)

Minor changes

Page 3. Line 85. I would suggest changing the term ‘normal’ to refer to healthy/wild type individuals.

Done.

Page 4. Line 126. There is no description of the subset of known cycling genes in the Methods section. The authors should consider including a table with these genes in the Additional material.

It’s in Materials in “Cross-referenced gene IDs and known cycling genes”. Known cycling genes were obtained from KEGG database (or FlyBase for drosophila) and correspond to “circadian entrainment” or “circadian rhythm” pathways.

Figure 1a. The panel aims at explaining the concept of nycthemeral gene expression but even though it is meant to be a simplification, it is very convoluted and may benefit from a simplified flow and a less synthetic explanation in the figure caption.

We feel that it is not possible to further simplify without sacrificing accuracy.

Rhythmic genes are enriched in highly expressed genes. The number of detected rhythmic genes is enriched among highly expressed genes in all the benchmarked methods. This is not surprising and the authors clearly state this and provide some possible reasons. However, the statement would benefit from a statistical test showing the enrichment, similarly to how the enrichment of orthologs was demonstrated (page 8).

For the enrichment of highly expressed genes within rhythmic genes, this has already been shown in mouse and drosophila by Wang et al. 2015 (10.1016/j.celrep.2015.10.043). We have also added Table 2 which strongly support this result, and cited it in the main text:

*Our results support the hypothesis that rhythmic genes are largely enriched in highly expressed genes (Table 2).*

Figure 2a. Transforming the y-axis of the plot into the -log(default p-value) may help visualize better the enrichment of rhythmic genes among the highly expressed genes compared to the lowly expressed ones.

Excellent suggestion, thank you. (now Supp Fig. S4a in Additional file 1).

Figure 3, 4 Use of ‘every’ instead of ‘each’ to refer to all the individual time points registered.

Done (now Figures 2 and 3).

Figure 5a. The use of the Venn diagram is redundant with the Upset plot and could be removed.

Yes but this is for a better understanding of the upsets plots for people who would not be familiar with this type of plots (now Figure 4) (see also Reviewer 1 Minor points: Figure 4c and 4d); see also explanation added in the caption of Fig 4a.

In general in all panels, the colour scheme seems unnecessary and it just makes the diagrams more difficult to read. The authors should consider that the number of overlaps at all levels (methods) might not be as informative as having for instance the number of common predicted genes by all, by half of the methods or by more than two. This would simplify the visualization and convey a similar message. If the message is how similar the results from different methods are, the authors could consider using a Jaccard index heatmap for instance.

We disagree that the colours are unnecessary. We kept the same color for each method. We think important to keep Upset diagrams since it allows to show specific intersections between more than 2 methods. However, we also have provided a Jaccard index heatmap in Figure S7 (Additional file 1). This provides interesting additional information, thank you.

Methods. Missing values. There is no description of how missing values were handled (if any).

Thank you for pointing this out. We removed probsets which contained NA (added in pre-processing). Indeed, some methods cannot deal with missing values and that the comparison between methods for these probsets would therefore not have been possible.

We have modified the text accordingly:

*ProbIDs or genes which contained one or several missing values have been removed, allowing comparison between all methods even those which can not deal with missing values.*

**Reviewer #3**: I enjoyed reading the very well-written manuscript by Laloum and Robinson-Rechave. The authors have performed a benchmarking study of existing methods for detecting diurnal rhythms in gene expression. That might sound run-of-the-mill, but the analysis is especially interesting since it highlights p value distributions and evolutionary conservation. In all, a worthwhile study, definitely mature enough to be reported to the community.

I have no major criticisms, only more minor points:

Noting that the number of time points is crucial for detection power is almost trivial. RNA-Seq may be more sensitive, but I cannot imagine anyone expecting that it should outweigh cutting the number of time points by a factor 3 (Zhang et al.). I suggest shortening the report of those results.

This is not obvious in our opinion, since microarrays can fail to measure low expression levels, and generally have a much weaker dynamic range than RNA-seq. Moreover, while we do not have a published reference for this, oral presentation of the results and discussions with colleagues indicate that many researchers today expect RNA-seq to always out-perform microarrays, and thus that the point is relevant to many in the target audience.

The problem with non-uniform null pvalue distributions in original JTK could be corrected (empJTK). The same correction could be made to RAIN to produce "empRAIN", as suggested by Hutchison and Dinner in the cited bioRxiv report, with as they write "little additional effort". If this is true, authors might carry this out and include the results. Note (also the Editor please) that I'm not requiring this extra work. But the authors should in any case emphasize this in the "Limitations and improvement" section in the discussion, to stimulate such an effort.

We have added the following text in the Discussion:

*It is possible that the improvement of RAIN suggested by Hutchison and Dinner \cite{HutchisonAndDinner}, which allows to produce uniform p-values distribution under the null, might similarly improve the results of RAIN.*

Something that should be discussed is amplitude. Amplitude is closely related to function: High amplitudes are to most researchers in the field suggestive of (perhaps) more significant function. For most methods, high amplitudes should (for constant noise level) lead to lower pvalues. In fact, since pvalues are themselves random variables with very broad distributions (broader the lower the amplitude, typically), amplitude may be a strong filtering co-variate with higher potential than orthology to isolate "good" rhythmic transcripts. This has of course been noted earlier, see e.g., the "guidelines" JBR paper from 2017 or the original RAIN paper from 2014.

Thank you for the suggestion. We provide a new supp figure (Fig S5 in Additional file 1) showing the relationship between the amplitude and the rhythmic detection. Indeed, methods tend to detect rhythms for genes with high amplitude. But, we assume that even if methods detect less genes rhythmic among low amplitude genes, this does not prove that the rhythmicity of these latter genes is biologically less relevant than other rhythmic genes.

*Much more rhythmic signal is detected among genes with high amplitude (Additional file 1: Fig. S5). This does not necessarily imply that the rhythmicity of the low amplitude genes isn't biologically relevant.*

The broad distribution of pvalues and realizing that they are random variables themselves makes the results in Figure 5 quite expected: Venn diagrams by necessity then give a limited overlap for fixed cutoffs. This is something that was thoroughly discussed in the CMLS review by Lück and Westermark in 2016, which could be cited in relation to the Venn diagrams.

Lück and Westermark compared different tissues with the same method, so the biology is expected to differ, leading to many differences in detected genes. Conversely, our results of this figure (now Figure 4) are comparing the same samples with different methods, thus the biology is the same, and a strong overlap would be expected if the methods worked perfectly.

Related, regarding the analysis in Figures 6 and 7. It might be interesting to discuss whether rhythmic orthologs tend to have higher amplitudes, leading to a tendency for lower p values, and thus to detection by more methods.

See answer about amplitude; now Figures 5 and 6.

The reporting of the results in Figures 6 and 7. is a bit of a mess to follow. The reader must be helped better to see the essential point. It is no trivial analysis, but the main text might be shortened to focus on the essentials (mainly Figure 7b). Explaining the analysis thoroughly could be done only in the methods section.

We moved some technical details to the Methods. We feel that for the section "Only strong rhythmic signals of gene expression are relevant" it is important to keep detailed information, as this ortholog reference approach is easily misunderstood. These are now Figures 5 and 6.

Page 3 close to top: To be fair, original JTK could detect an arbitrary waveform with a slight code modification, as stated in the original JTK paper. The limitation is rather that only this one waveform will be detected (a sine curve by default).

Indeed, that’s true, we added this precision, thank you for pointing it out.

*empJTK and RAIN aim to improve the original JTK algorithm which assumed that any underlying rhythms have symmetric waveforms (more precisely, only the waveform coded into the JTK algorithm will be detected, which is the sine curve by default) \cite{Thaben}.*

On page 7 3rd row from bottom: "efficieny", is "statistical power" meant?

Thank you for the remark, the correction was made (Figure 3 now).

**Reviewer #4**: There have been several reviews on this subject.
This manuscript adds to the existing literature
It includes more recent algorithms and, admirably, attempts to move beyond synthetic data (with its inherent biases) in the evaluation.

On these fronts the authors should be commended.

I do, however, have several significant concerns (Some more significant than others ;)
Some of the conclusions are overstated
There seem to be a few statistical issues/inconsistencies
One of recommendations seems wholly unsupported (and I would argue wrong)
Still I suspect that authors can address all my concerns

(1) In ALL P value histograms – you show p values less that 0 (the distribution goes to the left of the green 0, about -.2) and greater than 1. (the distribution goes to ~ 1.2) I am guessing/hoping this is a mislabeling? Did you try to do some weird kernel smoothing of these distributions that didn’t reflect their constrained domains?

This is just due to smoothing in the ggplot R package for density. All the precise p-values are available on our GitHub (<https://github.com/laloumdav/rhythm_detection_benchmark>), and never go outside 0 to 1. We added the GitHub link in the main text in “Availability of data and scripts”.

(2) The significance thresholds used throughout this manuscript are all very low and not reflective of current best practices (see for example the review by Hughes et al the authors site) In the setting of testing ~ 20,000 genes. A p value threshold of 0.01 is likely to be loaded with false positives. The analysis needs to be re-done with a reasonable threshold (q<=.1?) as including multiple test corrections is both important and standard.

Our results are robust to using multiple testing correction, as shown in Fig S15. We had forgotten to reference this in the main text, which was a mistake. We have now corrected that oversight and clarified this point. Thanks for pointing out this.

*We made the same observation using an FDR-based threshold (FDR* ≤ *0.01 or FDR* ≤ *0.1, Additional file 1: Fig. S15b).*

(3) The initial analysis showing that the p-value distributions resulting from the application of some of these methods on randomly generated data -is nonuniform seems important. (A) But in the end your argument that this is important is significantly undercut by your eventual use of the p-values as a simple ranking method. If we are just using the numbers for ranking purposes (which on the face of it – I don’t think I would advocate) then the fact that the p values don’t follow a uniform distribution wouldn’t be a deal breaker. You should either explain this – and consider ALL the methods throughout the review , stick to a reasonable p or q value threshold, or provide some reasoning for both excluding the methods based on distributional properties that you ultimately neglect.

The only place where we did not consider all methods is Figure 3, where we investigated the impact of replicate sampling. Notably, in all comparisons where we ranked results according to p-value we considered all the methods.

Even if we work with ranked p-values as output after applying methods to real data, this does not change the fact that some methods do not have a uniform distribution under the null while they should. We included these methods in the benchmark, but we do consider that this is a strong argument in their disfavour in the Discussion, and we do report their results less in detail in the text (but they are included in the figures 4-6).

We discuss the limitations of apparently reasonable thresholds for p-value or FDR in the context of these rhythmicity methods in several places, for example:

*These results imply that even with a stringent p-value threshold, such as 0.01, the rhythmic nature of some of the genes considered rhythmic is not relevant. These rhythm detection methods were relevant only for genes with very high signal of rhythmicity, where they performed better than a Naive method.*

(B) More theoretical point – the formal argument is that p values should be uniformly distributed under the “null hypothesis” I am not an expert in all these methods. What are thir null-hypthesis with regard to the temporal pattern? No variation? Gaussian white noise? Autoregressive noise? What is the statistical distribution of your random data? Does it match the null hypotheses for these different methods?

It is difficult to find explicit null hypotheses in each of the original method papers, but it is clear that randomizing the data relative to timepoints should remove all signal, and thus that there should not be any departure from a reasonable null.

(4) You spend a few pages separating data into high/low expression genes – and then mean/variance normalizing gene expression (within a gene) to test if that would influence the identification of cycling genes. But a quick review shows me that several of these methods (e.g JTK, eJTK, GeneCycle) are rank based. (purely use rank of the sample’s expression of Gene X compared to all other samples). By construction these methods should be completely unaffected by your normalization process (which is what you find). Thus these numerical experiments unnecessarily lengthens the manuscript. It seems like you could quickly explain this and then note that the bias toward High expression is not an artifact of (at least those) methods. It either reflects true biology or a lower signal to noise ratio in lowly expressed gene

Indeed. We put the Figure 2 in Supp Figure (Additional file 1: Figure S4) and shortened the text.

(5) Figure 4B Analysis of reducing number of data points and comparison of fewer data points over 2 cycles vs more over 1 cycle. You only show the distributions of p values here. This seems insufficient. You could theoretically be identifying completely different genes (with the reduced data sets) as compared to the full data set. Ie (assuming the full data set is a better representation) – the significant genes in the reduced data set could be all false positives. Similarly when you GeneCycle is not influenced by this comparison – you don’t really know that, You have only shown that it picks out the same # of genes – not the same genes.

Indeed, we added now Figure 3C showing that the same genes with small p-values tend to have also small p-values after down-sampling. Thank you for the suggestion.

(6) You repeatedly find that the baboon data set does not seem to have much overlap with the mouse (or other data sets). In reviewing this study I find that is has **very low read depth** – complicating any quantitative analysis. You might want to raise this possibility? (up to you)

Thank you for the observation. There are several issues with this baboon dataset, but we prefer not to focalize the manuscript on this point, as it would distract from the main message, and our results are confirmed with various datasets. At the same time, it would be odd to not use at all what is the largest such dataset published so far.

(7) Final Recommendations “Consider biological replicates as new cycles with one replicate” Perhaps I misunderstand your point - I think this is simply wrong. I can’t find that you supported this (one way or another) in your analysis. More to the point there is a big – real world – difference between euthanizing 3 mice every few hours for a day… and doing 1 mouse for three days. In the first case a single/random unknown influence of the shared environment (e.g. there was a loud noise at 12 pm stopping the mice from eating) would influence all three mice euthanized at that time. Concatenating their data as if they happened in 3 consecutive days would make this appear like a rhythmic behavior (a daily peak near noon). Having 3 days of real data would minimize this risk (unless the noise repeats every day at the same time) There are almost always unknown perturbations – this suggestion is a recipe to make them all appear rhythmic.

In our case, it concerns tissues such as the liver or the lung for which we need to sacrifice the individual in order to harvest the tissue. Each data at a given time-point requires the sacrifice of one individual. So to get 3 biological replicates per time-point over 1 cycle or 1 biological replicate per time-point over 3 cycles, this requires the sacrifice of the same number of individuals.

Also see our “ethical considerations” on this point.

But indeed there is the case of “single/random unknown influence” you describe where a rhythmic process could be retrieved. We now clarify this in the main text:

In the Discussion:

*Thus, if only one 24h cycle is feasible, ~~one cycle with~~ several biological replicates ~~should~~ must be favoured.*

[…]

*Moreover, we recommend producing data over at least two cycles to be sure of the repetitive nature of profiles, and to avoid a potential random influence of the shared environment, which might be considered rhythmic since it affects all replicates.*

[…]

In Recommendations:

*One full period sampled is the minimum required. Two periods are to be preferred.*

(8) Throughout the text you refer to conserved cycling as biologically important. (and non-conserved cycling to be unimportant). I understand that you are trying to use evolutionary conservation. But this is a giant oversimplification. The cycling of a gene – unique to a particular animal – might be very important to that animal. Conserved cycling of a transcript – that has a very long half-life protein product – might be completely physiologically unimportant. This language needs to be cleared up throughout. At best could say something “presumed evolutionary importance” but even this is too strong. I leave it to the authors to find some less loaded terminology
But the terminology should be refined

Non-conserved cycling are not considered as unimportant but we expect that a method which detects more genes with biologically relevant rhythmicity should also detect more conservation of rhythmicity. This is both justified in principle, because evolutionary conservation implies relevance to the functioning of the organism, and in practice, since orthologs rhythmic in one species have more chance to have a small p-value in another species (Fig 5d).

We provided modifications/precisions in main text:

In the Introduction:

*An unknown proportion of the genes reported as rhythmic but not conserved will be true positives, whose rhythmicity evolved recently in one species or was lost in the other. This would only be a problem if a method would somehow favour these non-conserved ones while reporting true positives; we do not see any reason to expect such a behavior.*

[…]

*While the rhythm detection methods have been designed based on the rhythmic profiles of gene expression, rather than benchmarking them based on this rhythmic profile, we used the biological relevance of genes detected rhythmic. Notably we considered that orthologs which conserved their rhythmic expression can formed a suitable reference group of rhythmic genes.*

[...]

In the Results:

*We expect that a method which detects more genes with biologically relevant rhythmicity should also detect more conservation of rhythmicity. This is both justified in principle, because evolutionary conservation implies relevance to the functioning of the organism, and in practice, since orthologs of rhythmic genes have smaller p-values (Fig 5d).*

(9) For figures 6 and 7 I am a bit confused. In these comparisons was the analysis in both species done using the same method. (ie e-JTK defines cyciling in the rat liver – and then e-JTK is used to asses cycling in mouse liver). This seems like the more fair test for each method. But in reading the text it seemed like you used GeneCycle to find (for example) the rhythmic genes in the “benchmark” rat set. and then used all the other method in the lung and looked for overlap. If that is what was done --- it should be redone to be fair (but I doubt it will change the results much). If the “fair” test is the one that was already done – the text should be clarified.

Yes, we provided this information in Additional file 1: Fig S11. We also added Figure S12b (in Additional file 1) showing that orthologs rhythmic in one species have more chance to have a small p-value in another species (same homologous tissue). Note that figures 6 and 7 are now figures 5 and 6.

(10) The analysis of conservation – while important – is also overstated.

You postulate (A) “Biologically important cycling should be conserved across species” and then jump to (B)“The statistical test that identifies a property most likely to be conserved is the best test. (B) does not follow from (A) For example A test completely unrelated to cycling could pick out a feature that is more likely to be conserved. This does not make it a good cycling test. Indeed this (I think) what you find when you say the “Naïve test” is better. High expression is actually a more phylogencially conserved feature than cycling per se. More to the point this test is assessing a desirable side feature of a cycling evaluation algorithm – but not its main function. As such this is an important – but secondary issue. Again the discussion needs to be d reframed and toned own (Limitations better acknowledged)

We agree that this is what the "Naive test" shows. But we also maintain that there is a large body of evidence that functional features are more evolutionarily conserved than random. The logic is: (A) functional cycling should be more conserved across species than random; (B) a good method for rhythmicity detection should detect more functional cycling than random; thus a good method for rhythmicity detection should be enriched in cycling conserved between species, relative to random.

Is this more clear?

(11) No mention is made of the precision of the different methods in describing the particulars of a cycling behavior (e.g the amplitude or period or some other feature depending on how the waveform is described. This is often of great import. Particularly as more recent experiments are trying to assess if there is a change in these parameters with a stimulus. These factors should be considered. It looks like some of the earlier reives did this – but as one of the points of this review is to look at newer methods – it should be redone. This might make a particular differenc with regard to questions of using more replicates (or adding time points) in two a smaller number of cycles – or measuring more cycles (eg. 2 animals every hour for 2 day) vs 2 animals every 4 hours for 4 days)

Here we are benchmarking the capacity to correctly classify genes are rhythmic or not with a period of about 24h. We noted in Limitations and Improvement that we did not assess the amplitudes, phases, and precise period provided by the algorithms. However, we added Fig S5 (Additional file 1) which shows the relationships between the amplitude and the rhythm detection (added in the main text).

Moreover (i) we lack good benchmarking criteria for these features, and (ii) we need to first be able to detect rhythmicity before characterising its detailed features. Our results show that this is not yet obvious.

(12) You try to squeeze a lot on the figures. Perhaps I am just to old - but when printed out I find them to be unreadable.
Text and plots need to be bigger. Perhaps plot a bit less on each figure (if thats what you need to do)

We apologize for the inconvenience. We have added all sub-figures as full size original figures in a folder "Paper main figures" on the GitHub of the paper (<https://github.com/laloumdav/rhythm_detection_benchmark>)
