# Supplementary material for: Methods detecting rhythmic gene expression are biologically relevant only for strong signal
Source: PLoS Comput Biol. 2020 Mar 17;16(3):e1007666. doi: 10.1371/journal.pcbi.1007666 (PMC7100990; doi:10.1371/journal.pcbi.1007666)
Supplement: Attachment — Submitted filename: Review_2nd.docx [file pcbi.1007666.s009.docx]

**Reviewer #2:** The authors have responded all the comments and edited the manuscript with the appropriate changes.

**Reviewer #3:** I am still endorsing this paper and am generally happy with the revisions. A careful re-reading revealed the following minor points.

Density estimates for p value distributions go beyond the support for the p values, i.e. have mass at < 0 and > 1. Density estimates can easily be restricted to 0 <= p <= 1, which should be done.

Done

Re: Figure S12b. The main text correctly mentions the lack of consistent p value correlation, but the naive reader may be mislead by the astronomically low p values. This may be related to the KS tests e.g. Figure 5d: the KS test is sensitive to the most minute differences in distributions: even very very similar distributions with minuscule differences are often detected by this test. Thus, some kind of deviance measure to assess similarity of the distributions would be beneficial here.

We now provide D statistic values given by KS tests, and mentioned them in the main text; they are not that "minute":

*(with Kolmogorov's D statistic around 10-15% of maximum deviation, Fig 5d)*

*(with Kolmogorov's D statistic around 10-15% of maximum deviation, Additional file 1: Fig S12a)*

Use of Pearson correlation coefficients: Especially for Figure S12 it seems important to use e.g., a permutation test to assess significance since the log p values cannot be assumed to follow a normal distribution. Was this done?

We added Spearman correlation coefficients in figures S12b and S14, which are consistent; Spearman correlation does not have an assumption of normality of the data, only that they are ordinal.

New text "Of note, the comparison of 260 species under different conditions (light-dark versus dark-dark) is a limitation in itself 261 since the overlap of the rhythmic transcriptome between these two conditions has been 262 shown to be low":
Where has it been shown to be low? In fact I even discourage the use of the term "overlap" which is arbitrary and can be misleading; overlap is usually defined by arbitrary cutoffs. It introduces a black/white thinking into a highly greyscale reality. In any case, mouse liver LD/DD were compared in the DODR paper and the correspondence did not seem "low".

The 3 references were hidden by the highlighting; we apologize for the LaTeX error.
For instance, the first reference (Rund et al. 2011) says: “*an important finding from our analysis in* An. gambiae *is that the overlap in genes rhythmically expressed under both LD and DD conditions is limited to ∼60%*” (our emphasis). The second reference (Leming et al. 2014) says: “*secondly, there is limited overlap of genes rhythmic under both conditions (24% and 28% as proportions of LD or DD rhythmic genes)”*.
We have added a clarification in brackets:

*Of note, the comparison of species under different conditions (light-dark versus dark-dark) is a limitation in itself since the overlap of the rhythmic transcriptome between these two conditions has been shown to be low (although this interpretation remains limited by the thresholds used).*

The caption of Figure S5 mentions 7 methods, only 5 are shown.

Indeed, only 5 methods produced estimations of amplitude. We corrected the text and added the following clarification:

*Only five methods are shown since they are the only ones giving estimations of amplitude.*
